# Supplementary material for: Comprehensive genomic analysis of dietary habits in UK Biobank identifies hundreds of genetic associations
Source: Nat Commun. 2020 Mar 19;11:1467. doi: 10.1038/s41467-020-15193-0 (PMC7081342; doi:10.1038/s41467-020-15193-0)
Supplement: Supplementary file 1 — Supplementary Information [file 41467_2020_15193_MOESM1_ESM.pdf]

Supplementary Information

Comprehensive genomic analysis of dietary habits in UK Biobank identifies hundreds of associated loci

Cole et al

Supplementary Information Table of Contents

Supplementary Figure 1: Phenotype correlation of 85 single FI-QTs	3
Supplementary Figure 2: Principal component analysis percentage of variance explained (scree plots)	4
Supplementary Figure 3: SNP heritability	5
Supplementary Figure 4: Significantly heritable PCs and their contributing FI-QTs	7
Supplementary Figure 5: Relationship between SNP heritability and GWAS success	37
Supplementary Figure 6: Association of lead SNP P-values across all dietary habits with P<0.05	39
Supplementary Figure 7: LocusZoom plots	42
Supplementary Figure 8: Bidirectional Mendelian Randomization with PC1 as the exposure and outcome with educational attainment, intelligence, and BMI	48
Supplementary Figure 9: P-value comparison of significant SNPs between two alcohol related phenotypes	51
Supplementary Figure 10: Correlation matrix of alcohol-related traits	52
Supplementary Figure 11: Heritability estimates between BOLT-lmm pseudo-h2g and LD Score regression heritability estimates	53
Supplementary Table 1. Bidirectional Mendelian randomization results for PC1 after SNP filtering	54
Supplementary Table 2. Mendelian randomization validation and sensitivity results for educational attainment and PC1	55
Supplementary Table 3. Mendelian randomization results between educational attainment and PC1's contributing FI-QTs	56
Supplementary Table 4. Bidirectional Mendelian randomization results for diet and coronary artery disease (CAD) and type 2 diabetes (T2D)	57

Supplementary Figure 1: Phenotype correlation of 85 single FI-QTs. Absolute pairwise Pearson correlation matrix of 85 QT derived from 35 FFQ clustered manually by related FFQ questions and food groups. While absolute correlations are presented visually herein, raw correlation values ranging from -1 to 1 are presented in Supplementary Table 2. Correlations with non-significant P-values ($P > 0.05/85 = 5.88 \times 10^{-4}$) were set to 0 and are white on the corresponding figure.

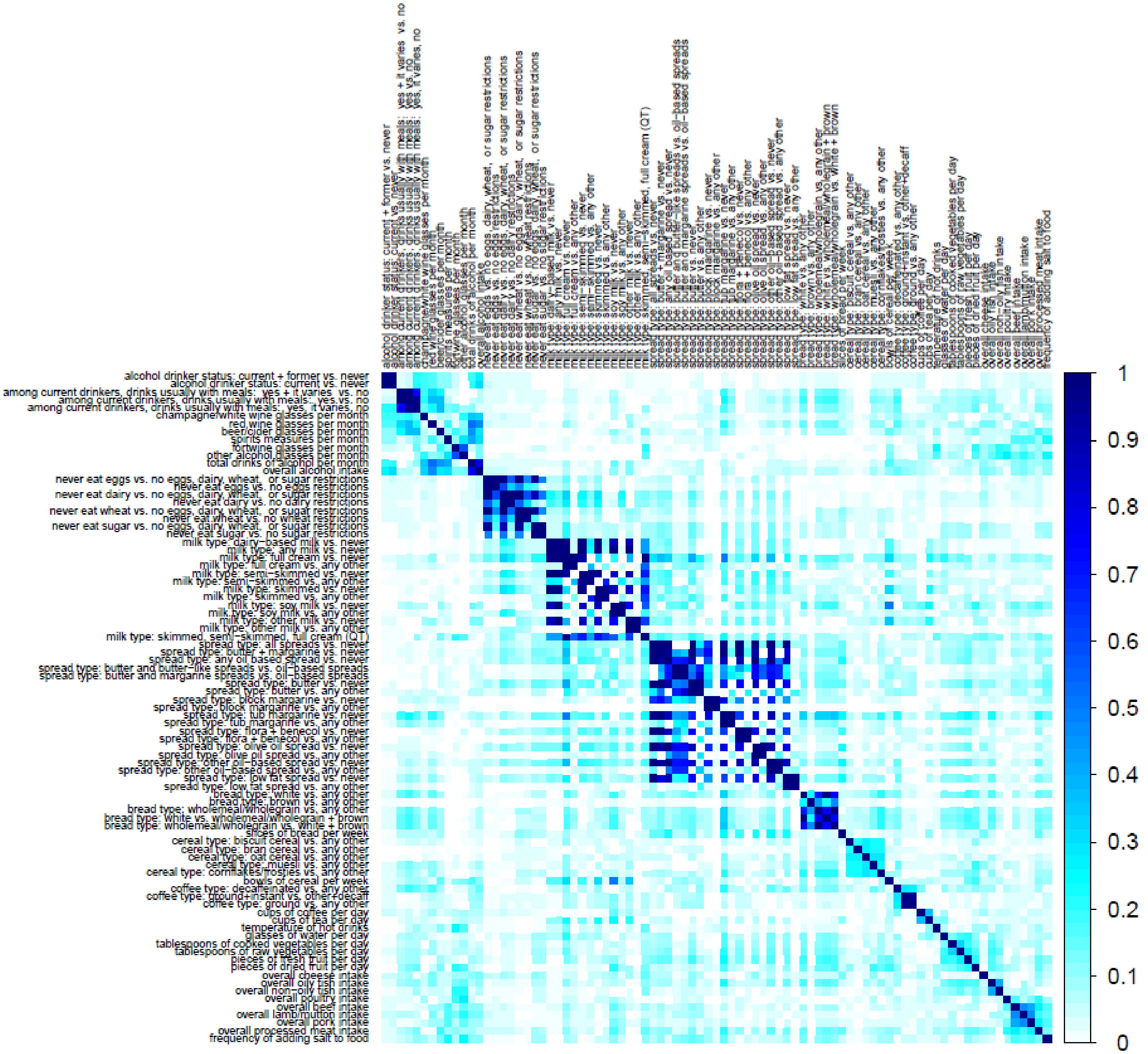

Supplementary Figure 2: Principal component analysis percentage of variance explained (scree plots). A) Percentage of variance explained by each of 85 PCs derived from PC analysis on 85 single FI-QT. B) Percentage of variance explained by the top 20 PCs with eigenvalue greater than one.

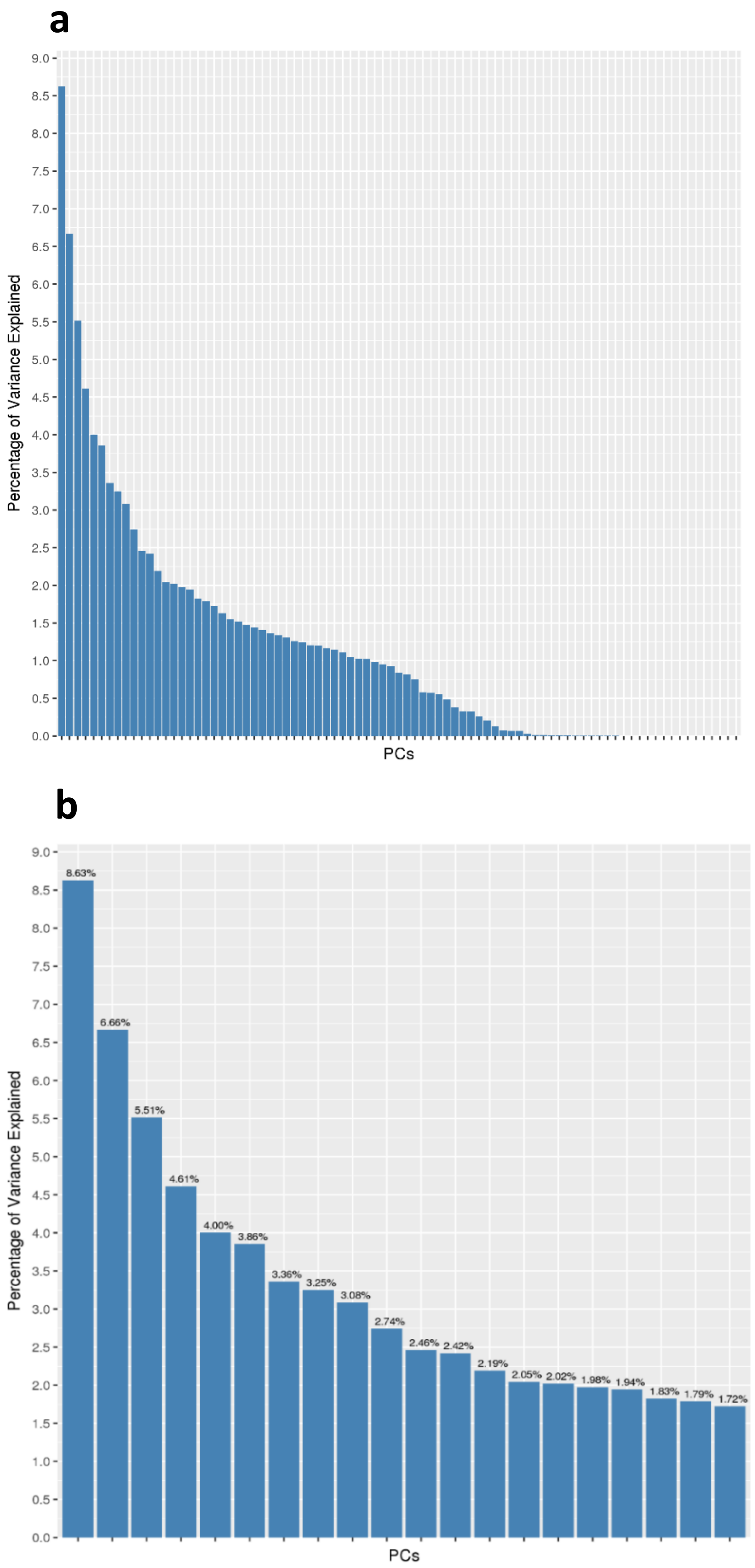

Supplementary Figure 3: SNP heritability. A) Boxplots of SNP heritability are shown for significantly heritable dietary habits from each of three categories: single FI-QTs, PCs, and PCs with eigenvalues (EV) greater than one (PC1-20). B) SNP heritability vs. PC order of the 60 significantly heritable PCs labeled with phenotypic variance explained. (boxplot elements: center line, median; box limits, upper and lower quartiles; whiskers, 1.5x interquartile range; points, outliers)

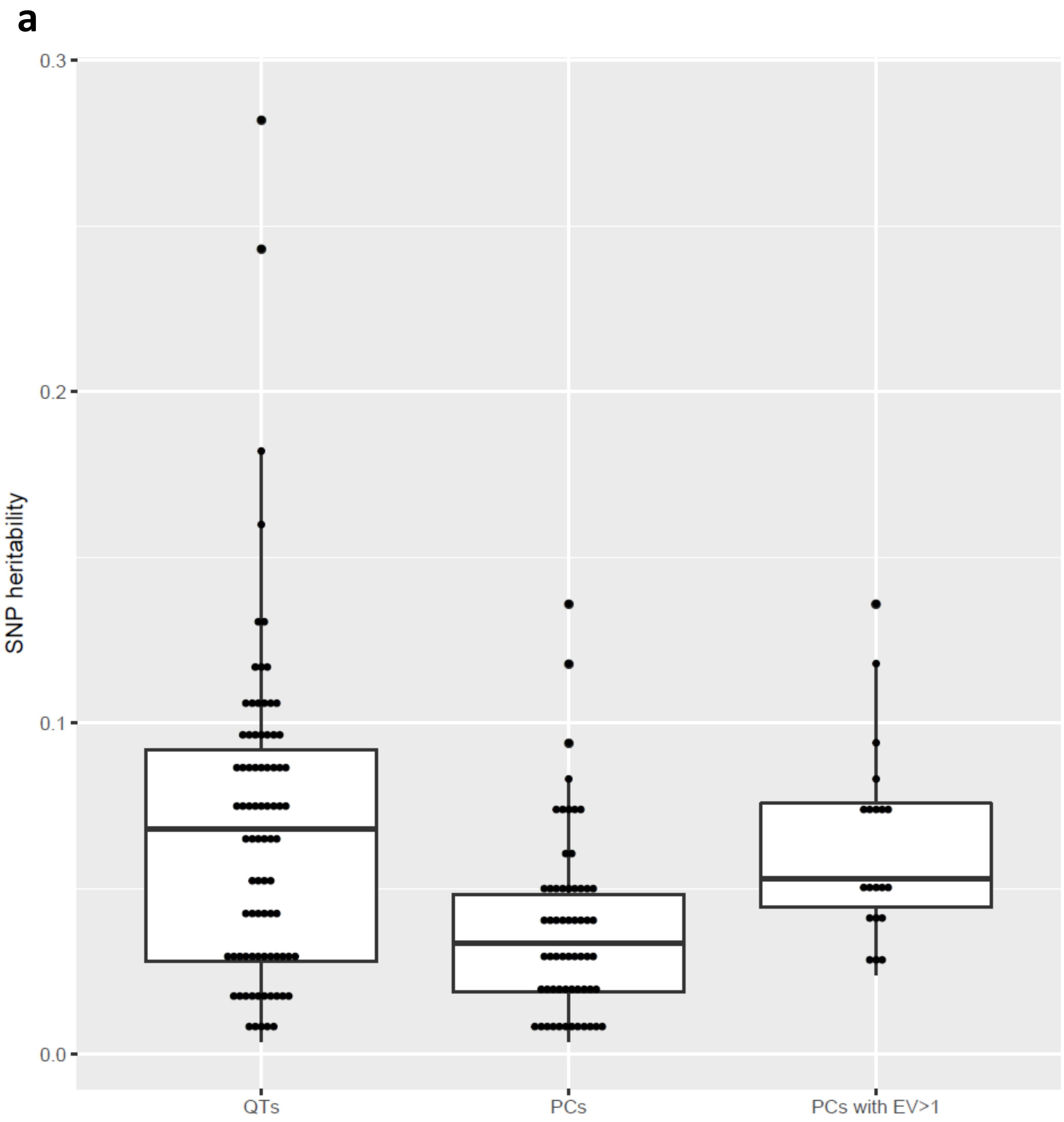

b

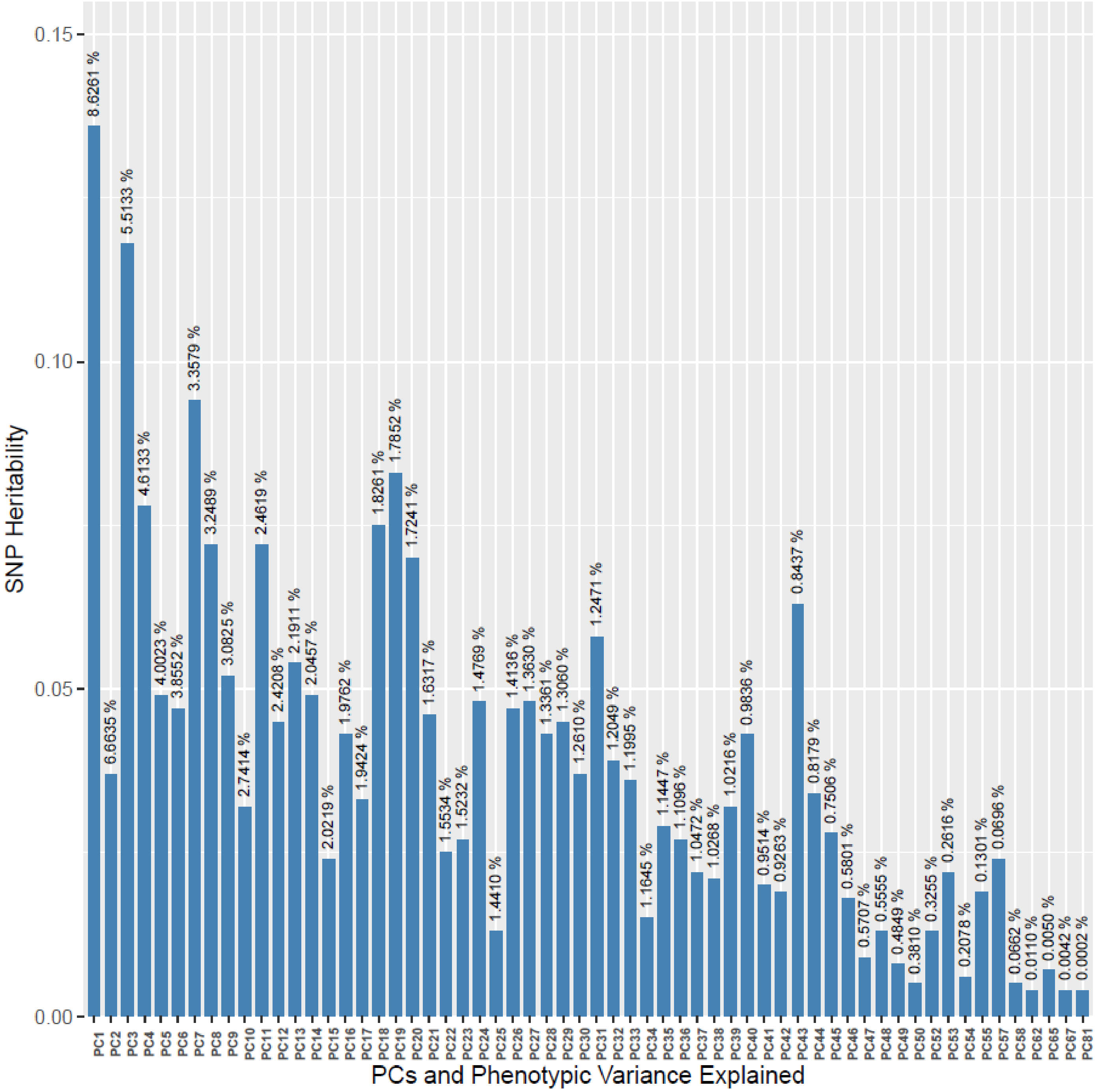
SNP Heritability
0.15
0.10
0.05
0.00
8.6261 %
6.6635 %
5.5133 %
4.6133 %
4.0023 %
3.8552 %
3.3579 %
3.2489 %
3.0825 %
2.7414 %
2.4619 %
2.4208 %
2.1911 %
2.0457 %
2.0219 %
1.9762 %
1.9424 %
1.8261 %
1.7852 %
1.7241 %
1.6317 %
1.5534 %
1.5232 %
1.4769 %
1.4410 %
1.4136 %
1.3630 %
1.3361 %
1.3060 %
1.2610 %
1.2471 %
1.2049 %
1.1995 %
1.1645 %
1.1447 %
1.1096 %
1.0472 %
1.0268 %
1.0216 %
0.9836 %
0.9514 %
0.9263 %
0.8437 %
0.8179 %
0.7506 %
0.5801 %
0.5707 %
0.5555 %
0.4849 %
0.3810 %
0.3255 %
0.2616 %
0.2078 %
0.1301 %
0.0696 %
0.0662 %
0.0110 %
0.0050 %
0.0042 %
0.0002 %
PC1
PC2
PC3
PC4
PC5
PC6
PC7
PC8
PC9
PC10
PC11
PC12
PC13
PC14
PC15
PC16
PC17
PC18
PC19
PC20
PC21
PC22
PC23
PC24
PC25
PC26
PC27
PC28
PC29
PC30
PC31
PC32
PC33
PC34
PC35
PC36
PC37
PC38
PC39
PC40
PC41
PC42
PC43
PC44
PC45
PC46
PC47
PC48
PC49
PC50
PC52
PC53
PC54
PC55
PC57
PC58
PC62
PC65
PC67
PC81
PCs and Phenotypic Variance Explained

Supplementary Figure 4: Significantly heritable PCs and their contributing FI-QTs. Similar to Figure 1, the following figures depict the phenotypic (upper triangle) and genetic (lower triangle) correlation between each PC's significantly contributing FI-QTs with each other and with the PC. All correlations with nonsignificant p-values ($P > 0.05/85 = 5.88 \times 10^{-4}$) were set to 0. Percent contribution of each of the contributing traits to the PC is depicted in the correlation matrix bar plot annotation, colored by loading direction.

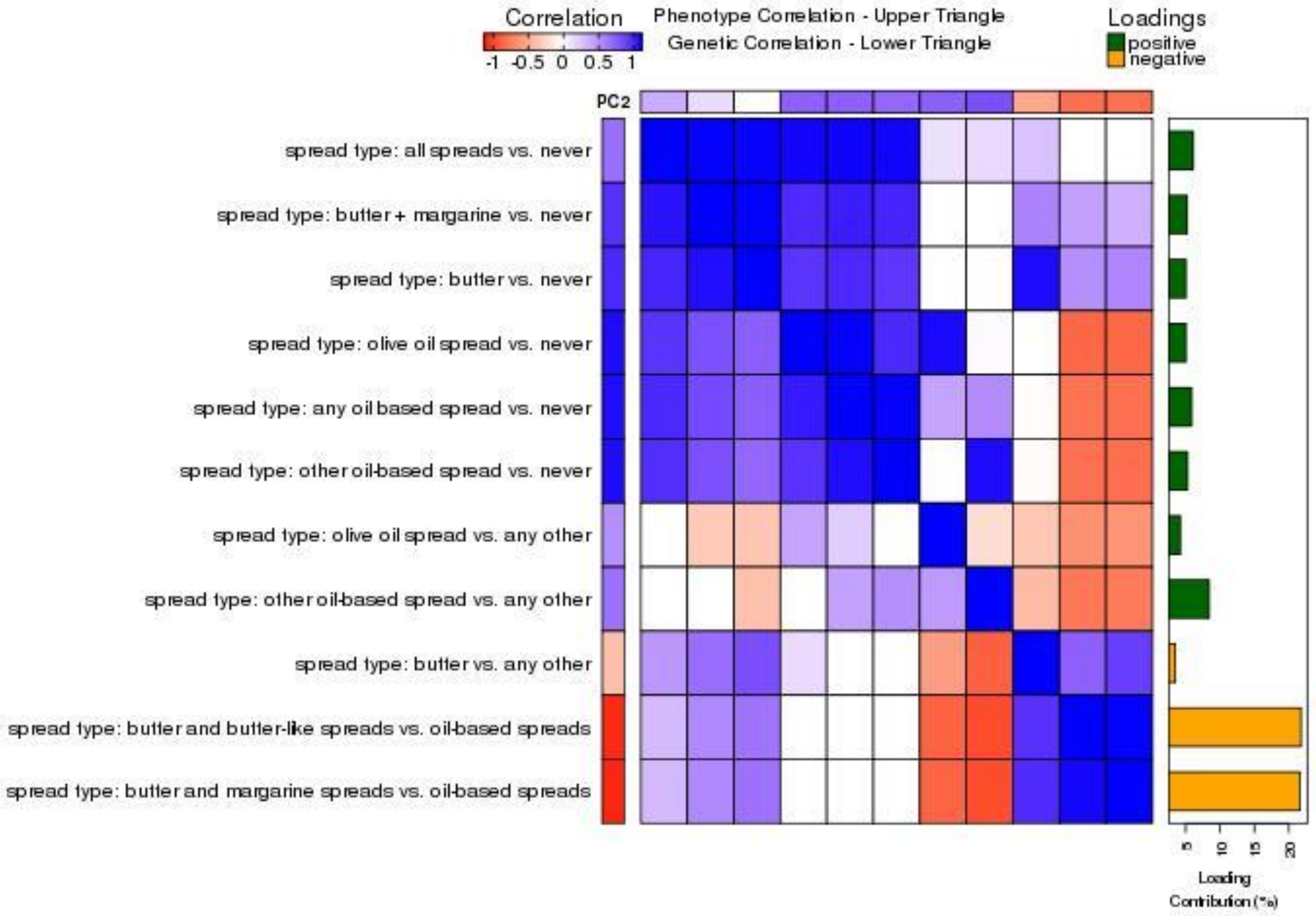

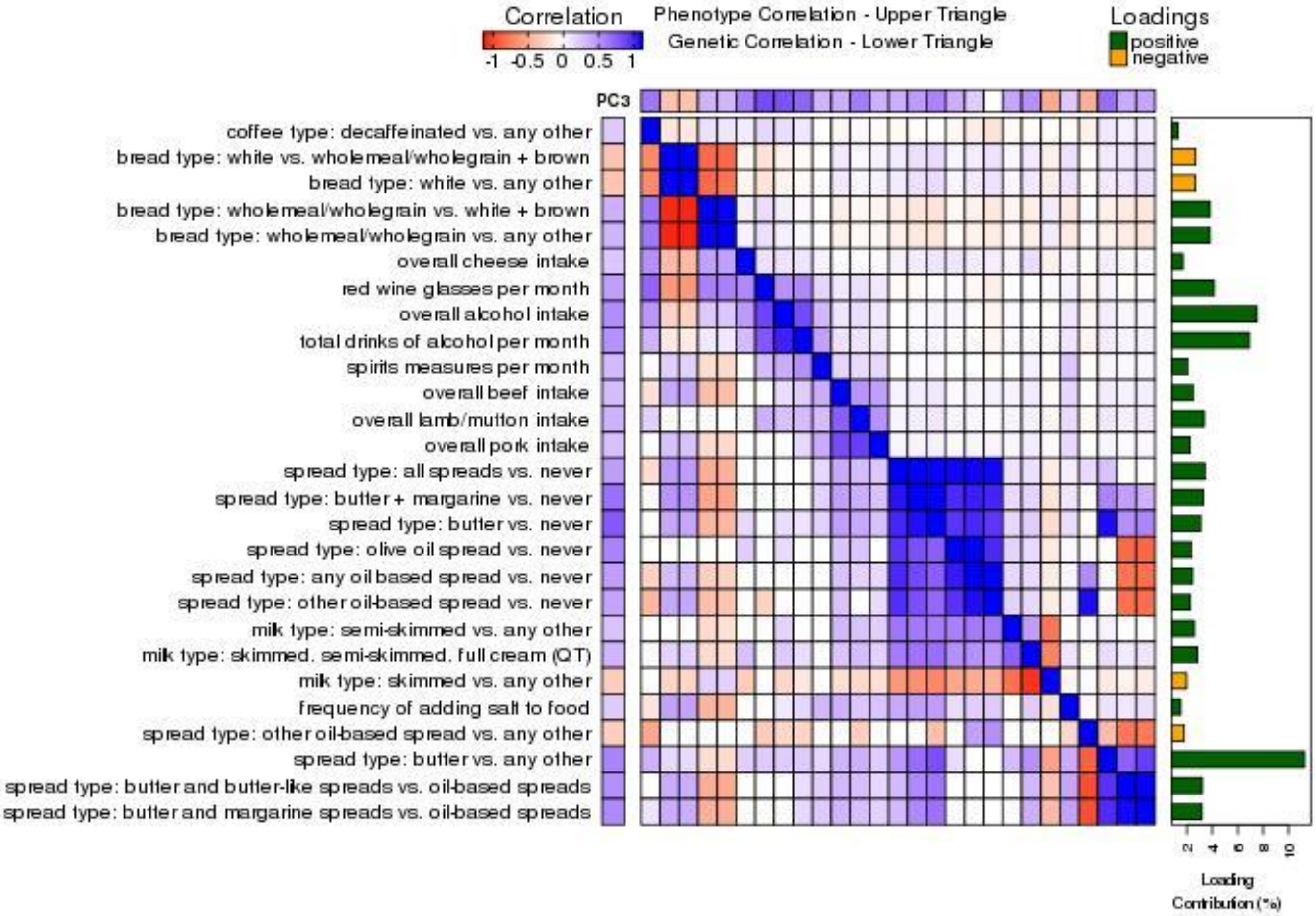
Correlation
-1 -0.5 0 0.5 1
Phenotype Correlation - Upper Triangle
Genetic Correlation - Lower Triangle
Loadings
positive
negative
PC3
coffee type: decaffeinated vs. any other
bread type: white vs. wholemeal/wholegrain + brown
bread type: white vs. any other
bread type: wholemeal/wholegrain vs. white + brown
bread type: wholemeal/wholegrain vs. any other
overall cheese intake
red wine glasses per month
overall alcohol intake
total drinks of alcohol per month
spirits measures per month
overall beef intake
overall lamb/mutton intake
overall pork intake
spread type: all spreads vs. never
spread type: butter + margarine vs. never
spread type: butter vs. never
spread type: olive oil spread vs. never
spread type: any oil based spread vs. never
spread type: other oil-based spread vs. never
milk type: semi-skimmed vs. any other
milk type: skimmed, semi-skimmed, full cream (QT)
milk type: skimmed vs. any other
frequency of adding salt to food
spread type: other oil-based spread vs. any other
spread type: butter vs. any other
spread type: butter and butter-like spreads vs. oil-based spreads
spread type: butter and margarine spreads vs. oil-based spreads
2 4 6 8 10
Loading Contribution (%)

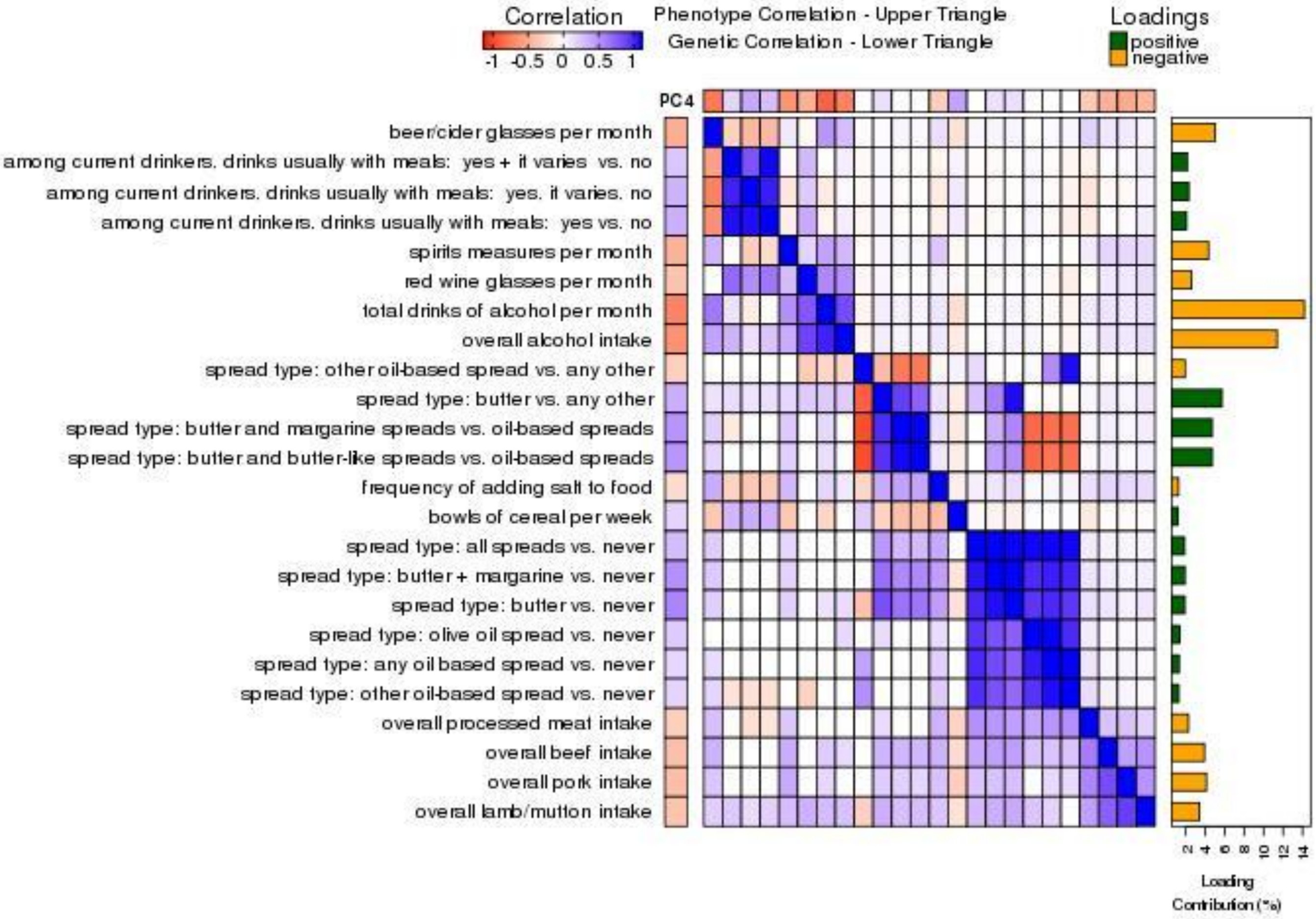
Correlation
-1 -0.5 0 0.5 1
Phenotype Correlation - Upper Triangle
Genetic Correlation - Lower Triangle
Loadings
positive
negative
PC4
beer/cider glasses per month
among current drinkers, drinks usually with meals: yes + it varies vs. no
among current drinkers, drinks usually with meals: yes, it varies, no
among current drinkers, drinks usually with meals: yes vs. no
spirits measures per month
red wine glasses per month
total drinks of alcohol per month
overall alcohol intake
spread type: other oil-based spread vs. any other
spread type: butter vs. any other
spread type: butter and margarine spreads vs. oil-based spreads
spread type: butter and butter-like spreads vs. oil-based spreads
frequency of adding salt to food
bowls of cereal per week
spread type: all spreads vs. never
spread type: butter + margarine vs. never
spread type: butter vs. never
spread type: olive oil spread vs. never
spread type: any oil based spread vs. never
spread type: other oil-based spread vs. never
overall processed meat intake
overall beef intake
overall pork intake
overall lamb/mutton intake
2 4 6 8 10 12 14
Loading Contribution (%)

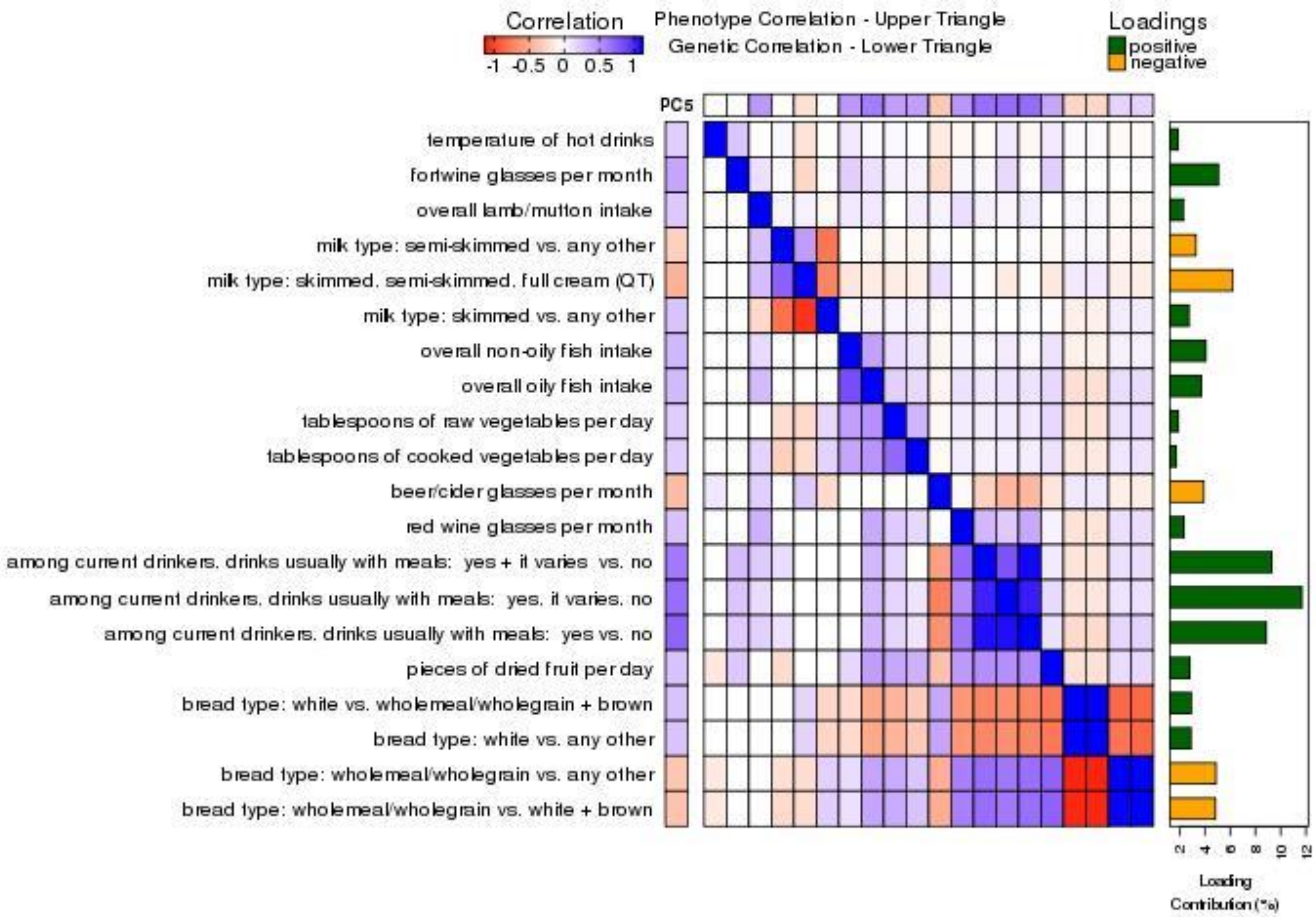

Correlation
-1 -0.5 0 0.5 1
Phenotype Correlation - Upper Triangle
Genetic Correlation - Lower Triangle
Loadings
positive
negative
PC5
temperature of hot drinks
fortwine glasses per month
overall lamb/mutton intake
milk type: semi-skimmed vs. any other
milk type: skimmed, semi-skimmed, full cream (QT)
milk type: skimmed vs. any other
overall non-oily fish intake
overall oily fish intake
tablespoons of raw vegetables per day
tablespoons of cooked vegetables per day
beer/cider glasses per month
red wine glasses per month
among current drinkers, drinks usually with meals: yes + it varies vs. no
among current drinkers, drinks usually with meals: yes, it varies, no
among current drinkers, drinks usually with meals: yes vs. no
pieces of dried fruit per day
bread type: white vs. wholemeal/wholegrain + brown
bread type: white vs. any other
bread type: wholemeal/wholegrain vs. any other
bread type: wholemeal/wholegrain vs. white + brown
2 4 6 8 10 12
Loading
Contribution (%)

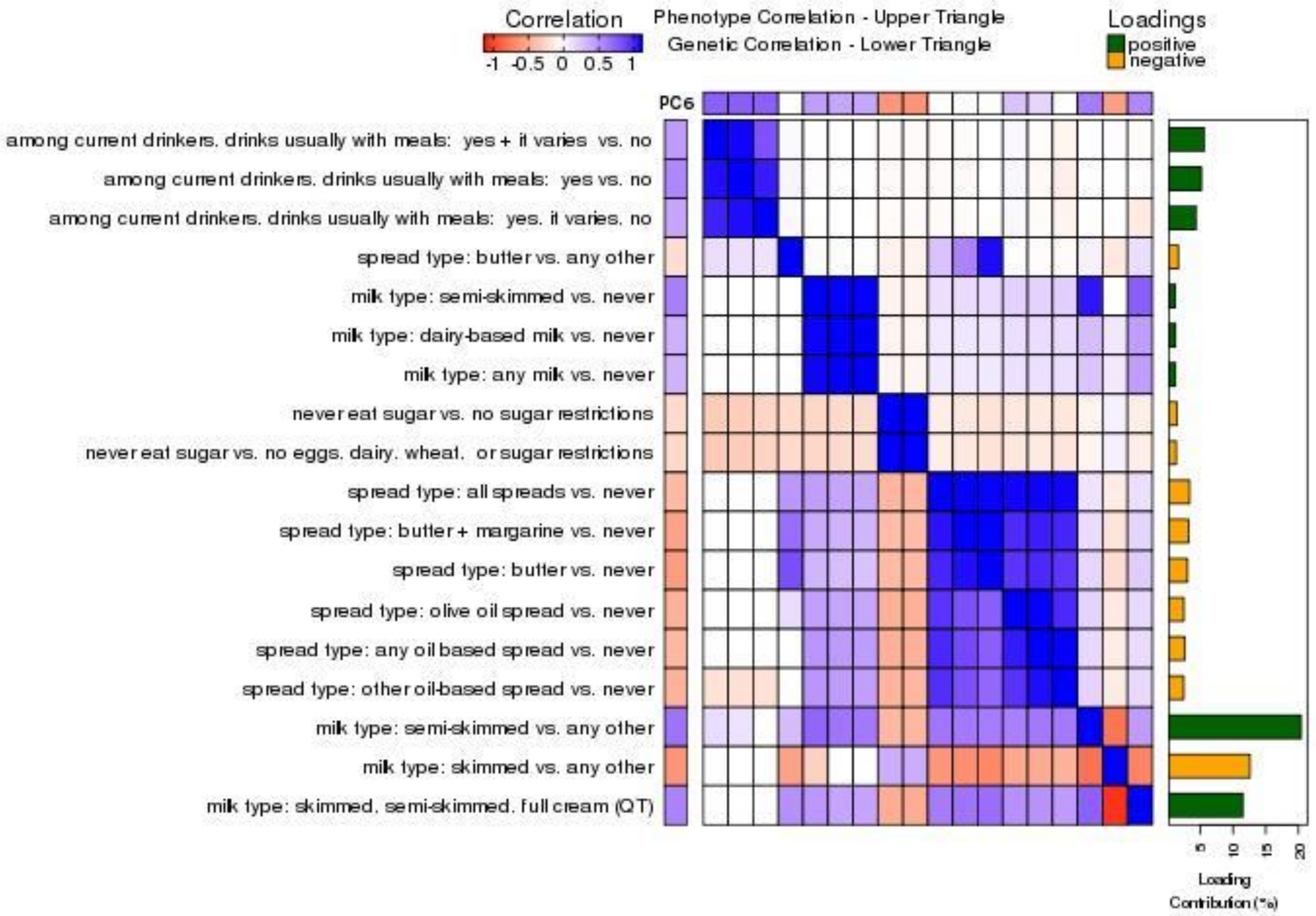

Correlation
-1 -0.5 0 0.5 1
Phenotype Correlation - Upper Triangle
Genetic Correlation - Lower Triangle
Loadings
positive
negative
PC6
among current drinkers, drinks usually with meals: yes + it varies vs. no
among current drinkers, drinks usually with meals: yes vs. no
among current drinkers, drinks usually with meals: yes, it varies, no
spread type: butter vs. any other
milk type: semi-skimmed vs. never
milk type: dairy-based milk vs. never
milk type: any milk vs. never
never eat sugar vs. no sugar restrictions
never eat sugar vs. no eggs, dairy, wheat, or sugar restrictions
spread type: all spreads vs. never
spread type: butter + margarine vs. never
spread type: butter vs. never
spread type: olive oil spread vs. never
spread type: any oil based spread vs. never
spread type: other oil-based spread vs. never
milk type: semi-skimmed vs. any other
milk type: skimmed vs. any other
milk type: skimmed, semi-skimmed, full cream (QT)
5 10 15 20
Loading
Contribution (%)

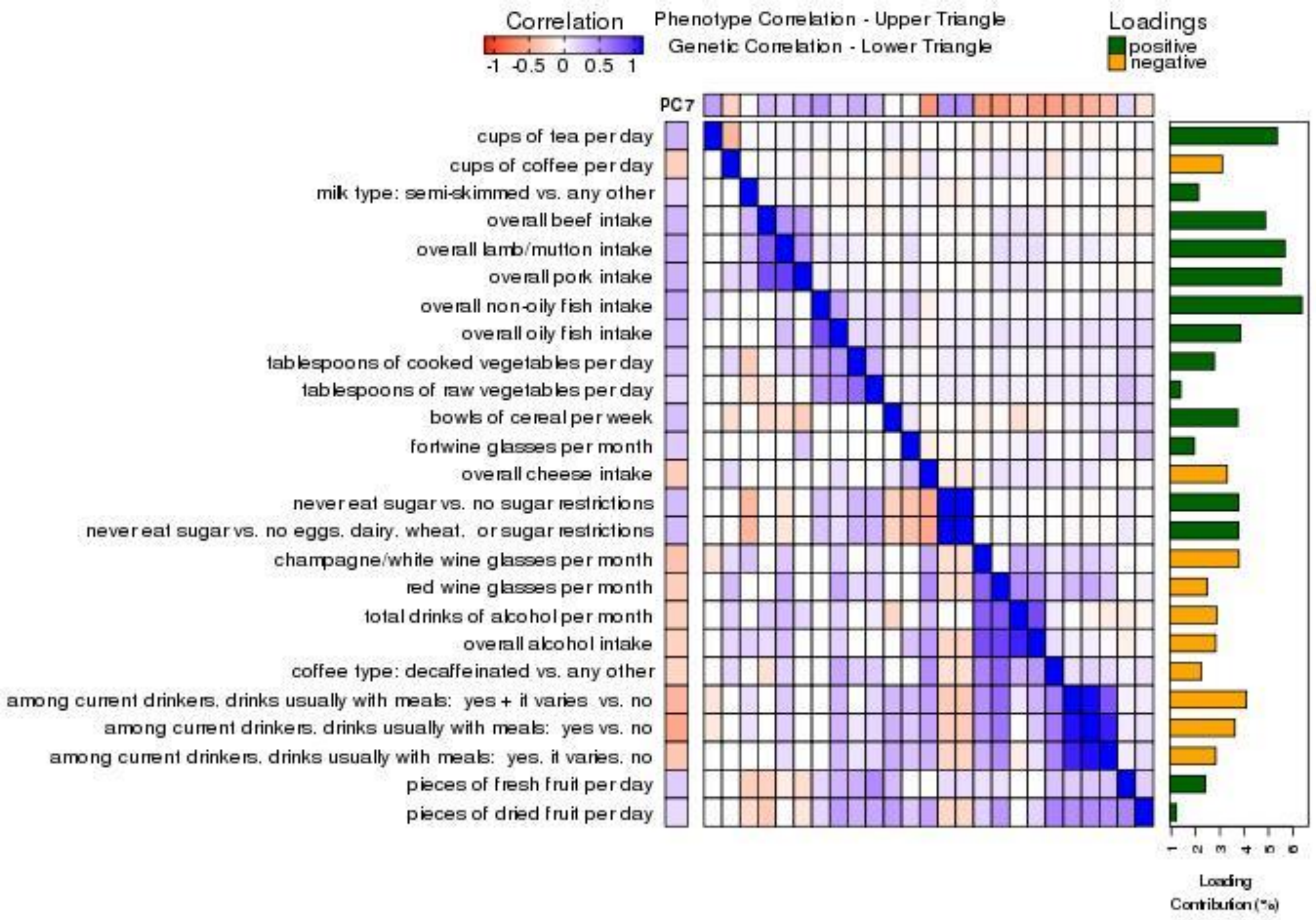
Correlation
-1 -0.5 0 0.5 1
Phenotype Correlation - Upper Triangle
Genetic Correlation - Lower Triangle
Loadings
positive
negative
PC7
cups of tea per day
cups of coffee per day
milk type: semi-skimmed vs. any other
overall beef intake
overall lamb/mutton intake
overall pork intake
overall non-oily fish intake
overall oily fish intake
tablespoons of cooked vegetables per day
tablespoons of raw vegetables per day
bowls of cereal per week
fortwine glasses per month
overall cheese intake
never eat sugar vs. no sugar restrictions
never eat sugar vs. no eggs, dairy, wheat, or sugar restrictions
champagne/white wine glasses per month
red wine glasses per month
total drinks of alcohol per month
overall alcohol intake
coffee type: decaffeinated vs. any other
among current drinkers, drinks usually with meals: yes + it varies vs. no
among current drinkers, drinks usually with meals: yes vs. no
among current drinkers, drinks usually with meals: yes, it varies, no
pieces of fresh fruit per day
pieces of dried fruit per day
1 2 3 4 5 6
Loading Contribution (%)

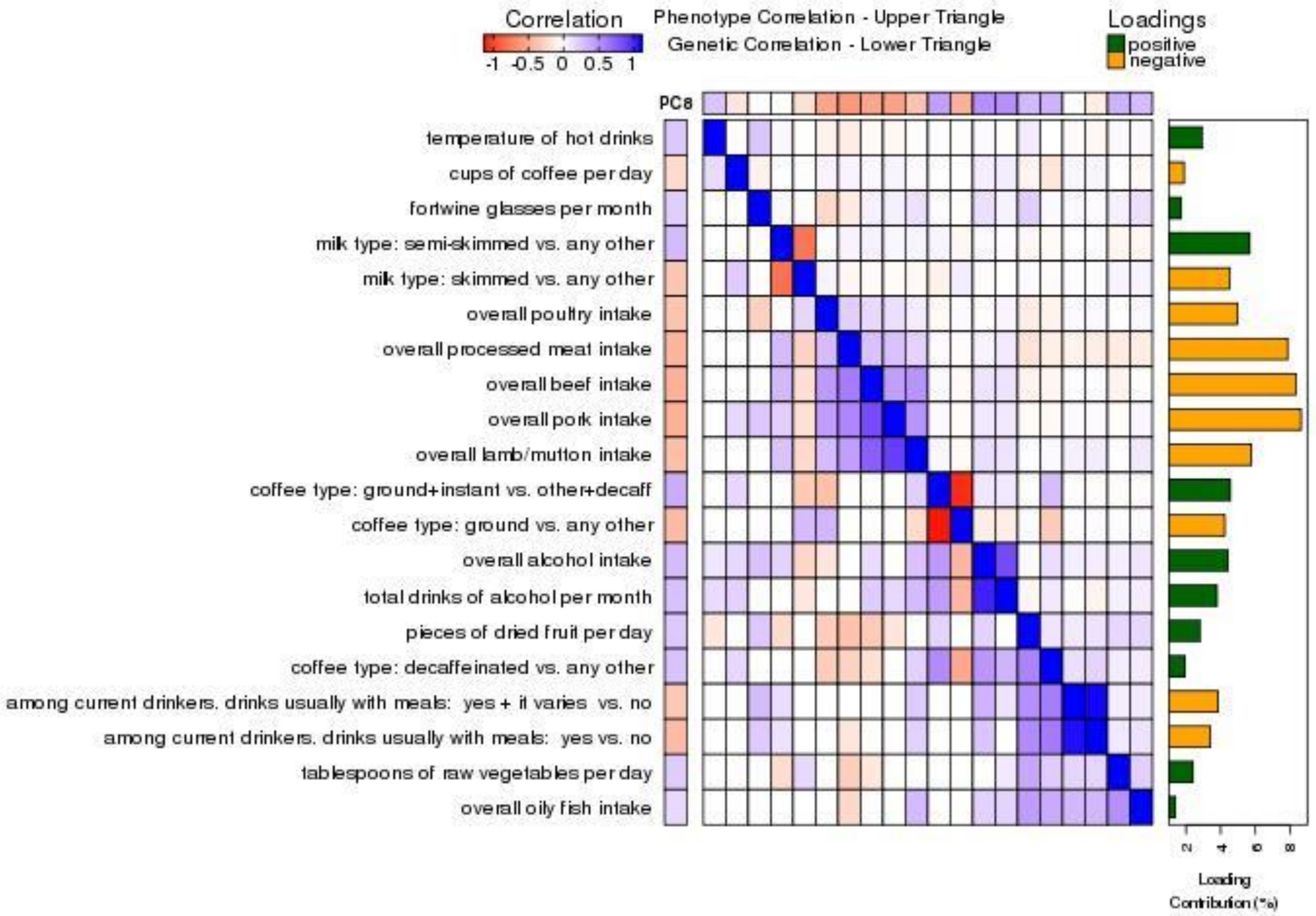
Correlation
-1 -0.5 0 0.5 1
Phenotype Correlation - Upper Triangle
Genetic Correlation - Lower Triangle
Loadings
positive
negative
PC8
temperature of hot drinks
cups of coffee per day
fortwine glasses per month
milk type: semi-skimmed vs. any other
milk type: skimmed vs. any other
overall poultry intake
overall processed meat intake
overall beef intake
overall pork intake
overall lamb/mutton intake
coffee type: ground+instant vs. other+decaff
coffee type: ground vs. any other
overall alcohol intake
total drinks of alcohol per month
pieces of dried fruit per day
coffee type: decaffeinated vs. any other
among current drinkers, drinks usually with meals: yes + it varies vs. no
among current drinkers, drinks usually with meals: yes vs. no
tablespoons of raw vegetables per day
overall oily fish intake
2 4 6 8
Loading Contribution (%)

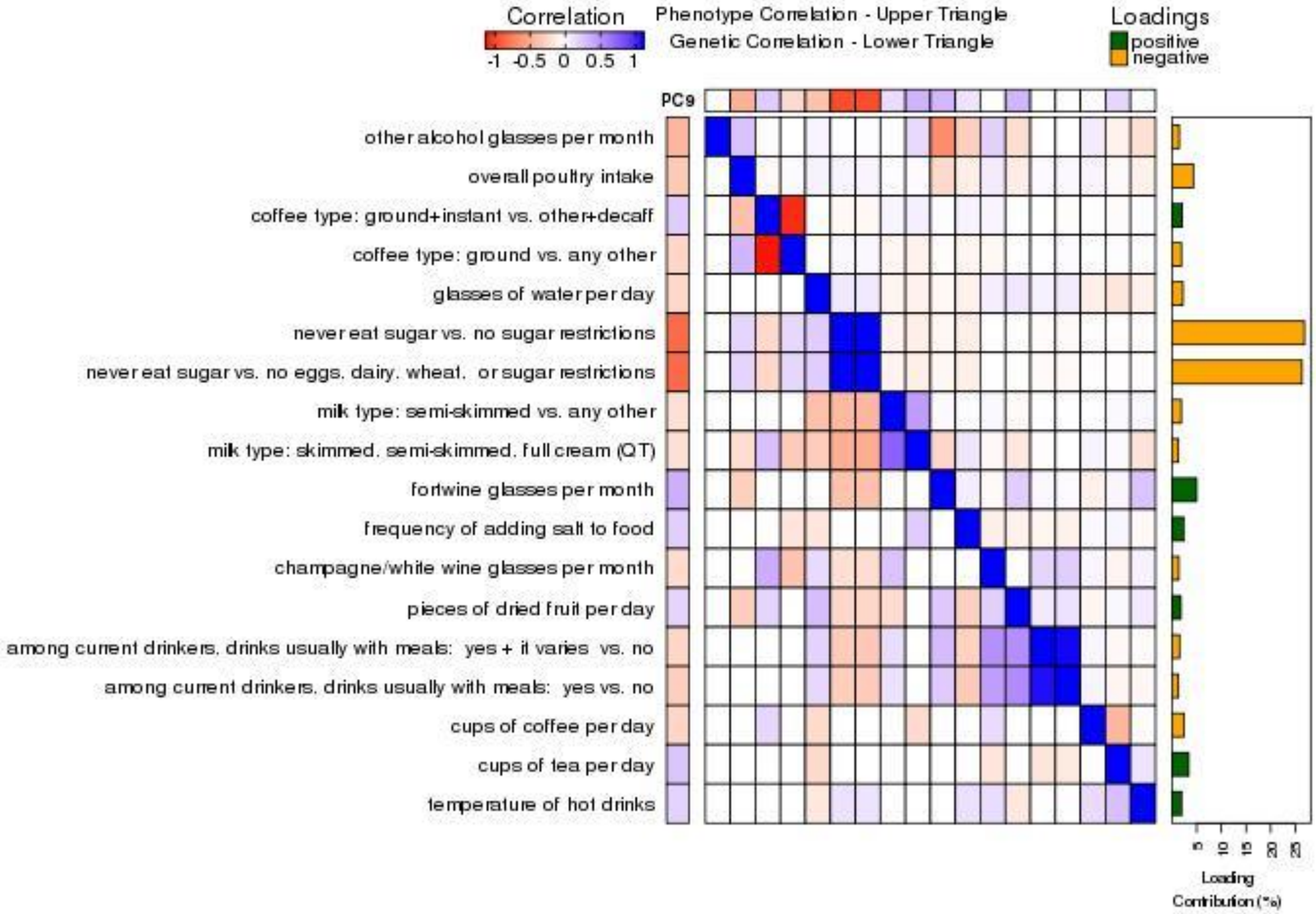
Correlation
-1 -0.5 0 0.5 1
Phenotype Correlation - Upper Triangle
Genetic Correlation - Lower Triangle
Loadings
positive
negative
PC9
other alcohol glasses per month
overall poultry intake
coffee type: ground+instant vs. other+decaff
coffee type: ground vs. any other
glasses of water per day
never eat sugar vs. no sugar restrictions
never eat sugar vs. no eggs, dairy, wheat, or sugar restrictions
milk type: semi-skimmed vs. any other
milk type: skimmed, semi-skimmed, full cream (QT)
fortwine glasses per month
frequency of adding salt to food
champagne/white wine glasses per month
pieces of dried fruit per day
among current drinkers, drinks usually with meals: yes + it varies vs. no
among current drinkers, drinks usually with meals: yes vs. no
cups of coffee per day
cups of tea per day
temperature of hot drinks
5 10 15 20 25
Loading
Contribution (%)

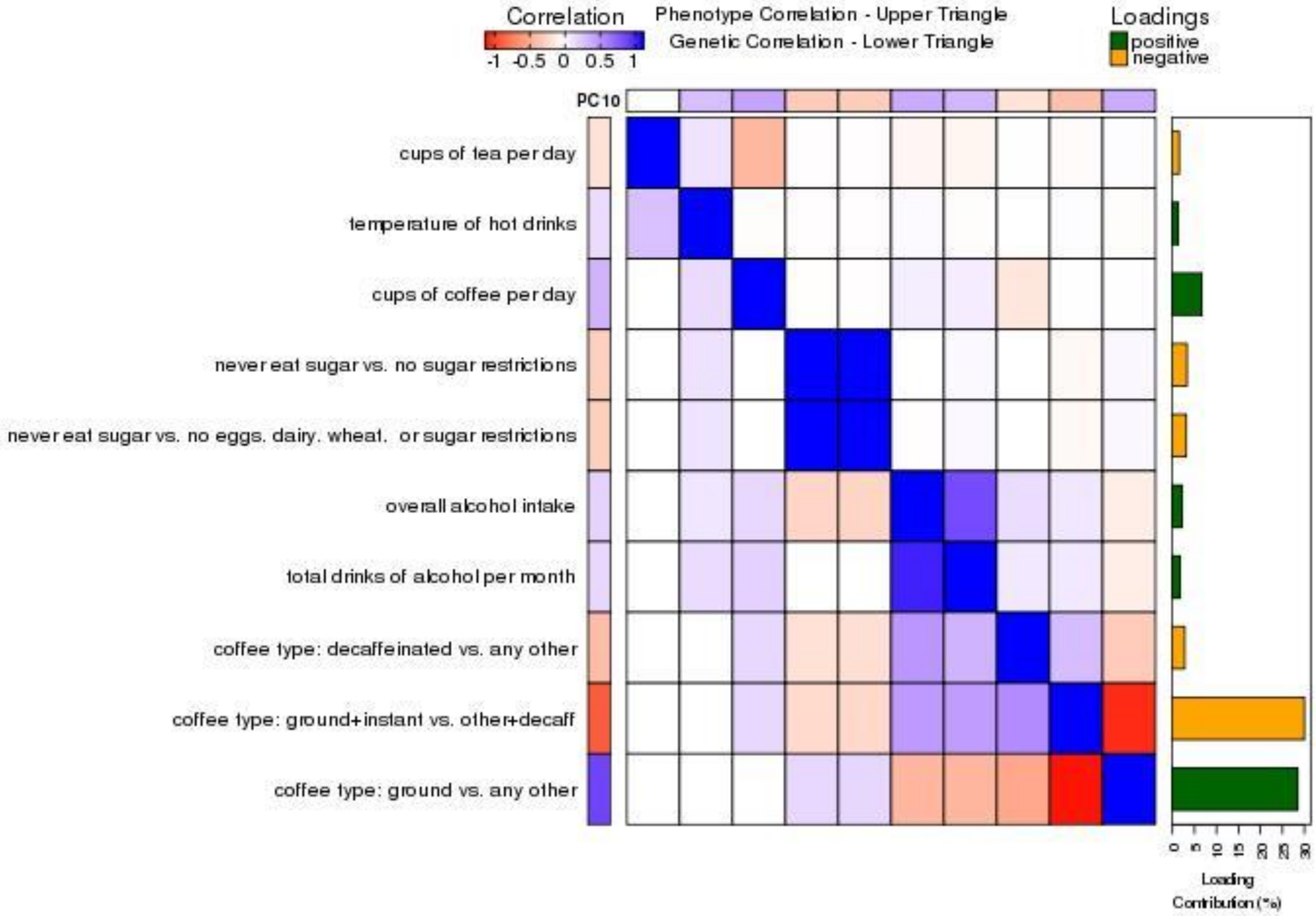
Correlation
-1 -0.5 0 0.5 1
Phenotype Correlation - Upper Triangle
Genetic Correlation - Lower Triangle
Loadings
positive
negative
PC10
cups of tea per day
temperature of hot drinks
cups of coffee per day
never eat sugar vs. no sugar restrictions
never eat sugar vs. no eggs, dairy, wheat, or sugar restrictions
overall alcohol intake
total drinks of alcohol per month
coffee type: decaffeinated vs. any other
coffee type: ground+instant vs. other+decaff
coffee type: ground vs. any other
0 5 10 15 20 25 30
Loading
Contribution (%)

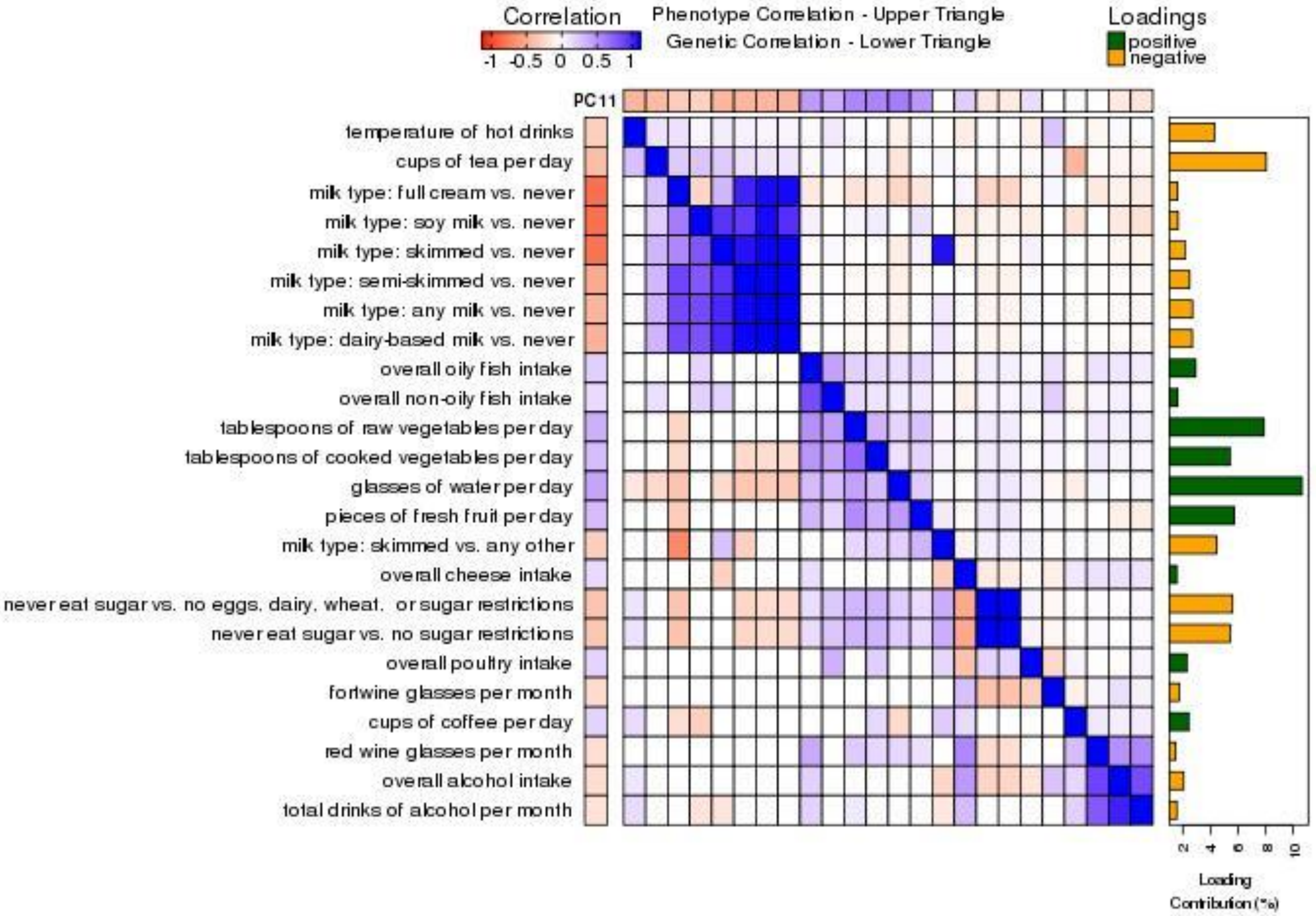
Correlation
-1 -0.5 0 0.5 1
Phenotype Correlation - Upper Triangle
Genetic Correlation - Lower Triangle
Loadings
positive
negative
PC11
temperature of hot drinks
cups of tea per day
milk type: full cream vs. never
milk type: soy milk vs. never
milk type: skimmed vs. never
milk type: semi-skimmed vs. never
milk type: any milk vs. never
milk type: dairy-based milk vs. never
overall oily fish intake
overall non-oily fish intake
tablespoons of raw vegetables per day
tablespoons of cooked vegetables per day
glasses of water per day
pieces of fresh fruit per day
milk type: skimmed vs. any other
overall cheese intake
never eat sugar vs. no eggs, dairy, wheat, or sugar restrictions
never eat sugar vs. no sugar restrictions
overall poultry intake
fortwine glasses per month
cups of coffee per day
red wine glasses per month
overall alcohol intake
total drinks of alcohol per month
2 4 6 8 10
Loading
Contribution (%)

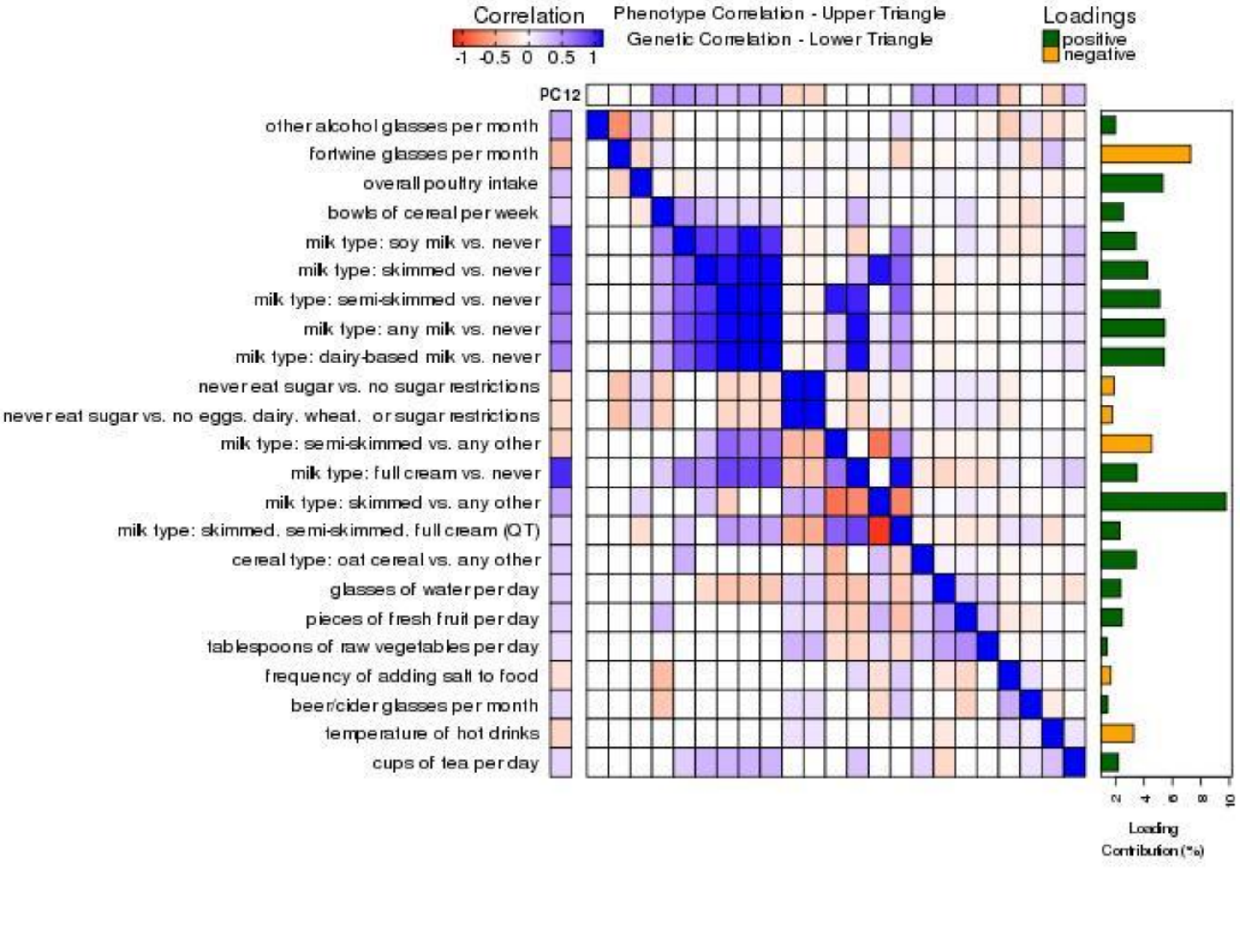
Correlation
-1 -0.5 0 0.5 1
Phenotype Correlation - Upper Triangle
Genetic Correlation - Lower Triangle
Loadings
positive
negative
PC12
other alcohol glasses per month
fortwine glasses per month
overall poultry intake
bowls of cereal per week
milk type: soy milk vs. never
milk type: skimmed vs. never
milk type: semi-skimmed vs. never
milk type: any milk vs. never
milk type: dairy-based milk vs. never
never eat sugar vs. no sugar restrictions
never eat sugar vs. no eggs, dairy, wheat, or sugar restrictions
milk type: semi-skimmed vs. any other
milk type: full cream vs. never
milk type: skimmed vs. any other
milk type: skimmed, semi-skimmed, full cream (QT)
cereal type: oat cereal vs. any other
glasses of water per day
pieces of fresh fruit per day
tablespoons of raw vegetables per day
frequency of adding salt to food
beer/cider glasses per month
temperature of hot drinks
cups of tea per day
2 4 6 8 10
Loading
Contribution (%)

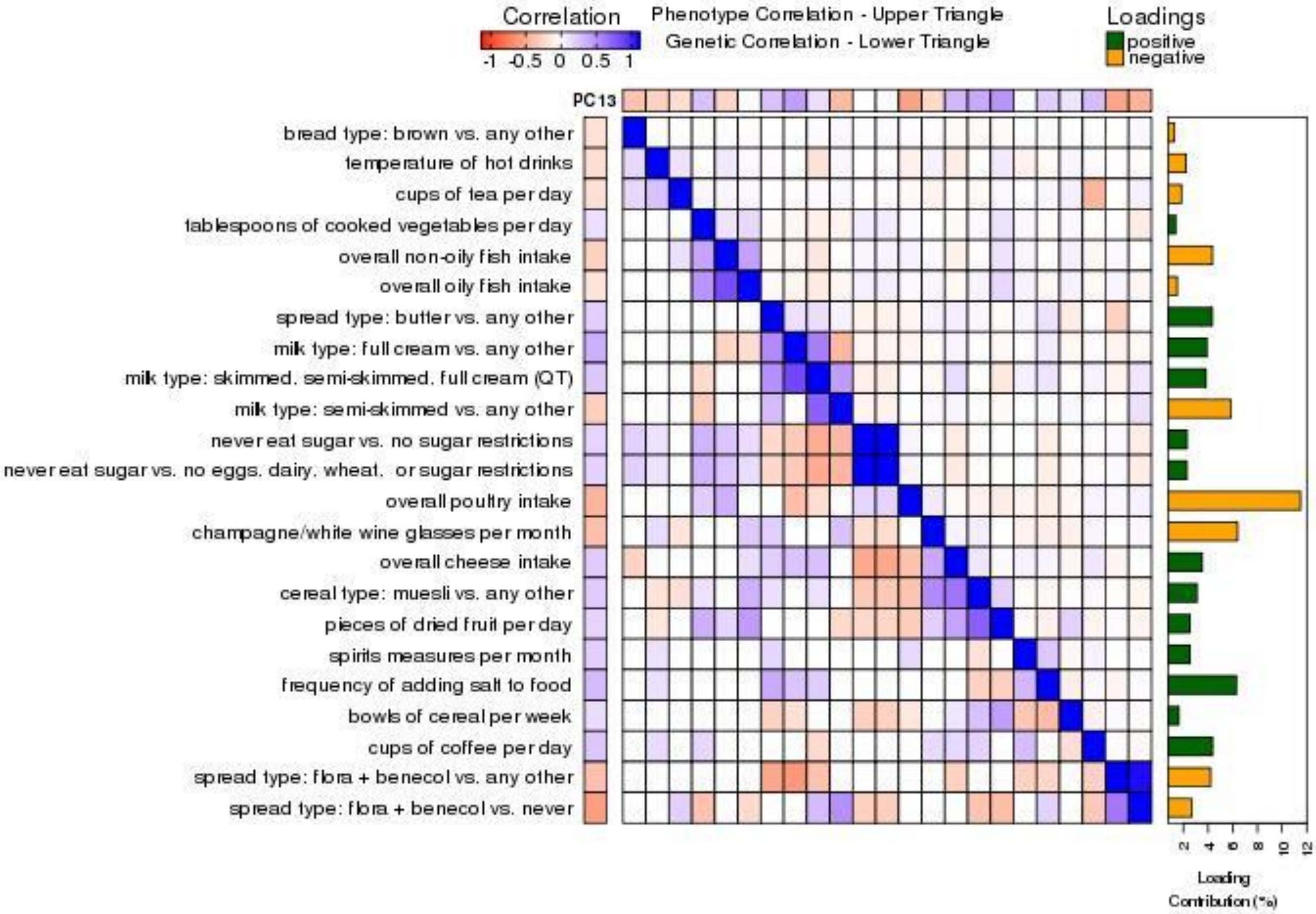
Correlation
-1 -0.5 0 0.5 1
Phenotype Correlation - Upper Triangle
Genetic Correlation - Lower Triangle
Loadings
positive
negative
PC13
bread type: brown vs. any other
temperature of hot drinks
cups of tea per day
tablespoons of cooked vegetables per day
overall non-oily fish intake
overall oily fish intake
spread type: butter vs. any other
milk type: full cream vs. any other
milk type: skimmed, semi-skimmed, full cream (QT)
milk type: semi-skimmed vs. any other
never eat sugar vs. no sugar restrictions
never eat sugar vs. no eggs, dairy, wheat, or sugar restrictions
overall poultry intake
champagne/white wine glasses per month
overall cheese intake
cereal type: muesli vs. any other
pieces of dried fruit per day
spirits measures per month
frequency of adding salt to food
bowls of cereal per week
cups of coffee per day
spread type: flora + benecol vs. any other
spread type: flora + benecol vs. never
2 4 6 8 10 12
Loading
Contribution (%)

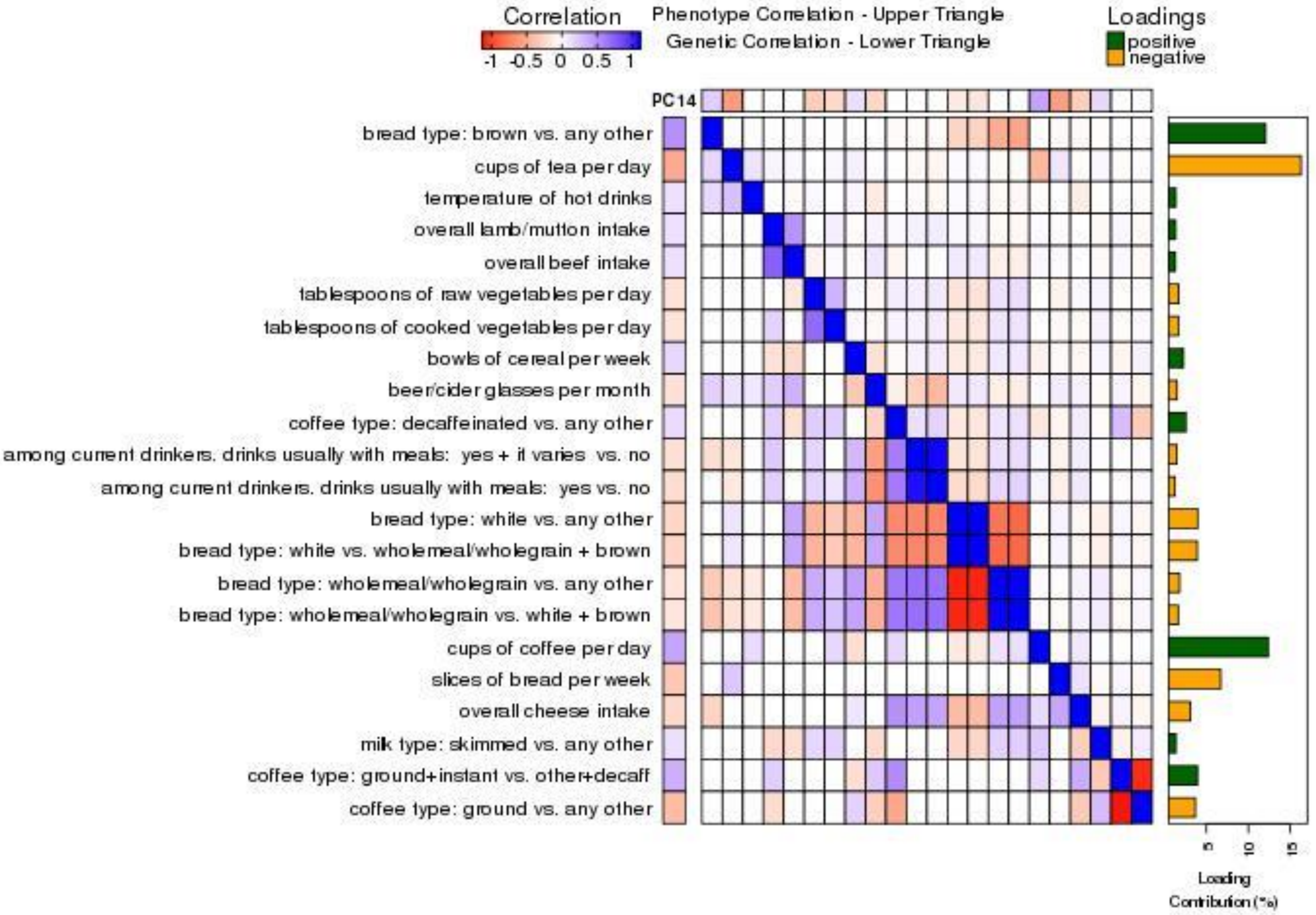
Correlation
-1 -0.5 0 0.5 1
Phenotype Correlation - Upper Triangle
Genetic Correlation - Lower Triangle
Loadings
positive
negative
PC14
bread type: brown vs. any other
cups of tea per day
temperature of hot drinks
overall lamb/mutton intake
overall beef intake
tablespoons of raw vegetables per day
tablespoons of cooked vegetables per day
bowls of cereal per week
beer/cider glasses per month
coffee type: decaffeinated vs. any other
among current drinkers, drinks usually with meals: yes + it varies vs. no
among current drinkers, drinks usually with meals: yes vs. no
bread type: white vs. any other
bread type: white vs. wholemeal/wholegrain + brown
bread type: wholemeal/wholegrain vs. any other
bread type: wholemeal/wholegrain vs. white + brown
cups of coffee per day
slices of bread per week
overall cheese intake
milk type: skimmed vs. any other
coffee type: ground+instant vs. other+decaff
coffee type: ground vs. any other
5 10 15
Loading
Contribution (%)

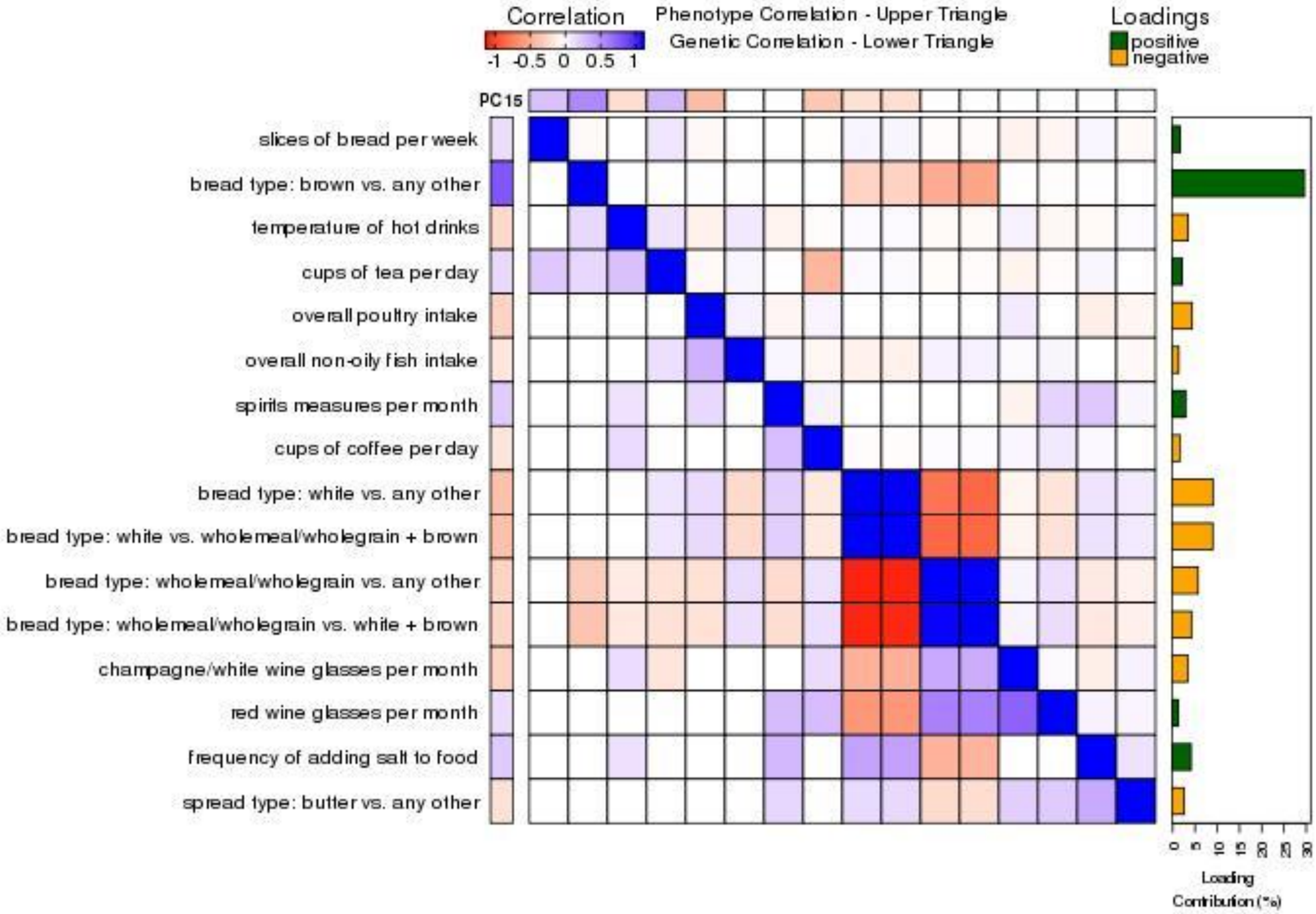
Correlation
-1 -0.5 0 0.5 1
Phenotype Correlation - Upper Triangle
Genetic Correlation - Lower Triangle
Loadings
positive
negative
PC15
slices of bread per week
bread type: brown vs. any other
temperature of hot drinks
cups of tea per day
overall poultry intake
overall non-oily fish intake
spirits measures per month
cups of coffee per day
bread type: white vs. any other
bread type: white vs. wholemeal/wholegrain + brown
bread type: wholemeal/wholegrain vs. any other
bread type: wholemeal/wholegrain vs. white + brown
champagne/white wine glasses per month
red wine glasses per month
frequency of adding salt to food
spread type: butter vs. any other
0 5 10 15 20 25 30
Loading
Contribution (%)

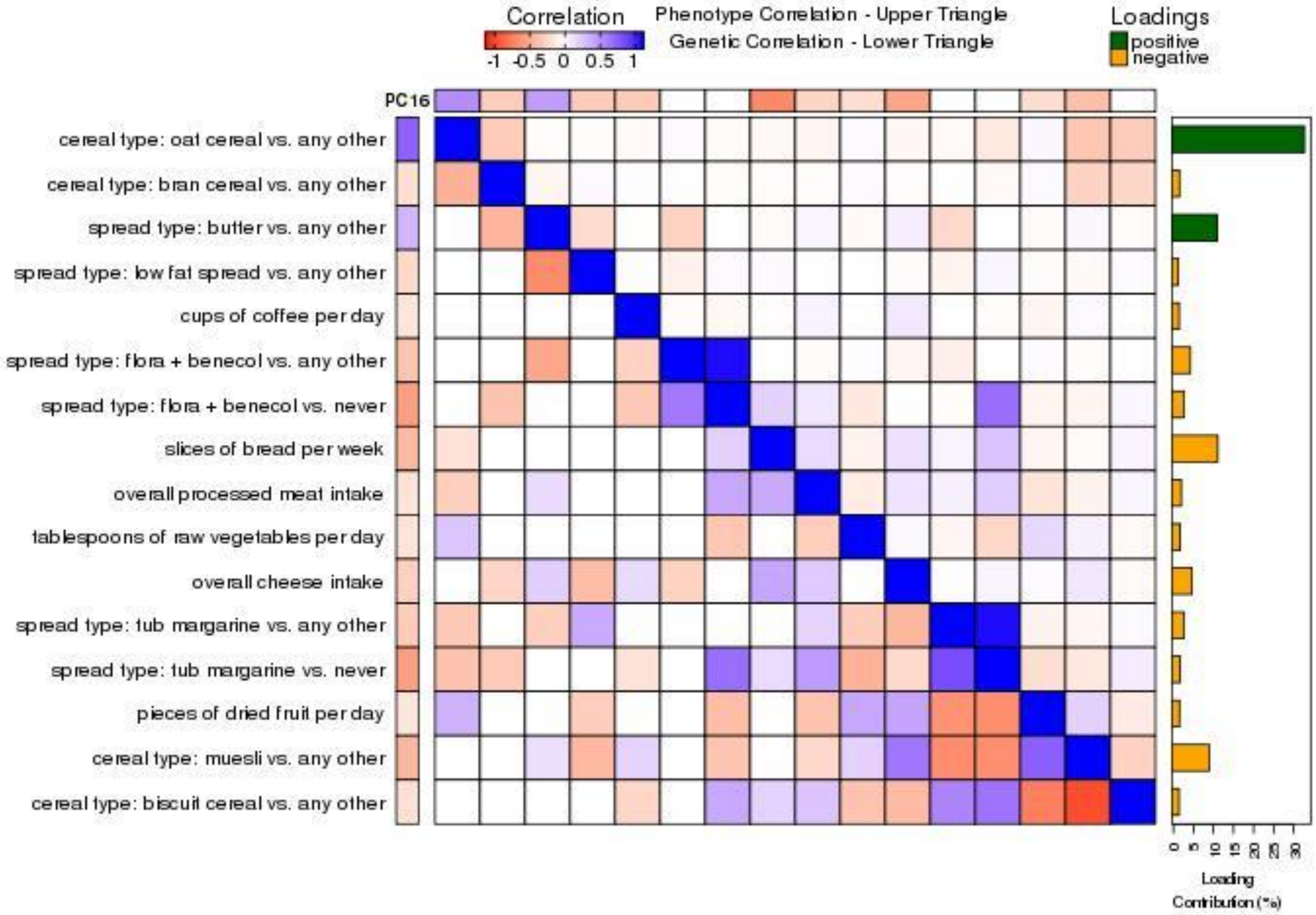
Correlation
-1 -0.5 0 0.5 1
Phenotype Correlation - Upper Triangle
Genetic Correlation - Lower Triangle
Loadings
positive
negative
PC16
cereal type: oat cereal vs. any other
cereal type: bran cereal vs. any other
spread type: butter vs. any other
spread type: low fat spread vs. any other
cups of coffee per day
spread type: flora + benecol vs. any other
spread type: flora + benecol vs. never
slices of bread per week
overall processed meat intake
tablespoons of raw vegetables per day
overall cheese intake
spread type: tub margarine vs. any other
spread type: tub margarine vs. never
pieces of dried fruit per day
cereal type: muesli vs. any other
cereal type: biscuit cereal vs. any other
0 5 10 15 20 25 30
Loading
Contribution (%)

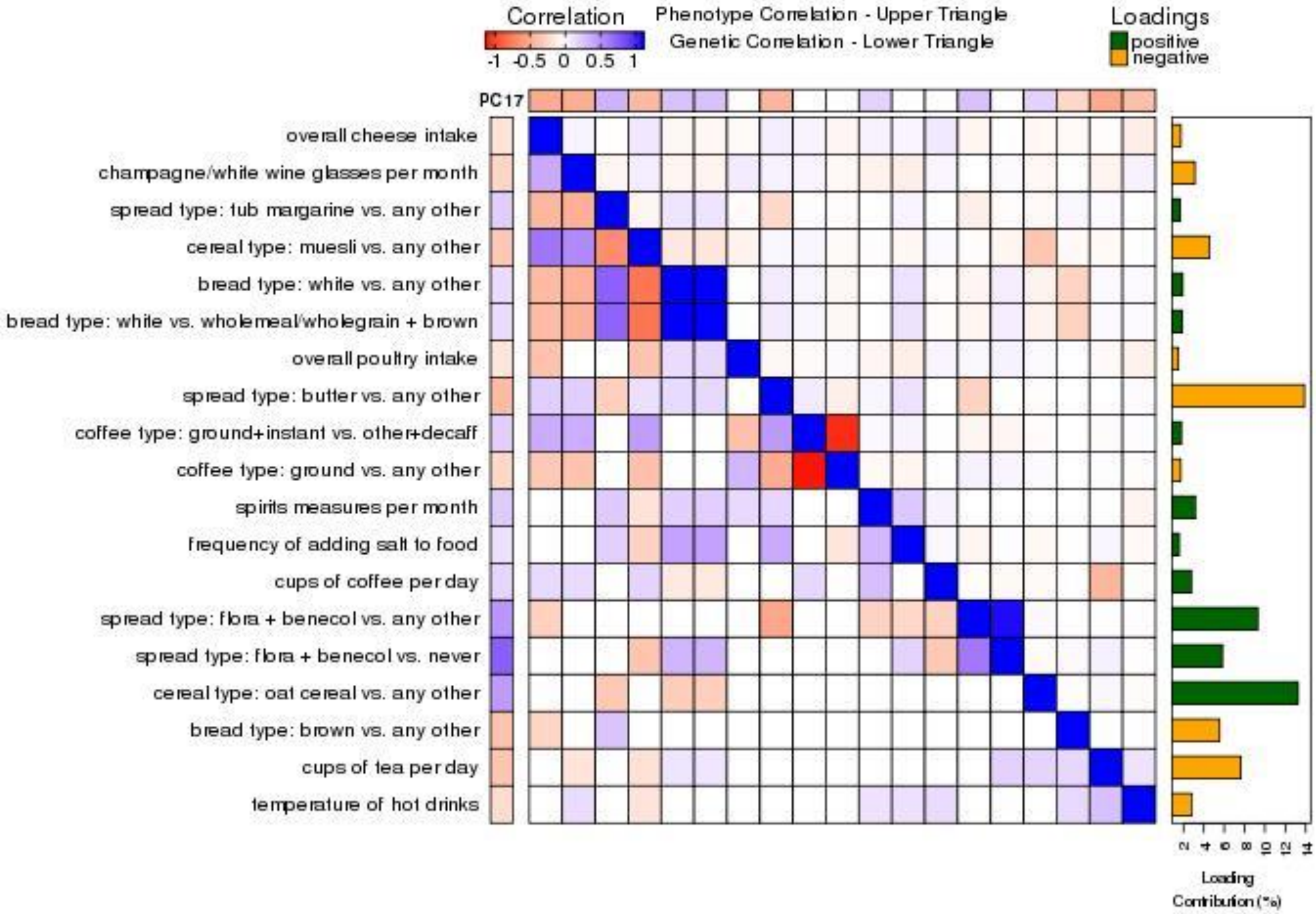
Correlation
-1 -0.5 0 0.5 1
Phenotype Correlation - Upper Triangle
Genetic Correlation - Lower Triangle
Loadings
positive
negative
PC17
overall cheese intake
champagne/white wine glasses per month
spread type: tub margarine vs. any other
cereal type: muesli vs. any other
bread type: white vs. any other
bread type: white vs. wholemeal/wholegrain + brown
overall poultry intake
spread type: butter vs. any other
coffee type: ground+instant vs. other+decaff
coffee type: ground vs. any other
spirits measures per month
frequency of adding salt to food
cups of coffee per day
spread type: flora + benecol vs. any other
spread type: flora + benecol vs. never
cereal type: oat cereal vs. any other
bread type: brown vs. any other
cups of tea per day
temperature of hot drinks
2 4 6 8 10 12 14
Loading Contribution (%)

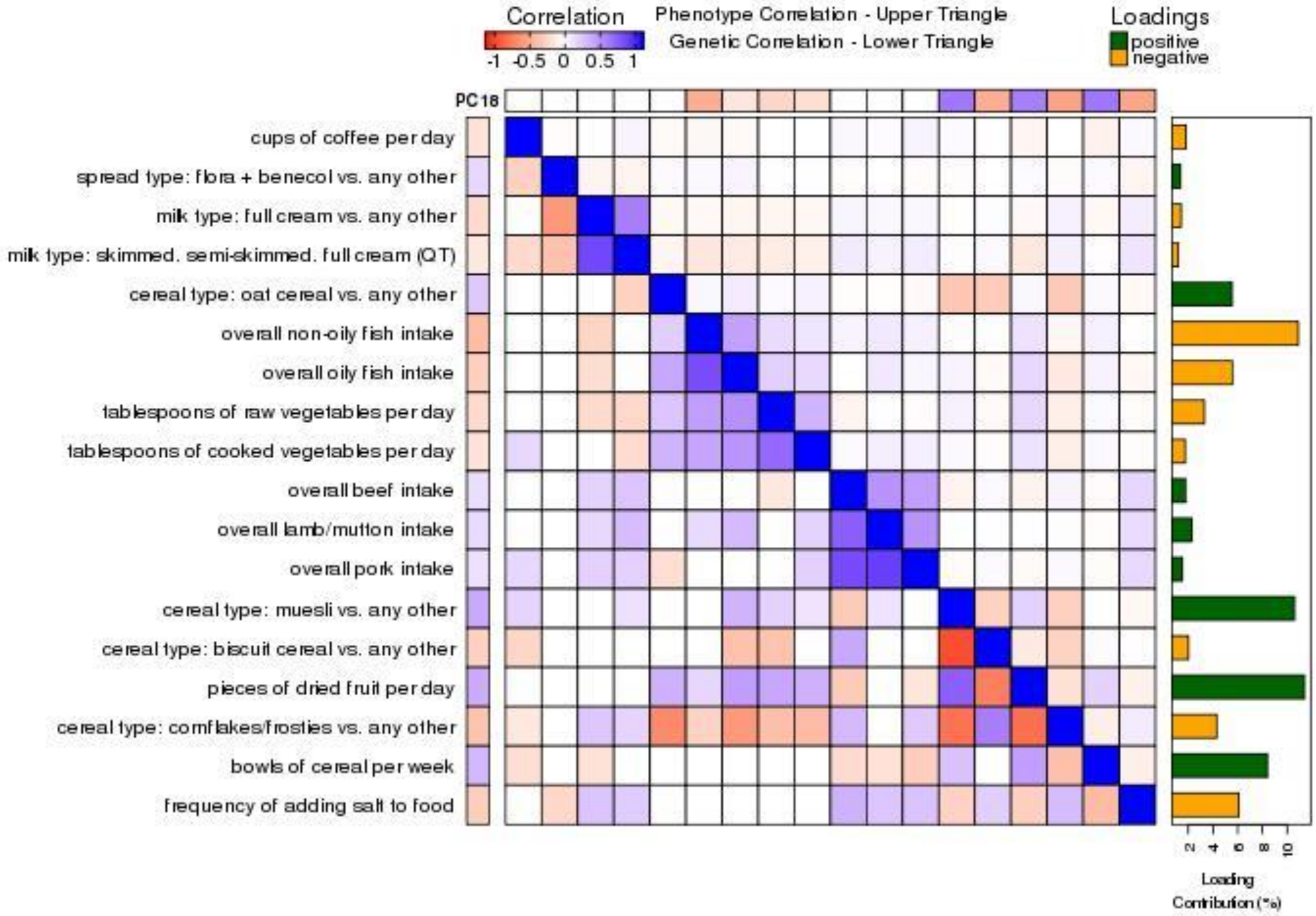
Correlation
-1 -0.5 0 0.5 1
Phenotype Correlation - Upper Triangle
Genetic Correlation - Lower Triangle
Loadings
positive
negative
PC18
cups of coffee per day
spread type: flora + benecol vs. any other
milk type: full cream vs. any other
milk type: skimmed, semi-skimmed, full cream (QT)
cereal type: oat cereal vs. any other
overall non-oily fish intake
overall oily fish intake
tablespoons of raw vegetables per day
tablespoons of cooked vegetables per day
overall beef intake
overall lamb/mutton intake
overall pork intake
cereal type: muesli vs. any other
cereal type: biscuit cereal vs. any other
pieces of dried fruit per day
cereal type: cornflakes/frosties vs. any other
bowls of cereal per week
frequency of adding salt to food
2 4 6 8 10
Loading Contribution (%)

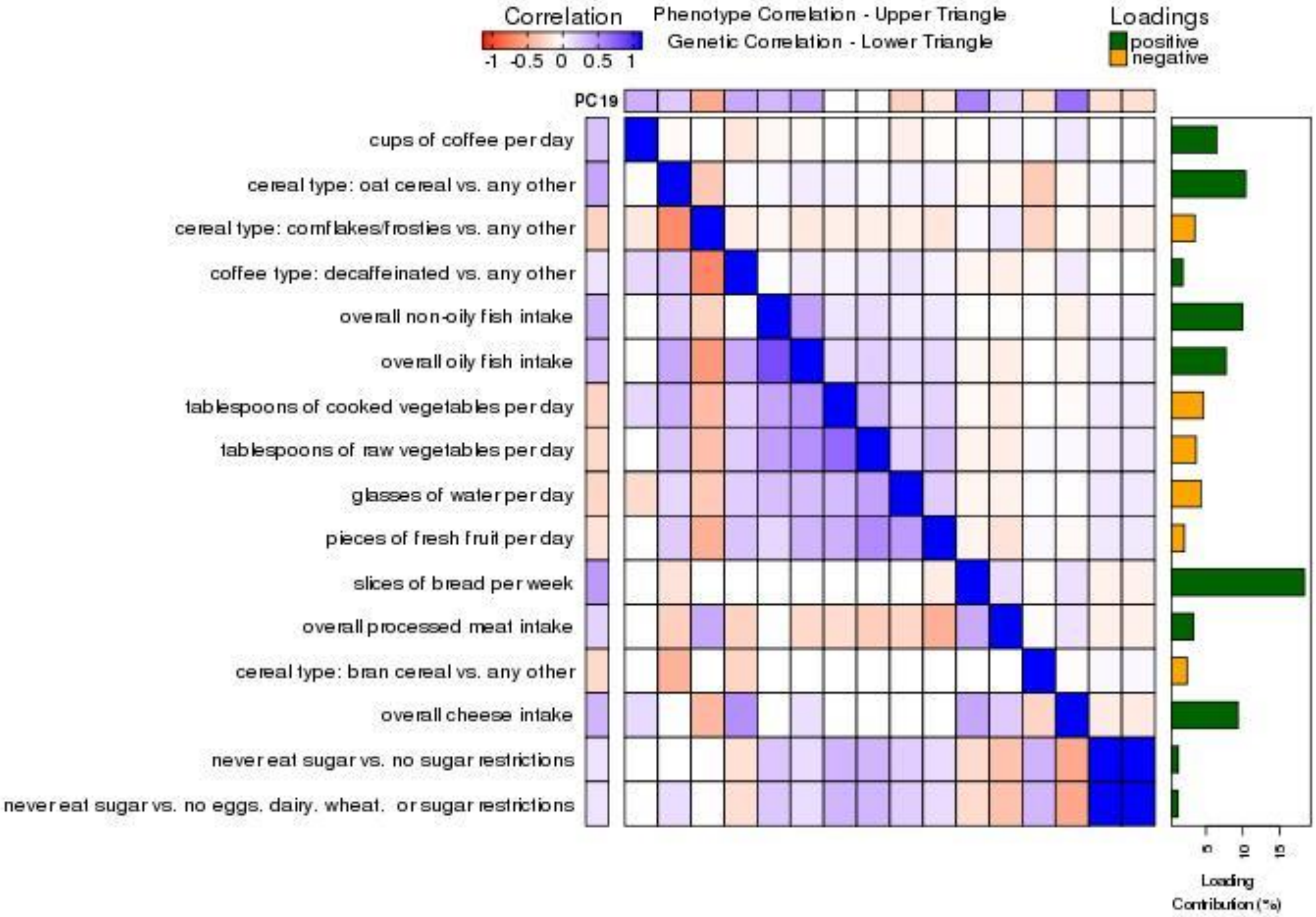
Correlation
-1 -0.5 0 0.5 1
Phenotype Correlation - Upper Triangle
Genetic Correlation - Lower Triangle
Loadings
positive
negative
PC19
cups of coffee per day
cereal type: oat cereal vs. any other
cereal type: cornflakes/frosties vs. any other
coffee type: decaffeinated vs. any other
overall non-oily fish intake
overall oily fish intake
tablespoons of cooked vegetables per day
tablespoons of raw vegetables per day
glasses of water per day
pieces of fresh fruit per day
slices of bread per week
overall processed meat intake
cereal type: bran cereal vs. any other
overall cheese intake
never eat sugar vs. no sugar restrictions
never eat sugar vs. no eggs, dairy, wheat, or sugar restrictions
5 10 15
Loading
Contribution (%)

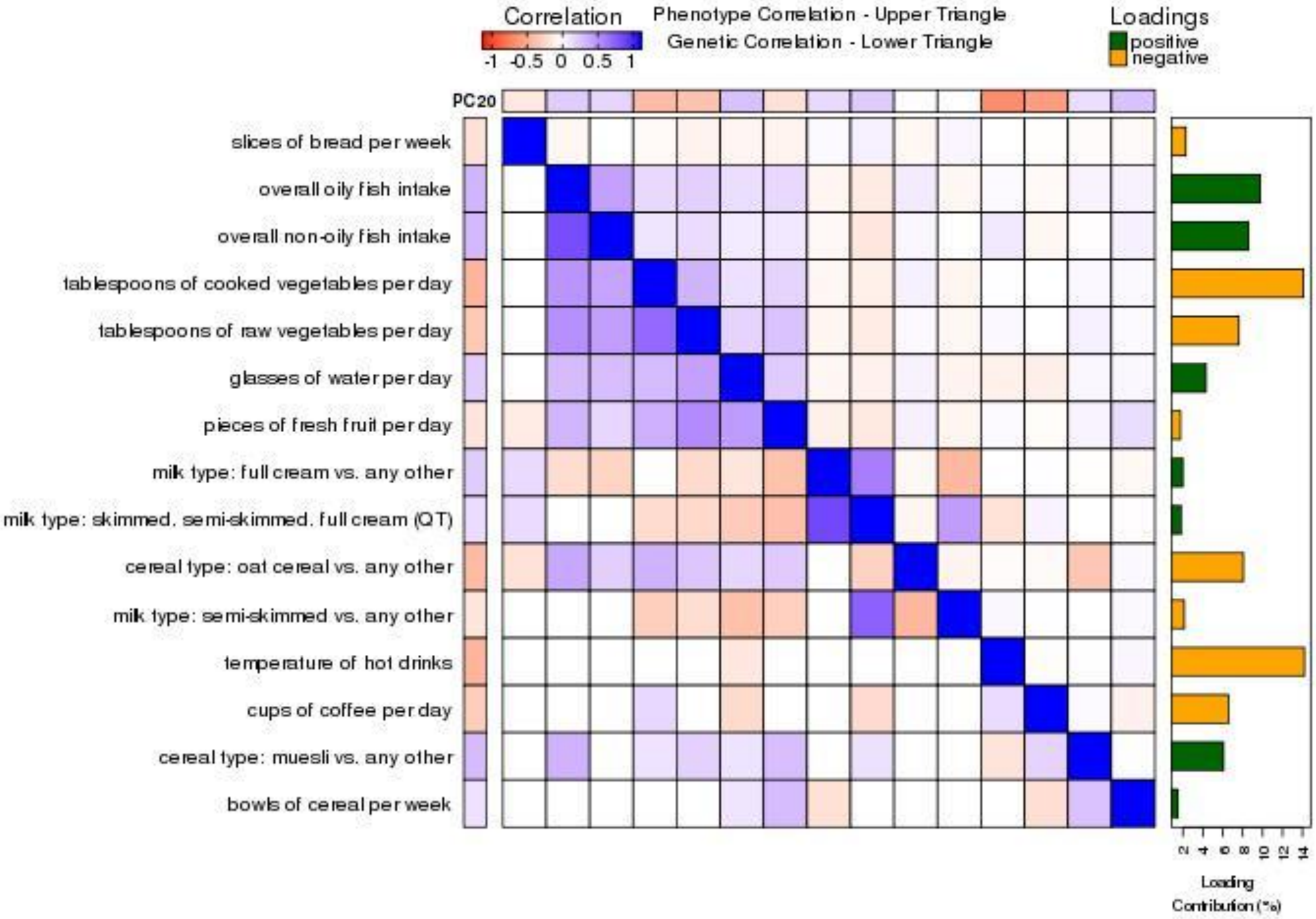
Correlation
-1 -0.5 0 0.5 1
Phenotype Correlation - Upper Triangle
Genetic Correlation - Lower Triangle
Loadings
positive
negative
PC20
slices of bread per week
overall oily fish intake
overall non-oily fish intake
tablespoons of cooked vegetables per day
tablespoons of raw vegetables per day
glasses of water per day
pieces of fresh fruit per day
milk type: full cream vs. any other
milk type: skimmed, semi-skimmed, full cream (QT)
cereal type: oat cereal vs. any other
milk type: semi-skimmed vs. any other
temperature of hot drinks
cups of coffee per day
cereal type: muesli vs. any other
bowls of cereal per week
2 4 6 8 10 12 14
Loading
Contribution (%)

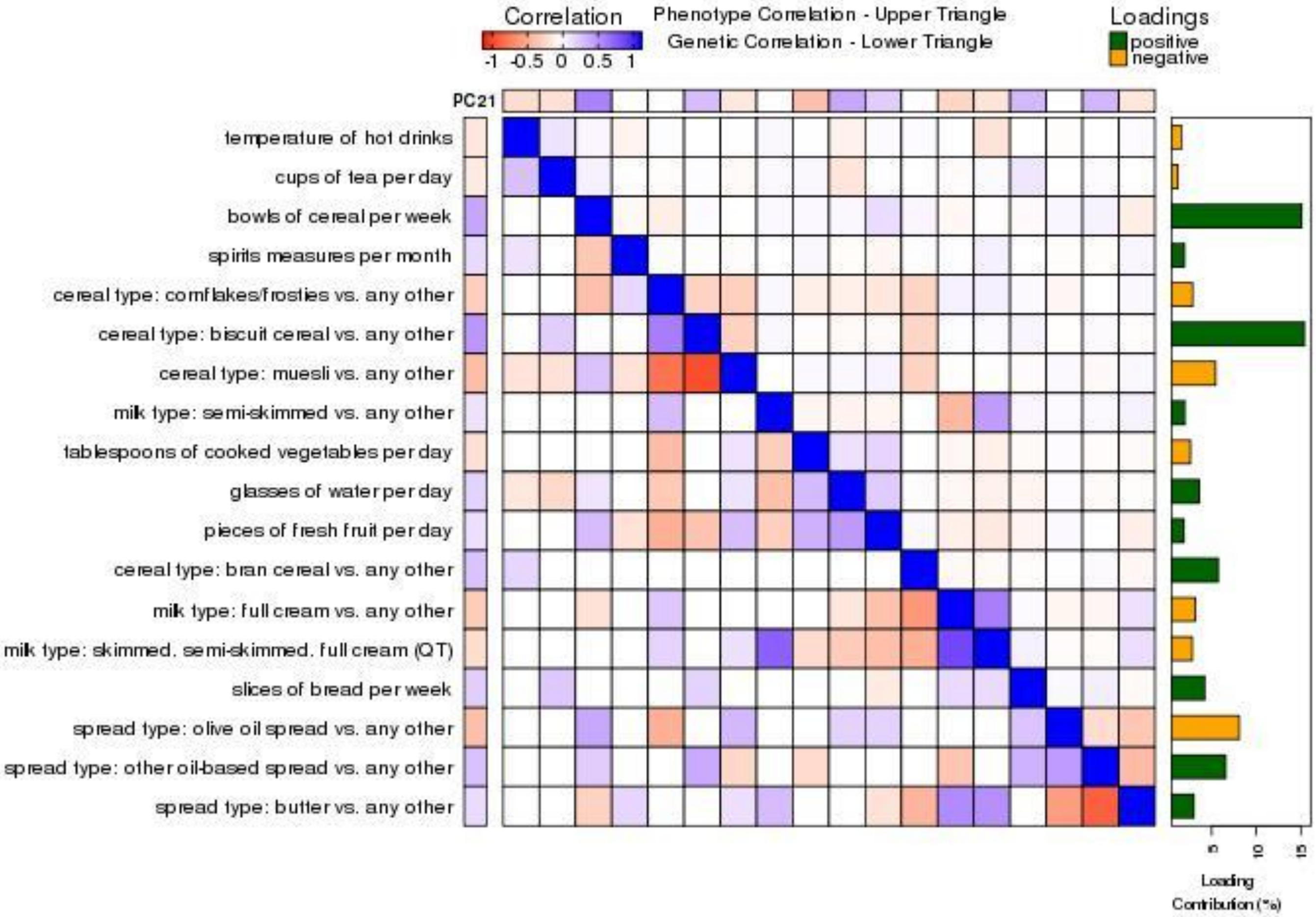
Correlation
-1 -0.5 0 0.5 1
Phenotype Correlation - Upper Triangle
Genetic Correlation - Lower Triangle
Loadings
positive
negative
PC21
temperature of hot drinks
cups of tea per day
bowls of cereal per week
spirits measures per month
cereal type: cornflakes/frosties vs. any other
cereal type: biscuit cereal vs. any other
cereal type: muesli vs. any other
milk type: semi-skimmed vs. any other
tablespoons of cooked vegetables per day
glasses of water per day
pieces of fresh fruit per day
cereal type: bran cereal vs. any other
milk type: full cream vs. any other
milk type: skimmed, semi-skimmed, full cream (QT)
slices of bread per week
spread type: olive oil spread vs. any other
spread type: other oil-based spread vs. any other
spread type: butter vs. any other
5 10 15
Loading Contribution (%)

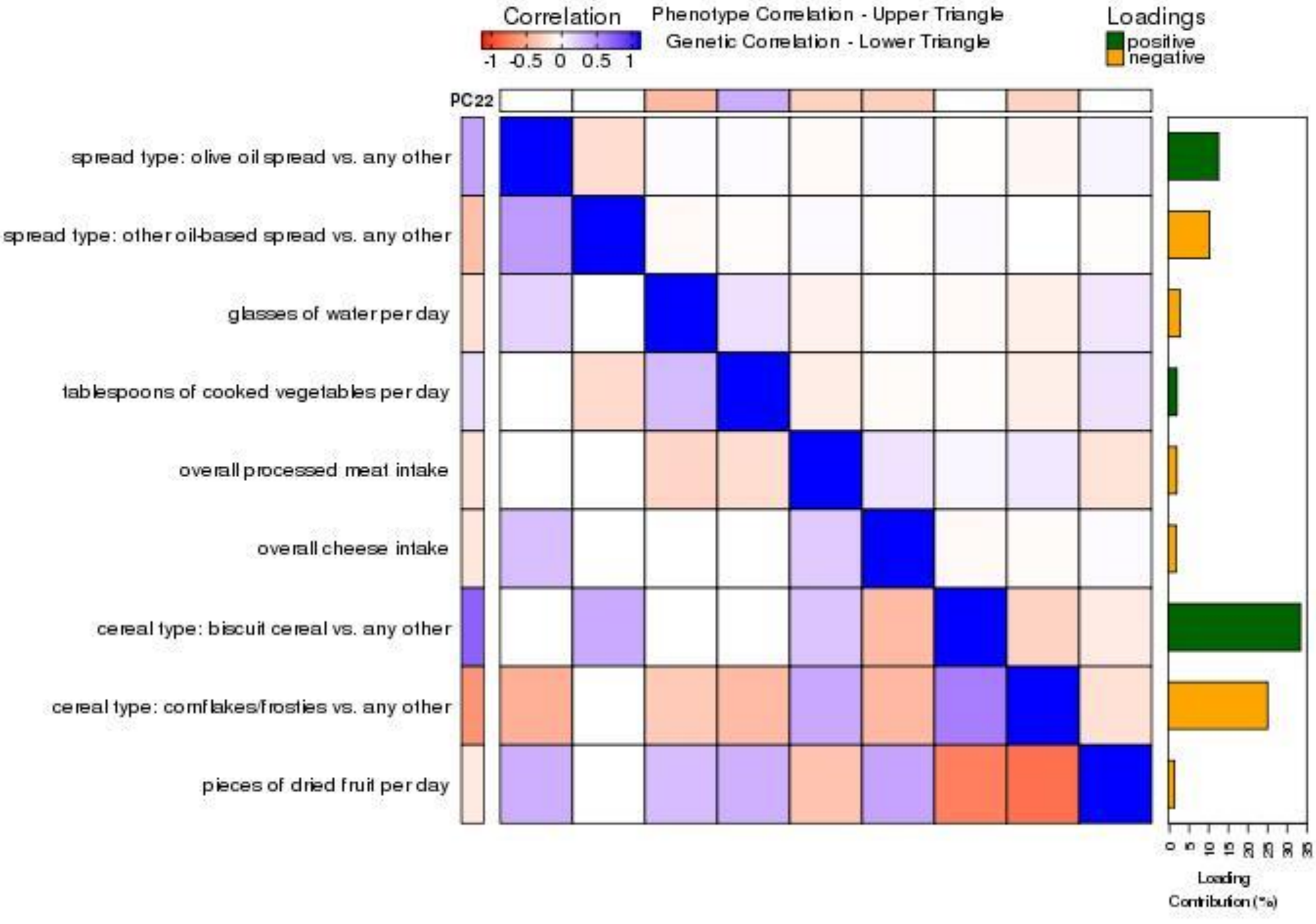
Correlation
-1 -0.5 0 0.5 1
Phenotype Correlation - Upper Triangle
Genetic Correlation - Lower Triangle
Loadings
positive
negative
PC22
spread type: olive oil spread vs. any other
spread type: other oil-based spread vs. any other
glasses of water per day
tablespoons of cooked vegetables per day
overall processed meat intake
overall cheese intake
cereal type: biscuit cereal vs. any other
cereal type: cornflakes/frosties vs. any other
pieces of dried fruit per day
0 5 10 15 20 25 30 35
Loading Contribution (%)

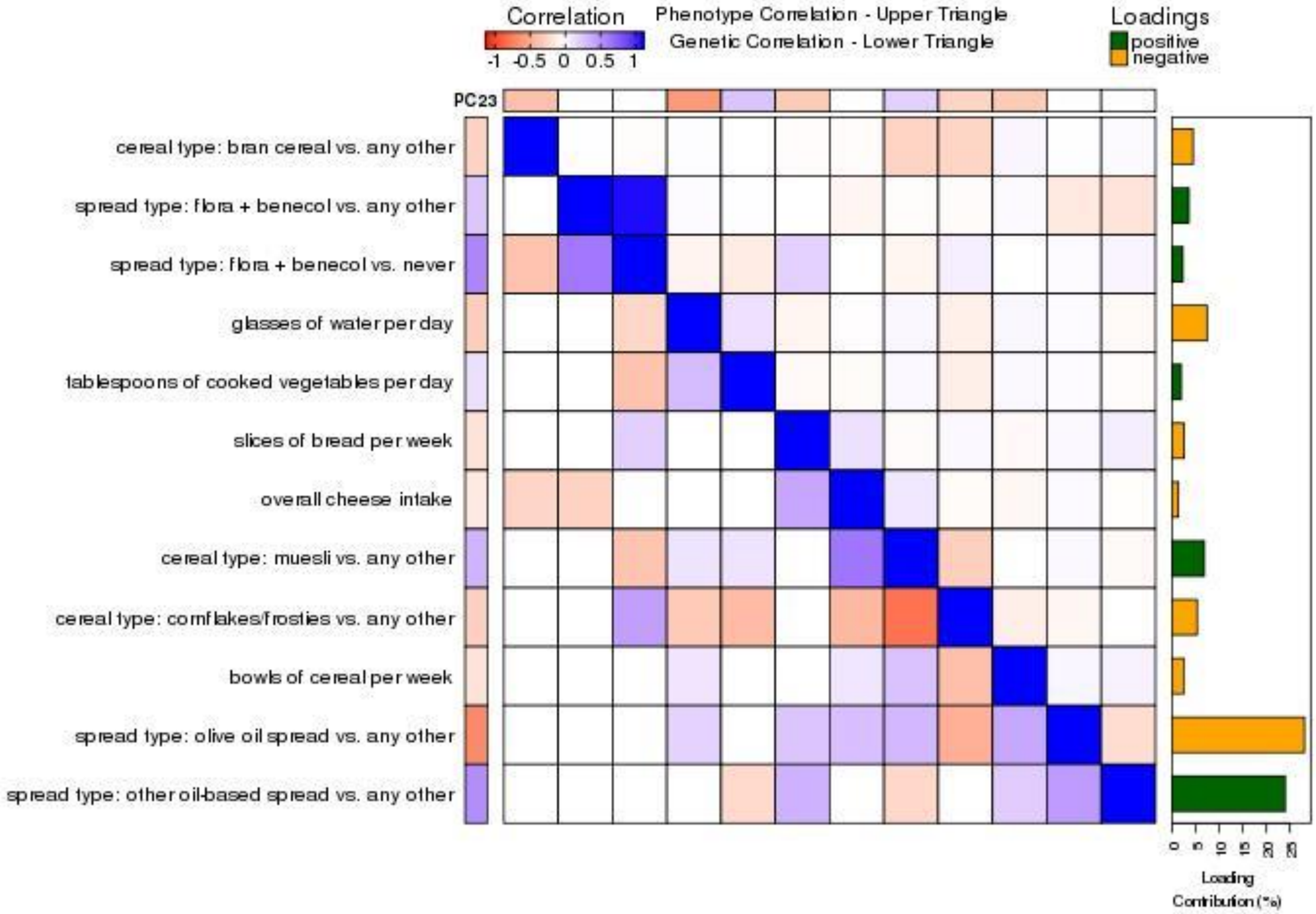
Correlation
-1 -0.5 0 0.5 1
Phenotype Correlation - Upper Triangle
Genetic Correlation - Lower Triangle
Loadings
positive
negative
PC23
cereal type: bran cereal vs. any other
spread type: flora + benecol vs. any other
spread type: flora + benecol vs. never
glasses of water per day
tablespoons of cooked vegetables per day
slices of bread per week
overall cheese intake
cereal type: muesli vs. any other
cereal type: cornflakes/frosties vs. any other
bowls of cereal per week
spread type: olive oil spread vs. any other
spread type: other oil-based spread vs. any other
0 5 10 15 20 25
Loading
Contribution (%)

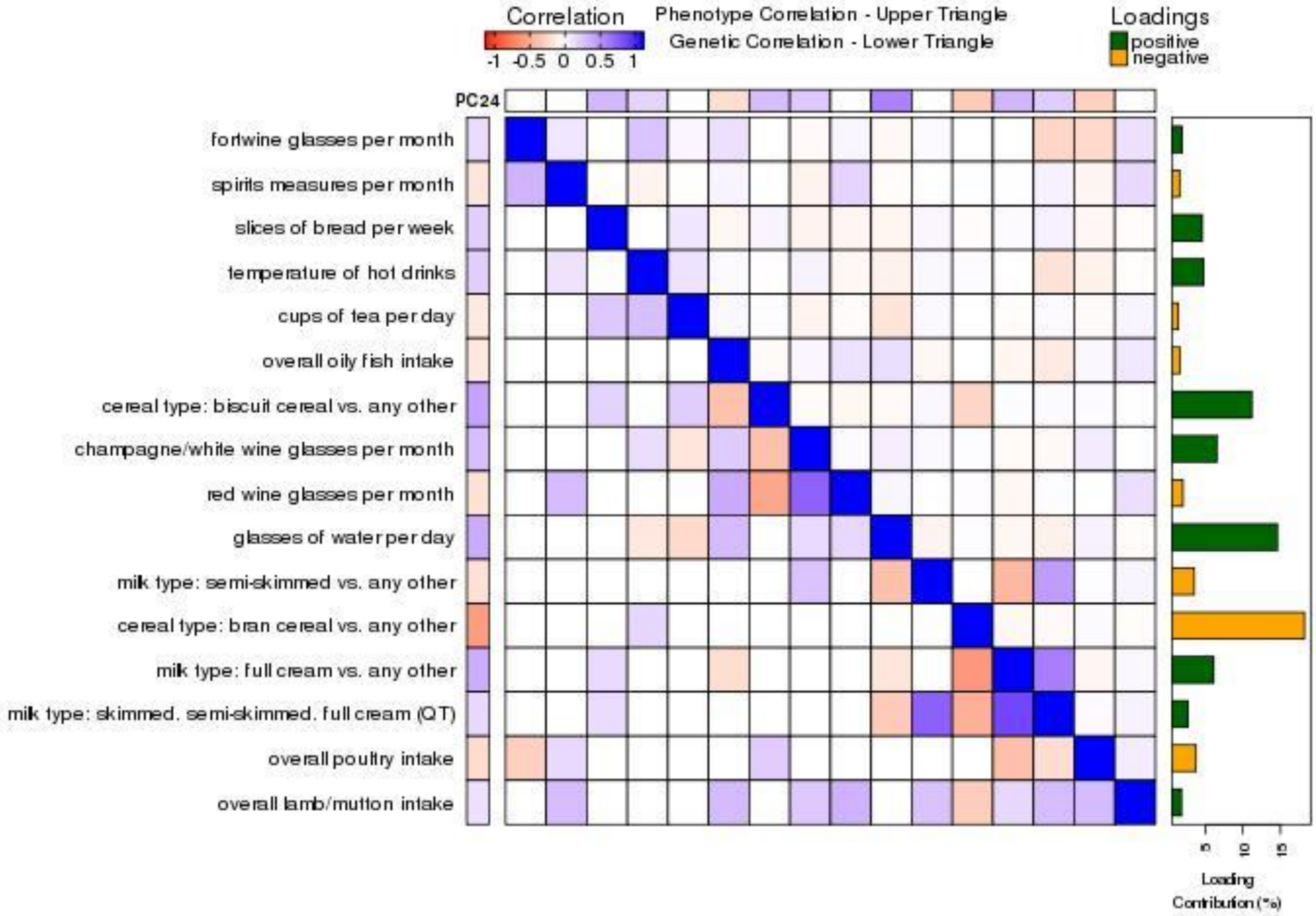
Correlation
-1 -0.5 0 0.5 1
Phenotype Correlation - Upper Triangle
Genetic Correlation - Lower Triangle
Loadings
positive
negative
PC24
fortwine glasses per month
spirits measures per month
slices of bread per week
temperature of hot drinks
cups of tea per day
overall oily fish intake
cereal type: biscuit cereal vs. any other
champagne/white wine glasses per month
red wine glasses per month
glasses of water per day
milk type: semi-skimmed vs. any other
cereal type: bran cereal vs. any other
milk type: full cream vs. any other
milk type: skimmed, semi-skimmed, full cream (QT)
overall poultry intake
overall lamb/mutton intake
5 10 15
Loading
Contribution (%)

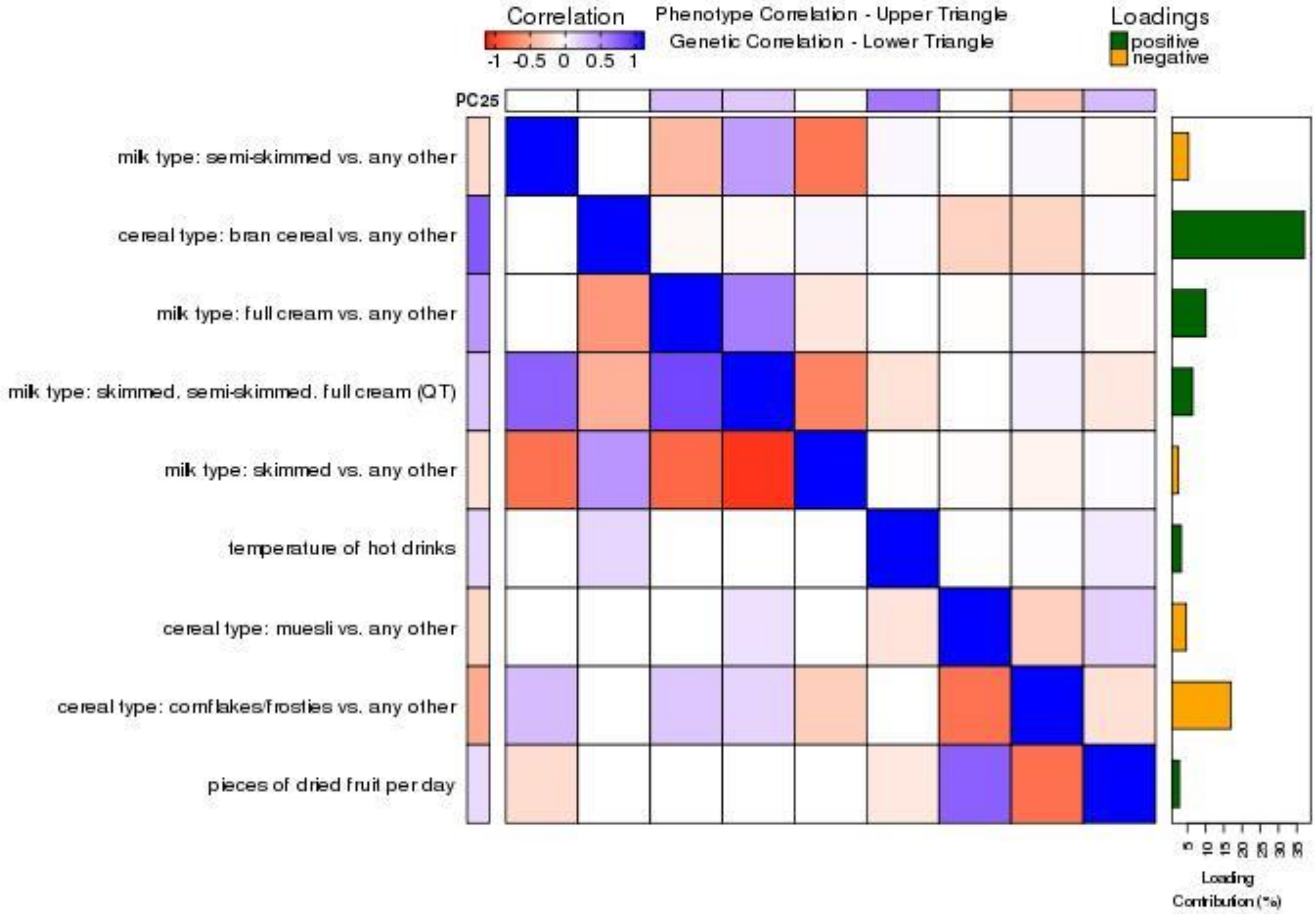
Correlation
-1 -0.5 0 0.5 1
Phenotype Correlation - Upper Triangle
Genetic Correlation - Lower Triangle
Loadings
positive
negative
PC25
milk type: semi-skimmed vs. any other
cereal type: bran cereal vs. any other
milk type: full cream vs. any other
milk type: skimmed, semi-skimmed, full cream (QT)
milk type: skimmed vs. any other
temperature of hot drinks
cereal type: muesli vs. any other
cereal type: cornflakes/frosties vs. any other
pieces of dried fruit per day
5 10 15 20 25 30 35
Loading
Contribution (%)

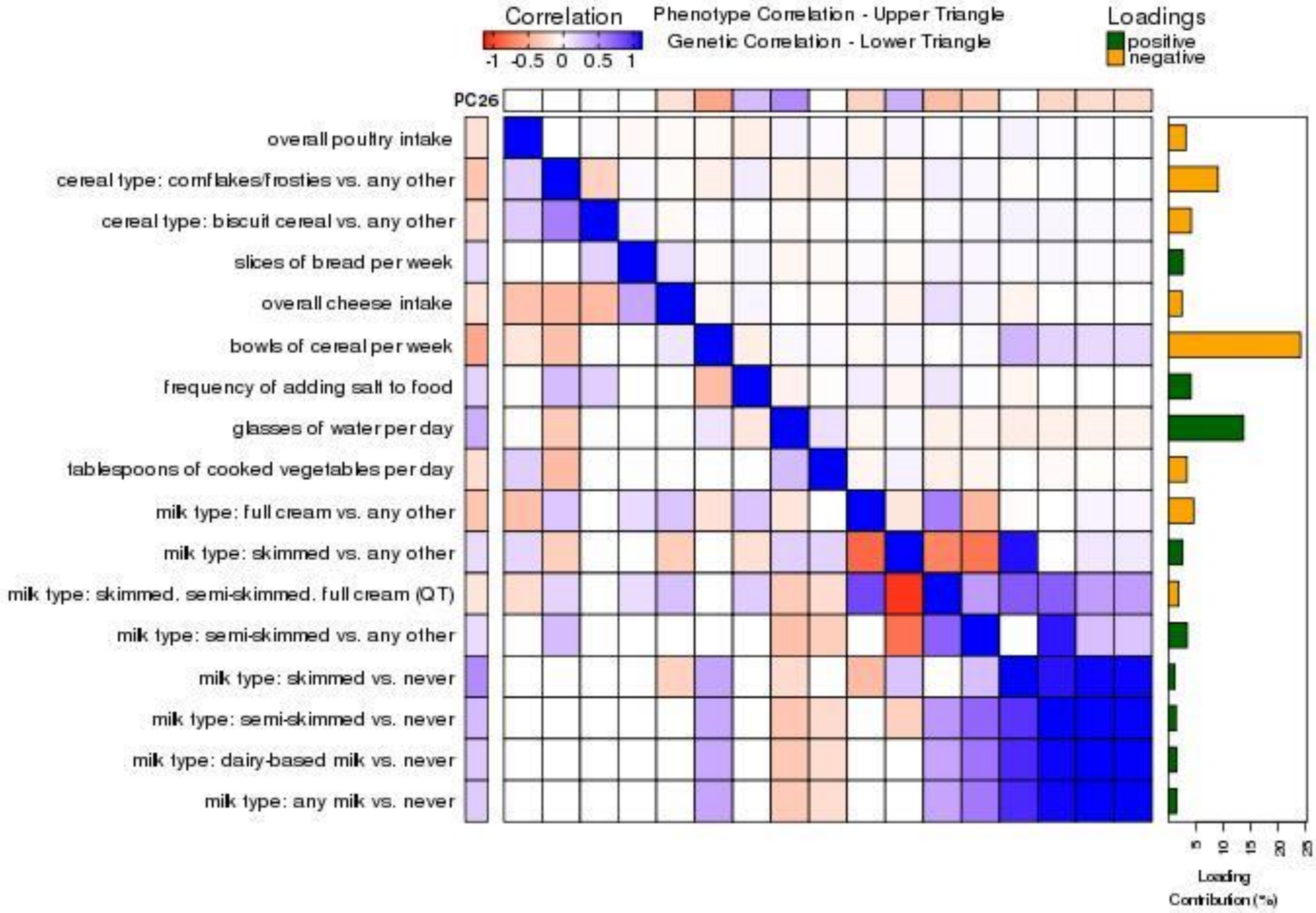
Correlation
-1 -0.5 0 0.5 1
Phenotype Correlation - Upper Triangle
Genetic Correlation - Lower Triangle
Loadings
positive
negative
PC26
overall poultry intake
cereal type: cornflakes/frosties vs. any other
cereal type: biscuit cereal vs. any other
slices of bread per week
overall cheese intake
bowls of cereal per week
frequency of adding salt to food
glasses of water per day
tablespoons of cooked vegetables per day
milk type: full cream vs. any other
milk type: skimmed vs. any other
milk type: skimmed, semi-skimmed, full cream (QT)
milk type: semi-skimmed vs. any other
milk type: skimmed vs. never
milk type: semi-skimmed vs. never
milk type: dairy-based milk vs. never
milk type: any milk vs. never
5 10 15 20 25
Loading
Contribution (%)

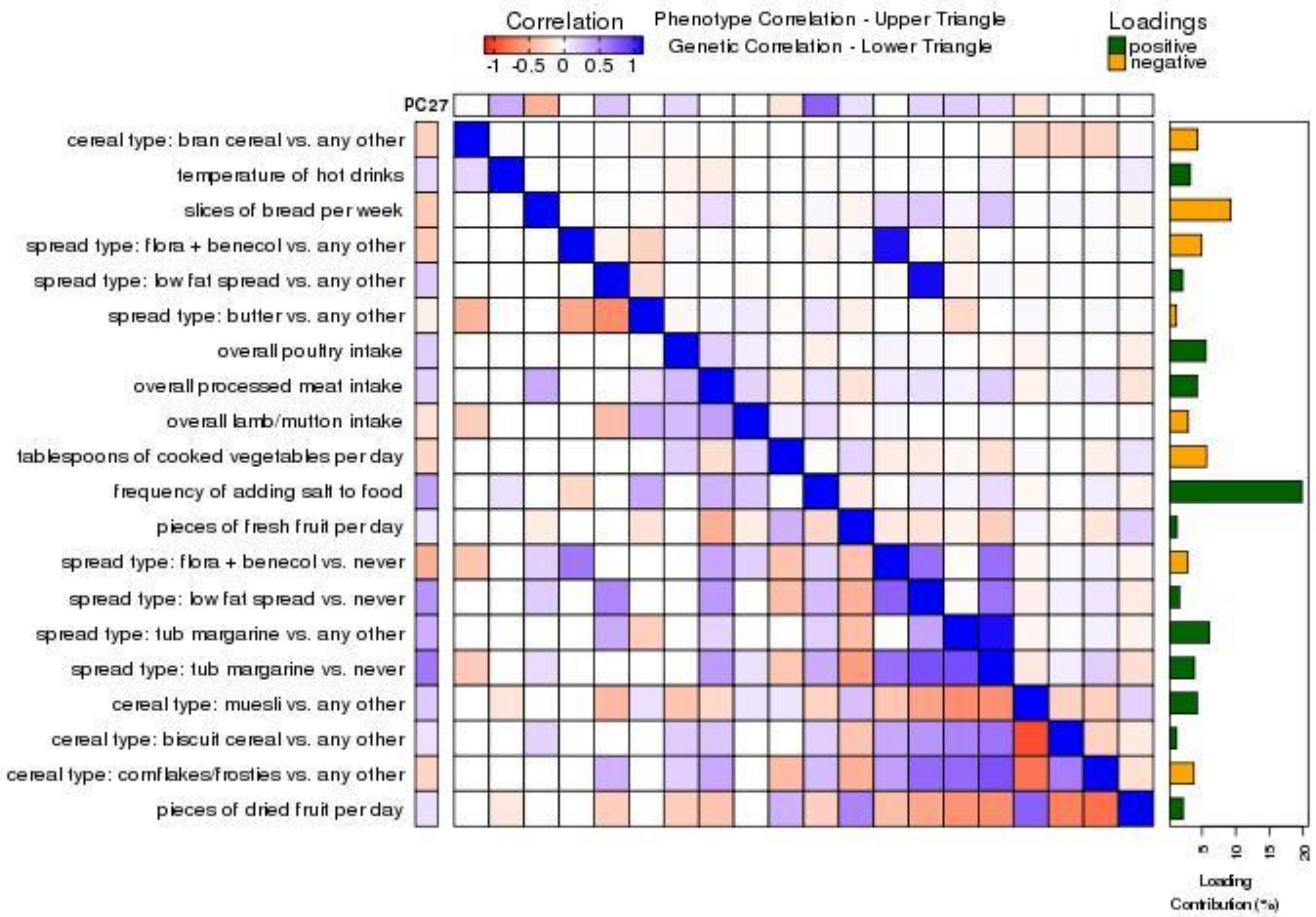
Correlation
-1 -0.5 0 0.5 1
Phenotype Correlation - Upper Triangle
Genetic Correlation - Lower Triangle
Loadings
positive
negative
PC27
cereal type: bran cereal vs. any other
temperature of hot drinks
slices of bread per week
spread type: flora + benecol vs. any other
spread type: low fat spread vs. any other
spread type: butter vs. any other
overall poultry intake
overall processed meat intake
overall lamb/mutton intake
tablespoons of cooked vegetables per day
frequency of adding salt to food
pieces of fresh fruit per day
spread type: flora + benecol vs. never
spread type: low fat spread vs. never
spread type: tub margarine vs. any other
spread type: tub margarine vs. never
cereal type: muesli vs. any other
cereal type: biscuit cereal vs. any other
cereal type: cornflakes/frosties vs. any other
pieces of dried fruit per day
5
10
15
20
Loading Contribution (%)

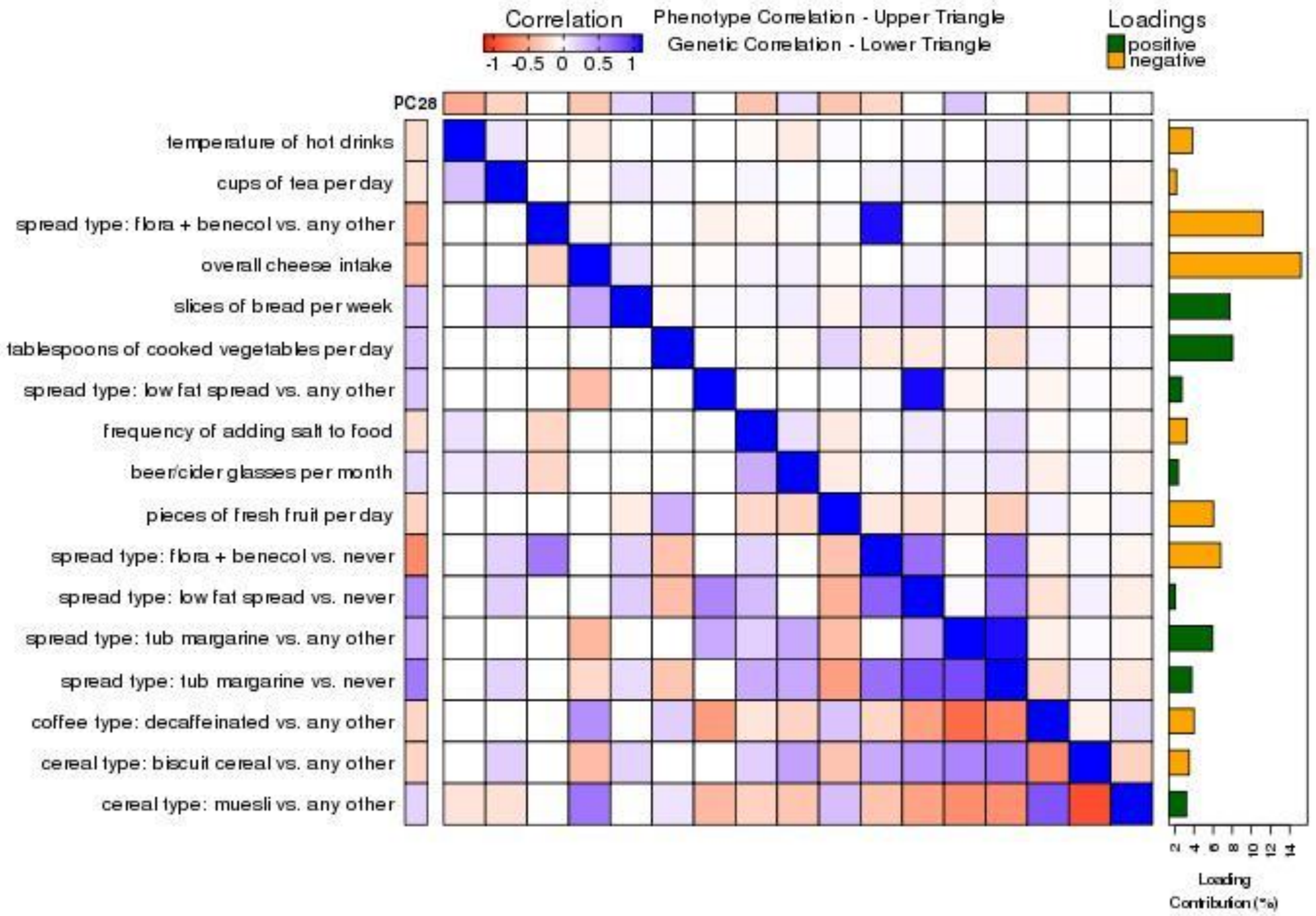
Correlation
-1 -0.5 0 0.5 1
Phenotype Correlation - Upper Triangle
Genetic Correlation - Lower Triangle
Loadings
positive
negative
PC28
temperature of hot drinks
cups of tea per day
spread type: flora + benecol vs. any other
overall cheese intake
slices of bread per week
tablespoons of cooked vegetables per day
spread type: low fat spread vs. any other
frequency of adding salt to food
beer/cider glasses per month
pieces of fresh fruit per day
spread type: flora + benecol vs. never
spread type: low fat spread vs. never
spread type: tub margarine vs. any other
spread type: tub margarine vs. never
coffee type: decaffeinated vs. any other
cereal type: biscuit cereal vs. any other
cereal type: muesli vs. any other
2
4
6
8
10
12
14
Loading Contribution (%)

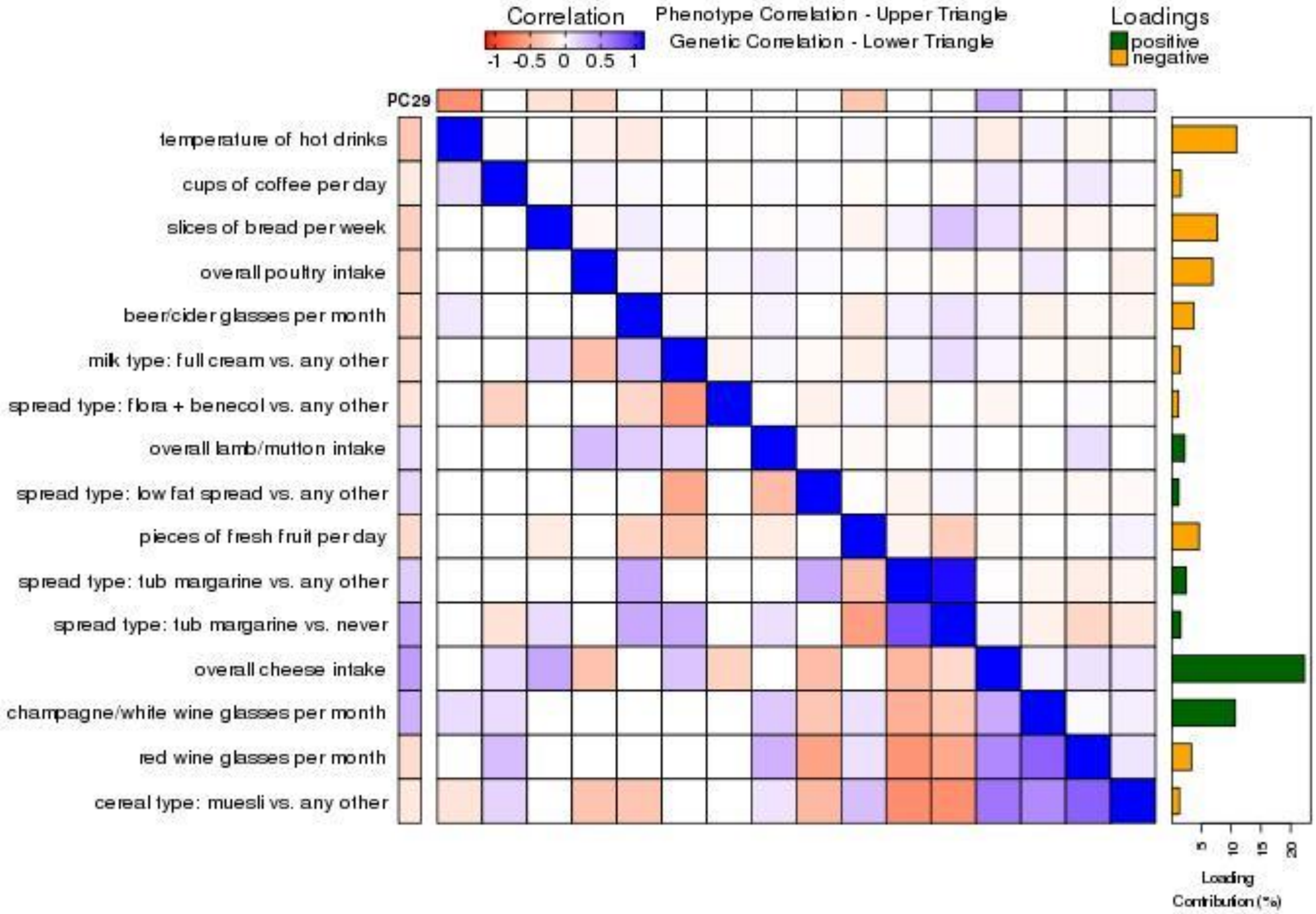
Correlation
-1 -0.5 0 0.5 1
Phenotype Correlation - Upper Triangle
Genetic Correlation - Lower Triangle
Loadings
positive
negative
PC29
temperature of hot drinks
cups of coffee per day
slices of bread per week
overall poultry intake
beer/cider glasses per month
milk type: full cream vs. any other
spread type: flora + benecol vs. any other
overall lamb/mutton intake
spread type: low fat spread vs. any other
pieces of fresh fruit per day
spread type: tub margarine vs. any other
spread type: tub margarine vs. never
overall cheese intake
champagne/white wine glasses per month
red wine glasses per month
cereal type: muesli vs. any other
5 10 15 20
Loading Contribution (%)

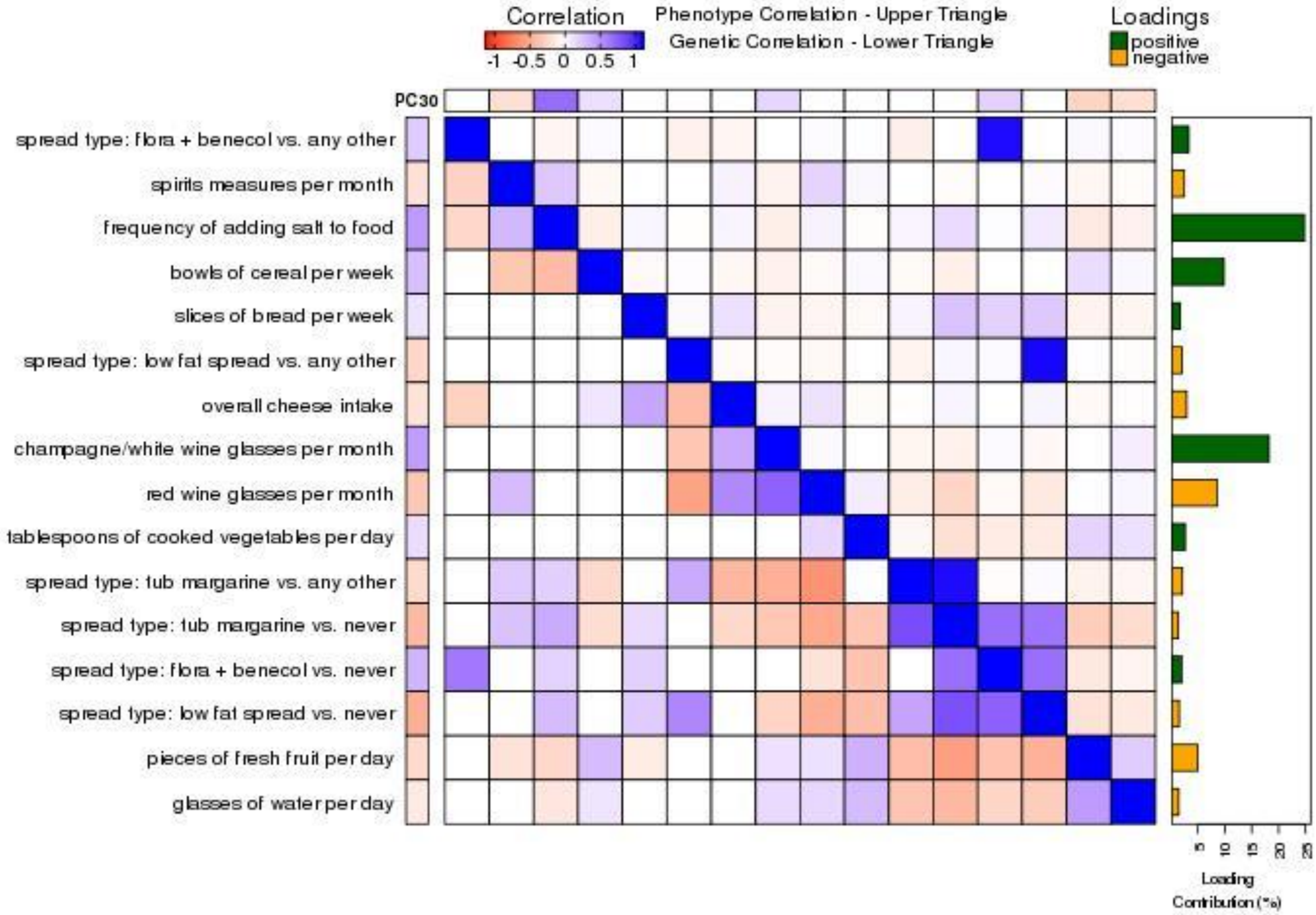
Correlation
-1 -0.5 0 0.5 1
Phenotype Correlation - Upper Triangle
Genetic Correlation - Lower Triangle
Loadings
positive
negative
PC30
spread type: flora + benecol vs. any other
spirits measures per month
frequency of adding salt to food
bowls of cereal per week
slices of bread per week
spread type: low fat spread vs. any other
overall cheese intake
champagne/white wine glasses per month
red wine glasses per month
tablespoons of cooked vegetables per day
spread type: tub margarine vs. any other
spread type: tub margarine vs. never
spread type: flora + benecol vs. never
spread type: low fat spread vs. never
pieces of fresh fruit per day
glasses of water per day
5 10 15 20 25
Loading Contribution (%)

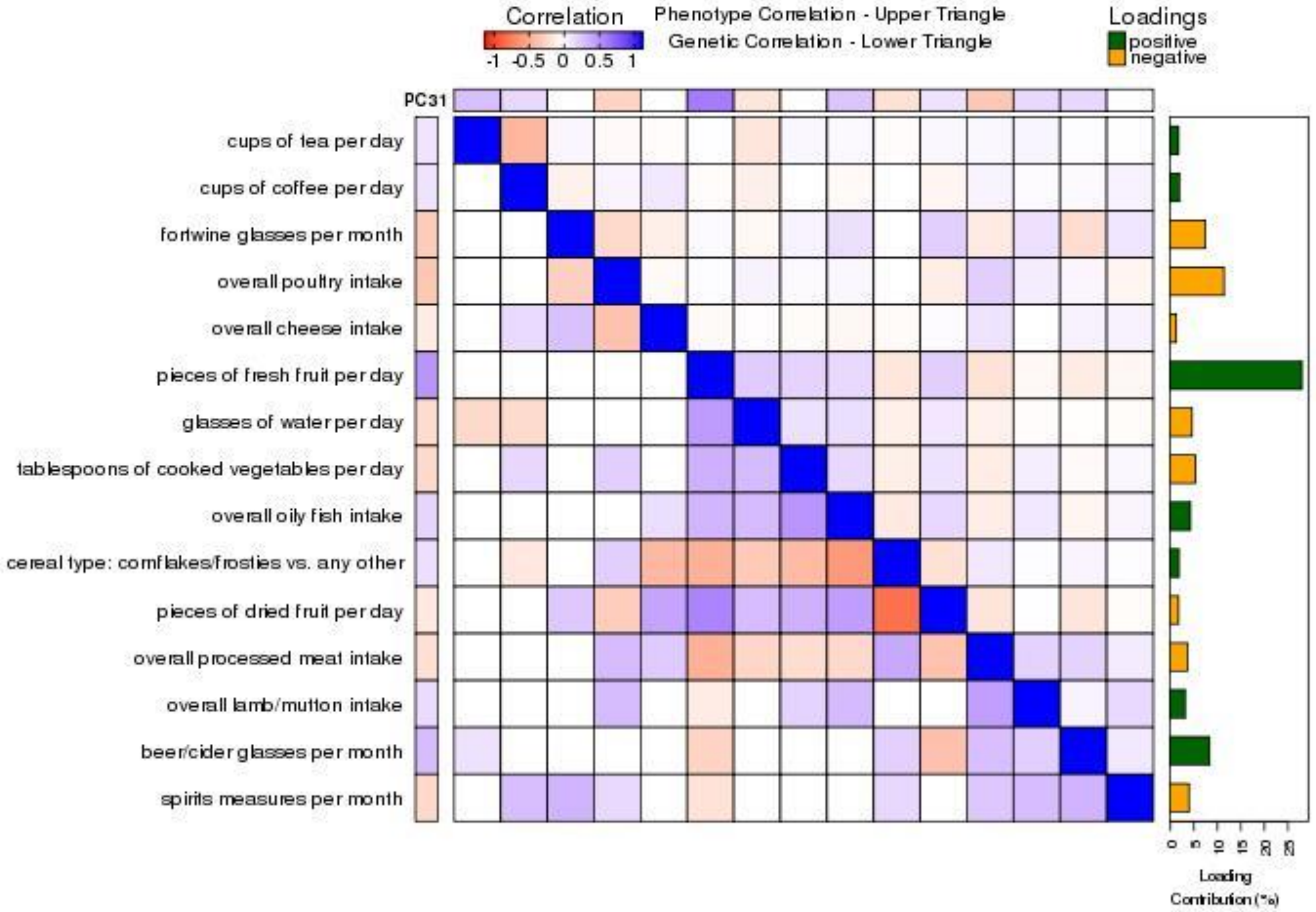
Correlation
-1 -0.5 0 0.5 1
Phenotype Correlation - Upper Triangle
Genetic Correlation - Lower Triangle
Loadings
positive
negative
PC31
cups of tea per day
cups of coffee per day
fortwine glasses per month
overall poultry intake
overall cheese intake
pieces of fresh fruit per day
glasses of water per day
tablespoons of cooked vegetables per day
overall oily fish intake
cereal type: cornflakes/frosties vs. any other
pieces of dried fruit per day
overall processed meat intake
overall lamb/mutton intake
beer/cider glasses per month
spirits measures per month
Loading Contribution (%)

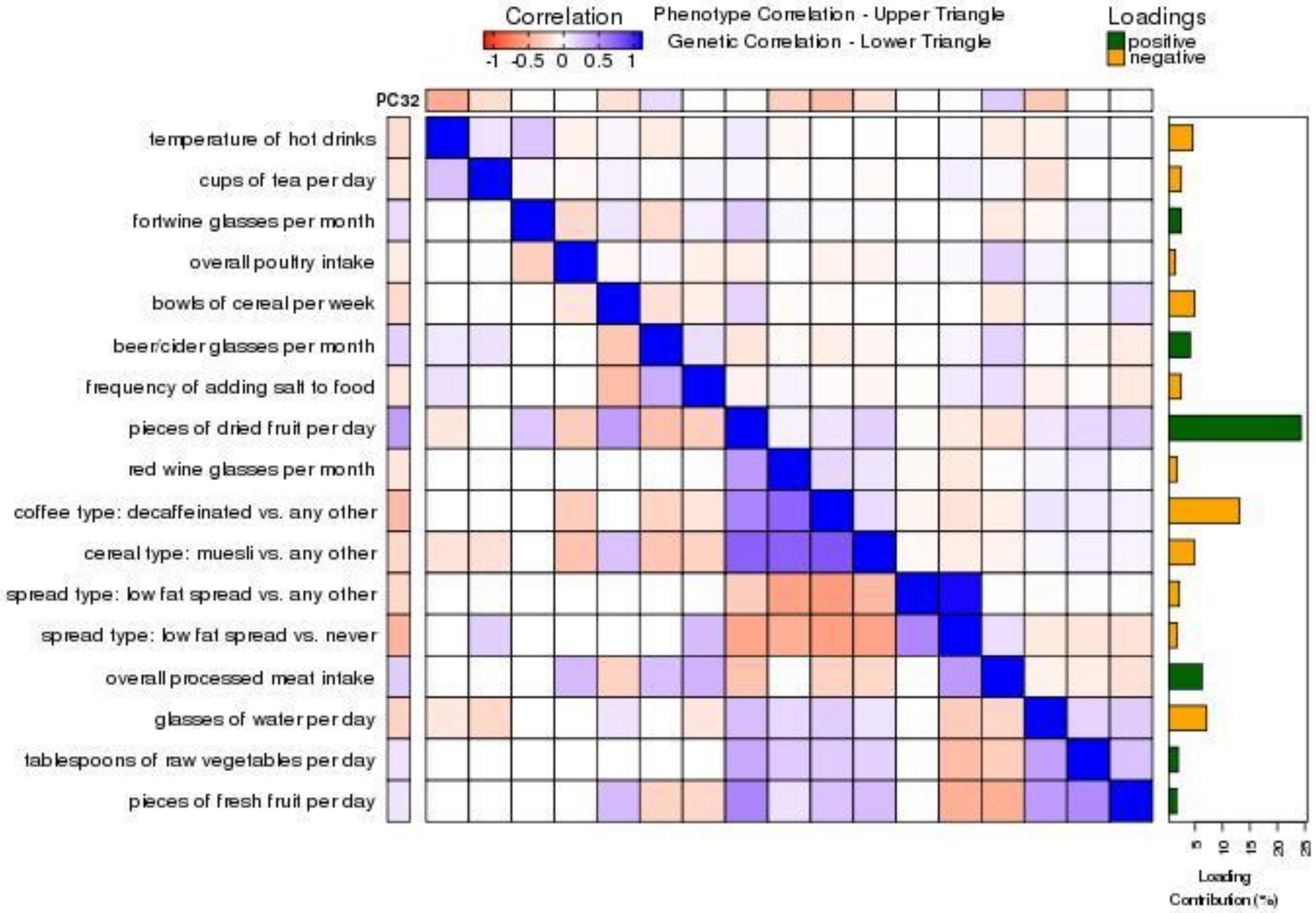
Correlation
-1 -0.5 0 0.5 1
Phenotype Correlation - Upper Triangle
Genetic Correlation - Lower Triangle
Loadings
positive
negative
PC32
temperature of hot drinks
cups of tea per day
fortwine glasses per month
overall poultry intake
bowls of cereal per week
beer/cider glasses per month
frequency of adding salt to food
pieces of dried fruit per day
red wine glasses per month
coffee type: decaffeinated vs. any other
cereal type: muesli vs. any other
spread type: low fat spread vs. any other
spread type: low fat spread vs. never
overall processed meat intake
glasses of water per day
tablespoons of raw vegetables per day
pieces of fresh fruit per day
Loading Contribution (%)

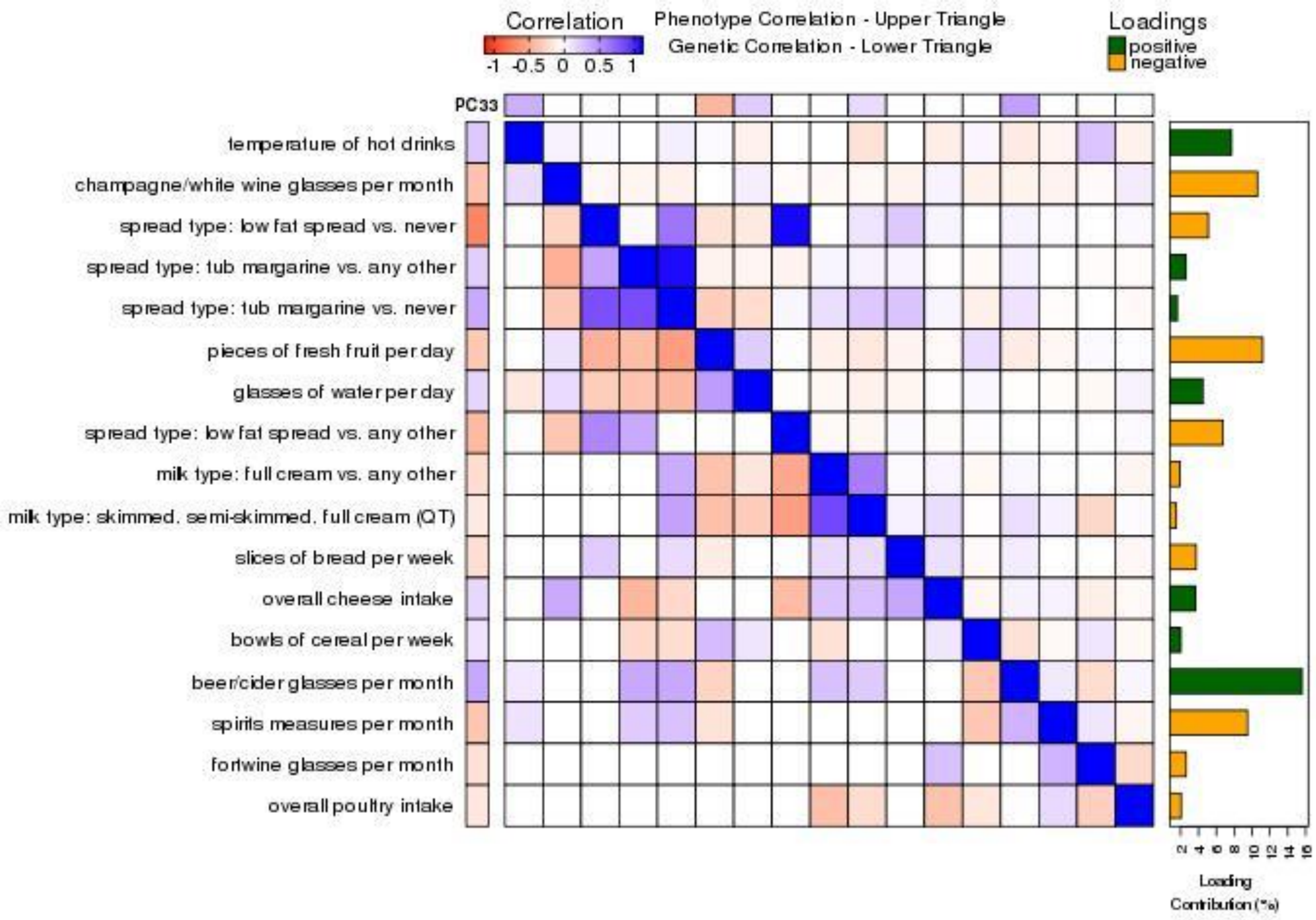
Correlation
-1 -0.5 0 0.5 1
Phenotype Correlation - Upper Triangle
Genetic Correlation - Lower Triangle
Loadings
positive
negative
PC33
temperature of hot drinks
champagne/white wine glasses per month
spread type: low fat spread vs. never
spread type: tub margarine vs. any other
spread type: tub margarine vs. never
pieces of fresh fruit per day
glasses of water per day
spread type: low fat spread vs. any other
milk type: full cream vs. any other
milk type: skimmed, semi-skimmed, full cream (QT)
slices of bread per week
overall cheese intake
bowls of cereal per week
beer/cider glasses per month
spirits measures per month
fortwine glasses per month
overall poultry intake
2 4 6 8 10 12 14 16
Loading Contribution (%)

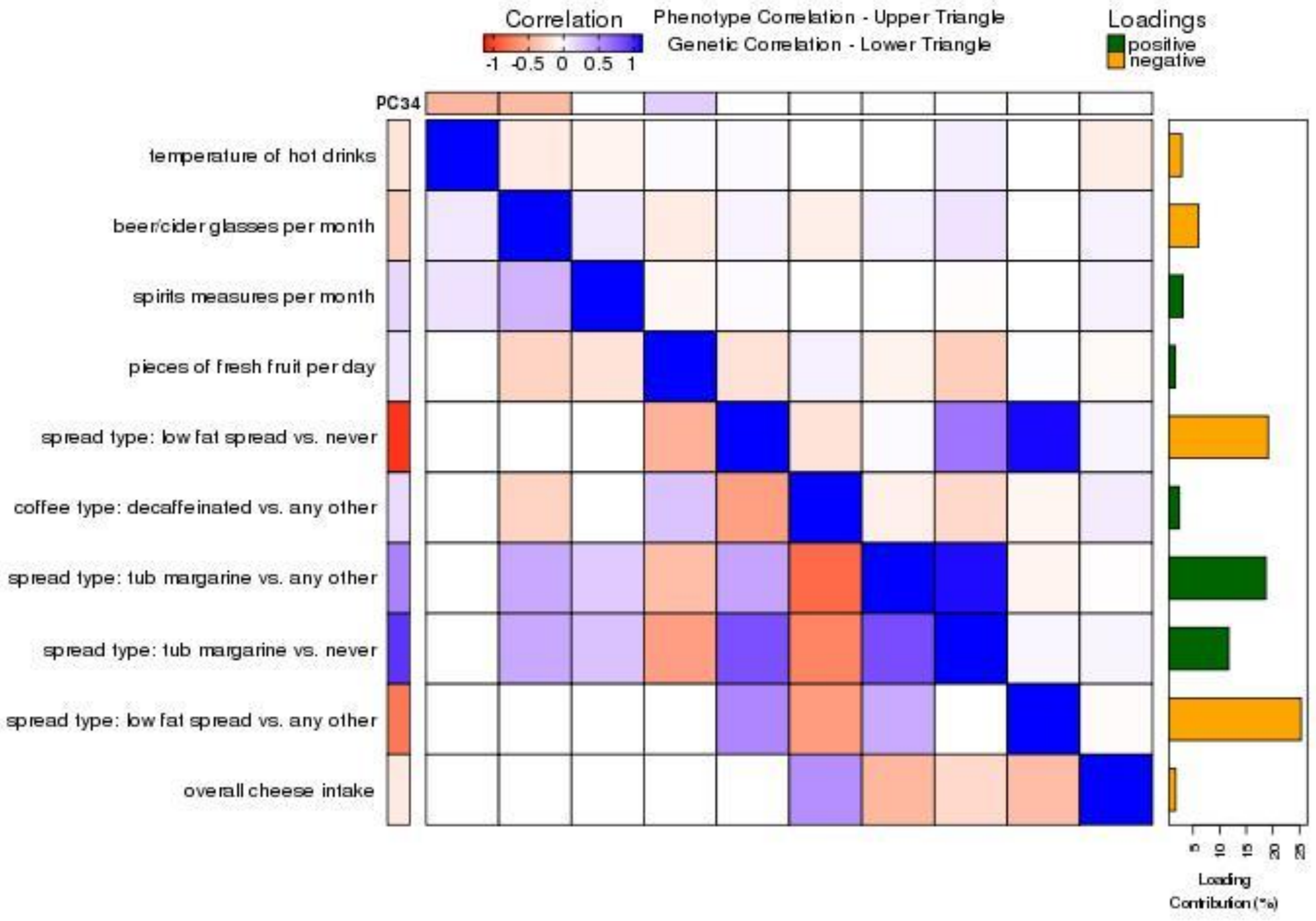
Correlation
-1 -0.5 0 0.5 1
Phenotype Correlation - Upper Triangle
Genetic Correlation - Lower Triangle
Loadings
positive
negative
PC34
temperature of hot drinks
beer/cider glasses per month
spirits measures per month
pieces of fresh fruit per day
spread type: low fat spread vs. never
coffee type: decaffeinated vs. any other
spread type: tub margarine vs. any other
spread type: tub margarine vs. never
spread type: low fat spread vs. any other
overall cheese intake
5 10 15 20 25
Loading Contribution (%)

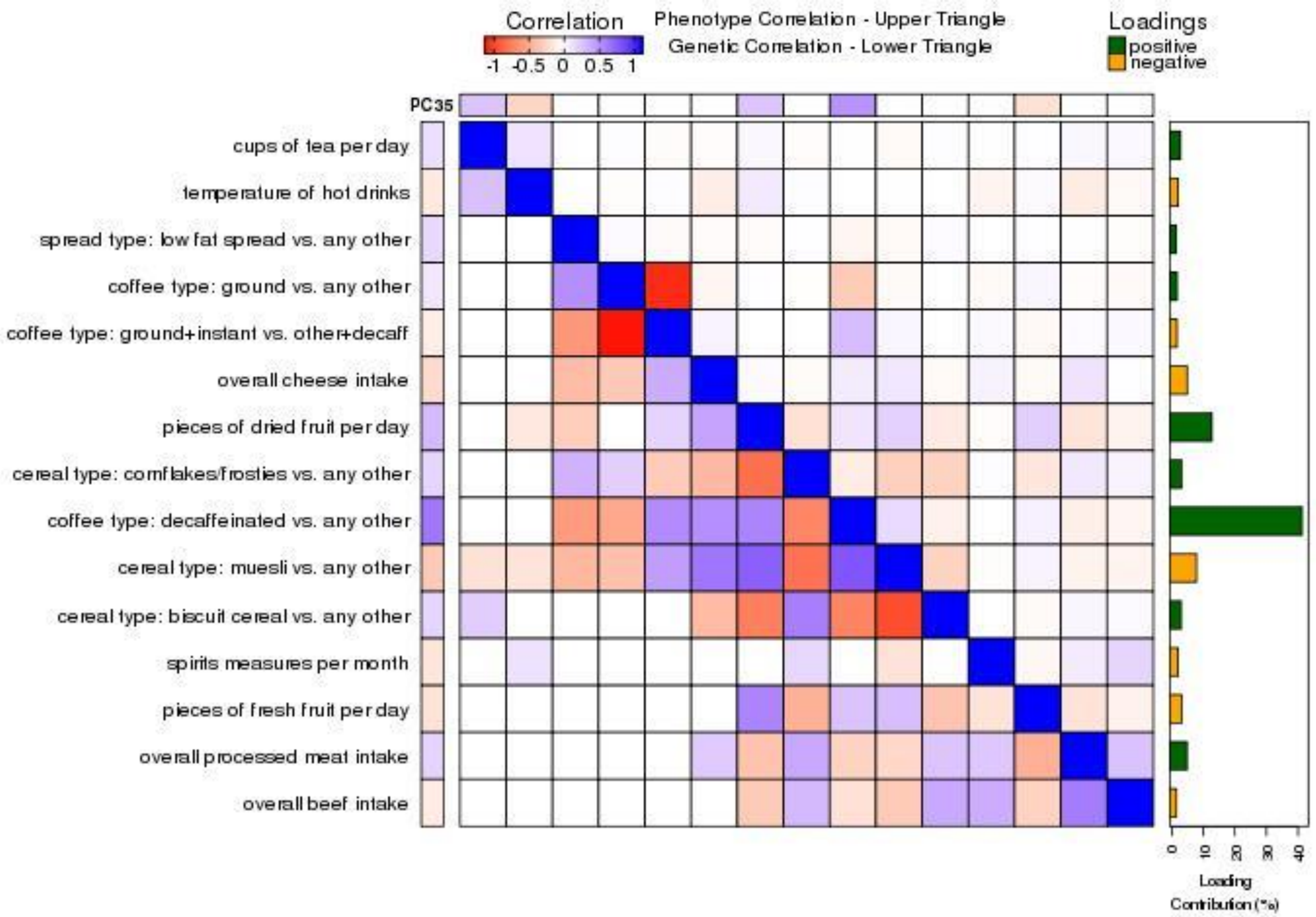
Correlation
-1 -0.5 0 0.5 1
Phenotype Correlation - Upper Triangle
Genetic Correlation - Lower Triangle
Loadings
positive
negative
PC35
cups of tea per day
temperature of hot drinks
spread type: low fat spread vs. any other
coffee type: ground vs. any other
coffee type: ground+instant vs. other+decaff
overall cheese intake
pieces of dried fruit per day
cereal type: cornflakes/frosties vs. any other
coffee type: decaffeinated vs. any other
cereal type: muesli vs. any other
cereal type: biscuit cereal vs. any other
spirits measures per month
pieces of fresh fruit per day
overall processed meat intake
overall beef intake
0 10 20 30 40
Loading
Contribution (%)

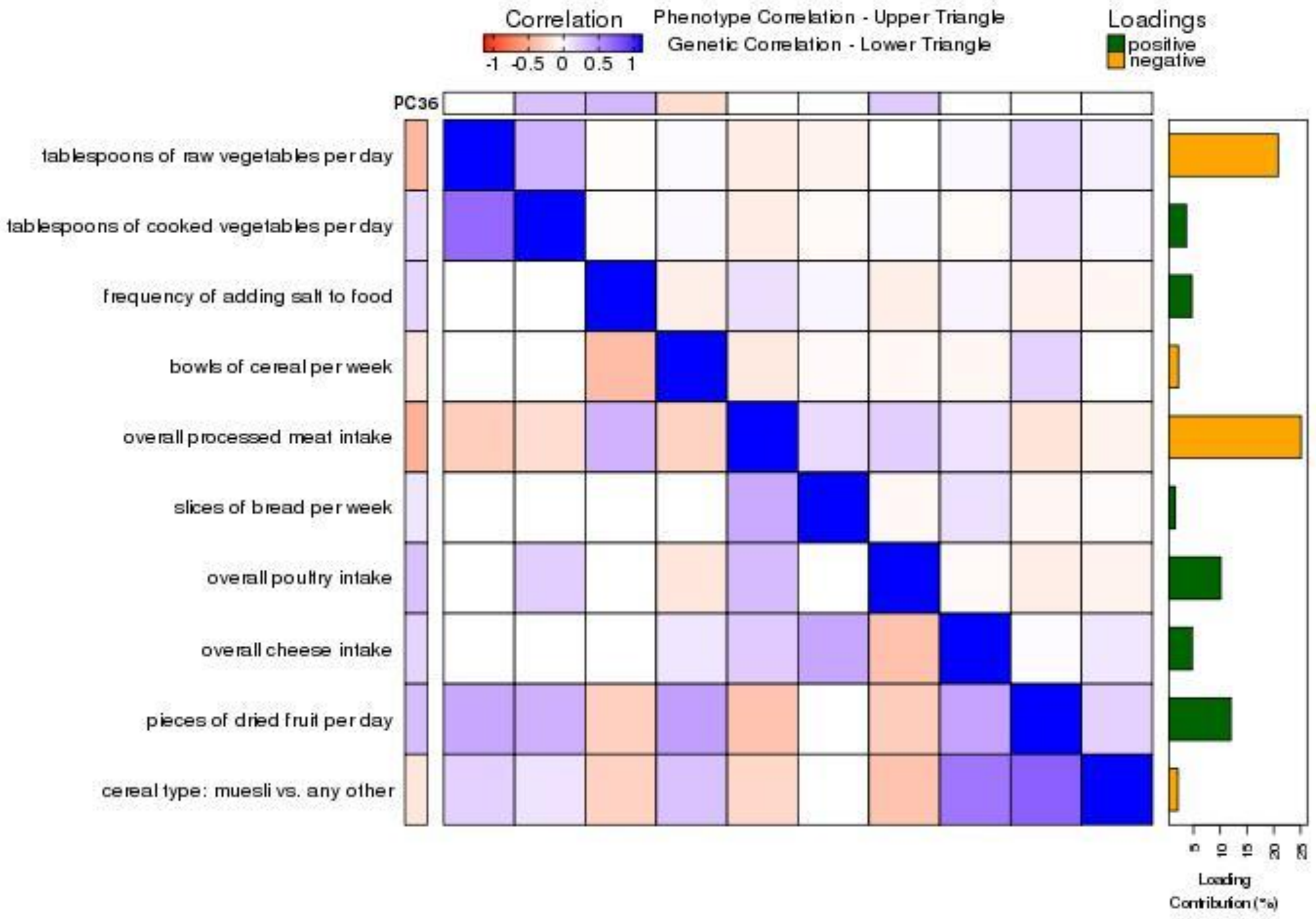
Correlation
-1 -0.5 0 0.5 1
Phenotype Correlation - Upper Triangle
Genetic Correlation - Lower Triangle
Loadings
positive
negative
PC36
tablespoons of raw vegetables per day
tablespoons of cooked vegetables per day
frequency of adding salt to food
bowls of cereal per week
overall processed meat intake
slices of bread per week
overall poultry intake
overall cheese intake
pieces of dried fruit per day
cereal type: muesli vs. any other
5 10 15 20 25
Loading
Contribution (%)

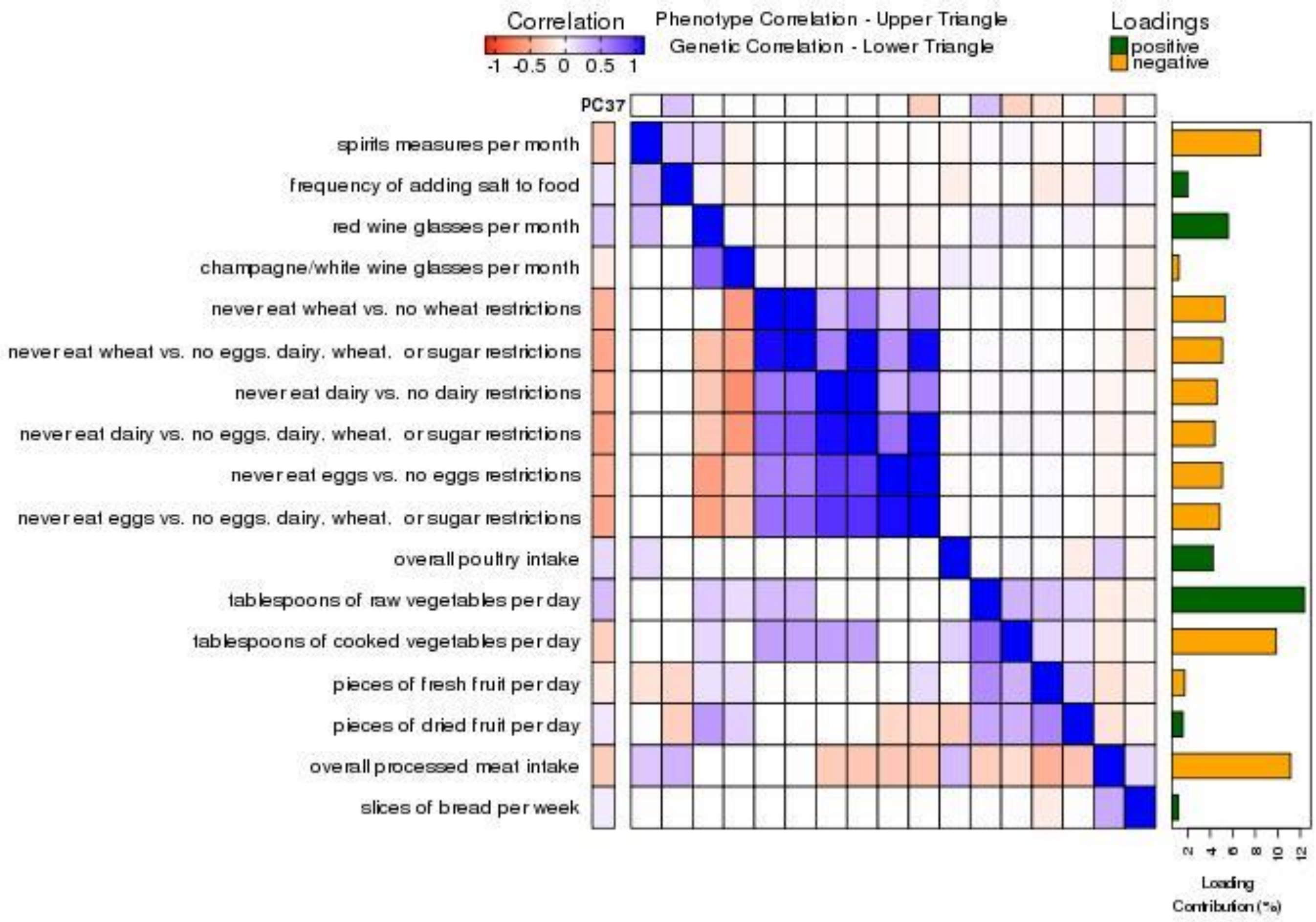
Correlation
-1 -0.5 0 0.5 1
Phenotype Correlation - Upper Triangle
Genetic Correlation - Lower Triangle
Loadings
positive
negative
PC37
spirits measures per month
frequency of adding salt to food
red wine glasses per month
champagne/white wine glasses per month
never eat wheat vs. no wheat restrictions
never eat wheat vs. no eggs, dairy, wheat, or sugar restrictions
never eat dairy vs. no dairy restrictions
never eat dairy vs. no eggs, dairy, wheat, or sugar restrictions
never eat eggs vs. no eggs restrictions
never eat eggs vs. no eggs, dairy, wheat, or sugar restrictions
overall poultry intake
tablespoons of raw vegetables per day
tablespoons of cooked vegetables per day
pieces of fresh fruit per day
pieces of dried fruit per day
overall processed meat intake
slices of bread per week
2 4 6 8 10 12
Loading Contribution (%)

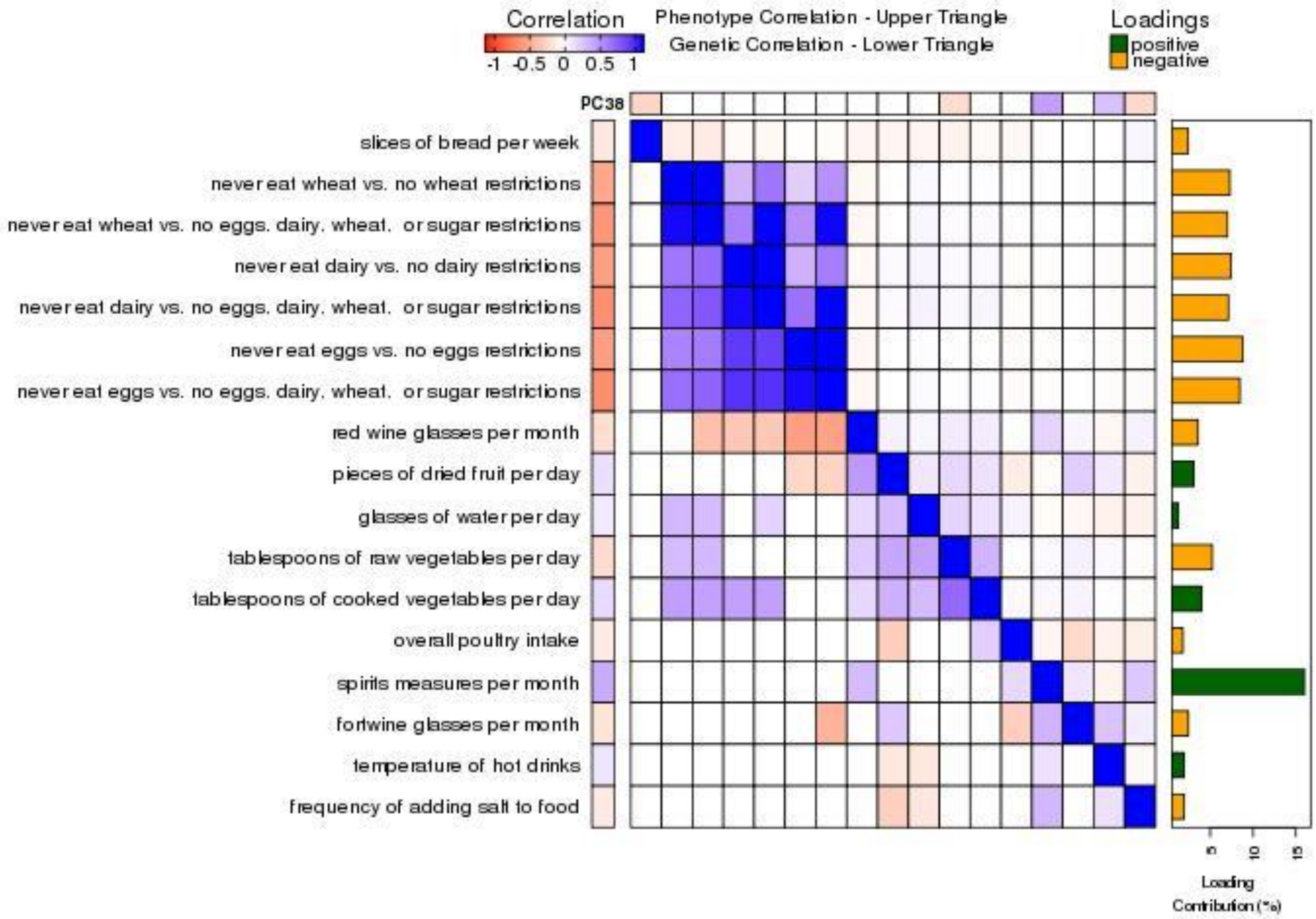
Correlation
-1 -0.5 0 0.5 1
Phenotype Correlation - Upper Triangle
Genetic Correlation - Lower Triangle
Loadings
positive
negative
PC38
slices of bread per week
never eat wheat vs. no wheat restrictions
never eat wheat vs. no eggs, dairy, wheat, or sugar restrictions
never eat dairy vs. no dairy restrictions
never eat dairy vs. no eggs, dairy, wheat, or sugar restrictions
never eat eggs vs. no eggs restrictions
never eat eggs vs. no eggs, dairy, wheat, or sugar restrictions
red wine glasses per month
pieces of dried fruit per day
glasses of water per day
tablespoons of raw vegetables per day
tablespoons of cooked vegetables per day
overall poultry intake
spirits measures per month
fortwine glasses per month
temperature of hot drinks
frequency of adding salt to food
5 10 15
Loading Contribution (%)

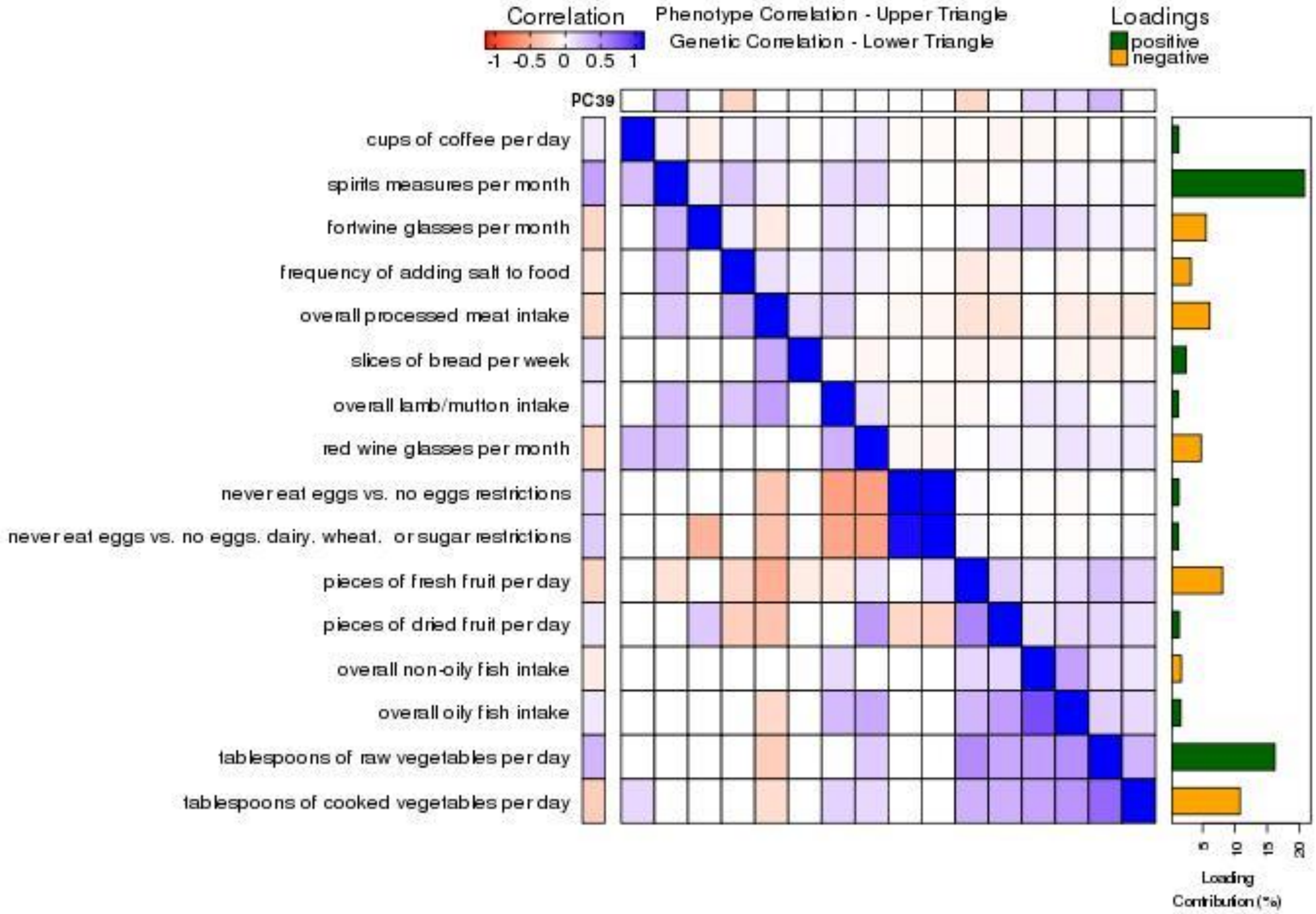
Correlation
-1 -0.5 0 0.5 1
Phenotype Correlation - Upper Triangle
Genetic Correlation - Lower Triangle
Loadings
positive
negative
PC39
cups of coffee per day
spirits measures per month
fortwine glasses per month
frequency of adding salt to food
overall processed meat intake
slices of bread per week
overall lamb/mutton intake
red wine glasses per month
never eat eggs vs. no eggs restrictions
never eat eggs vs. no eggs, dairy, wheat, or sugar restrictions
pieces of fresh fruit per day
pieces of dried fruit per day
overall non-oily fish intake
overall oily fish intake
tablespoons of raw vegetables per day
tablespoons of cooked vegetables per day
5 10 15 20
Loading
Contribution (%)

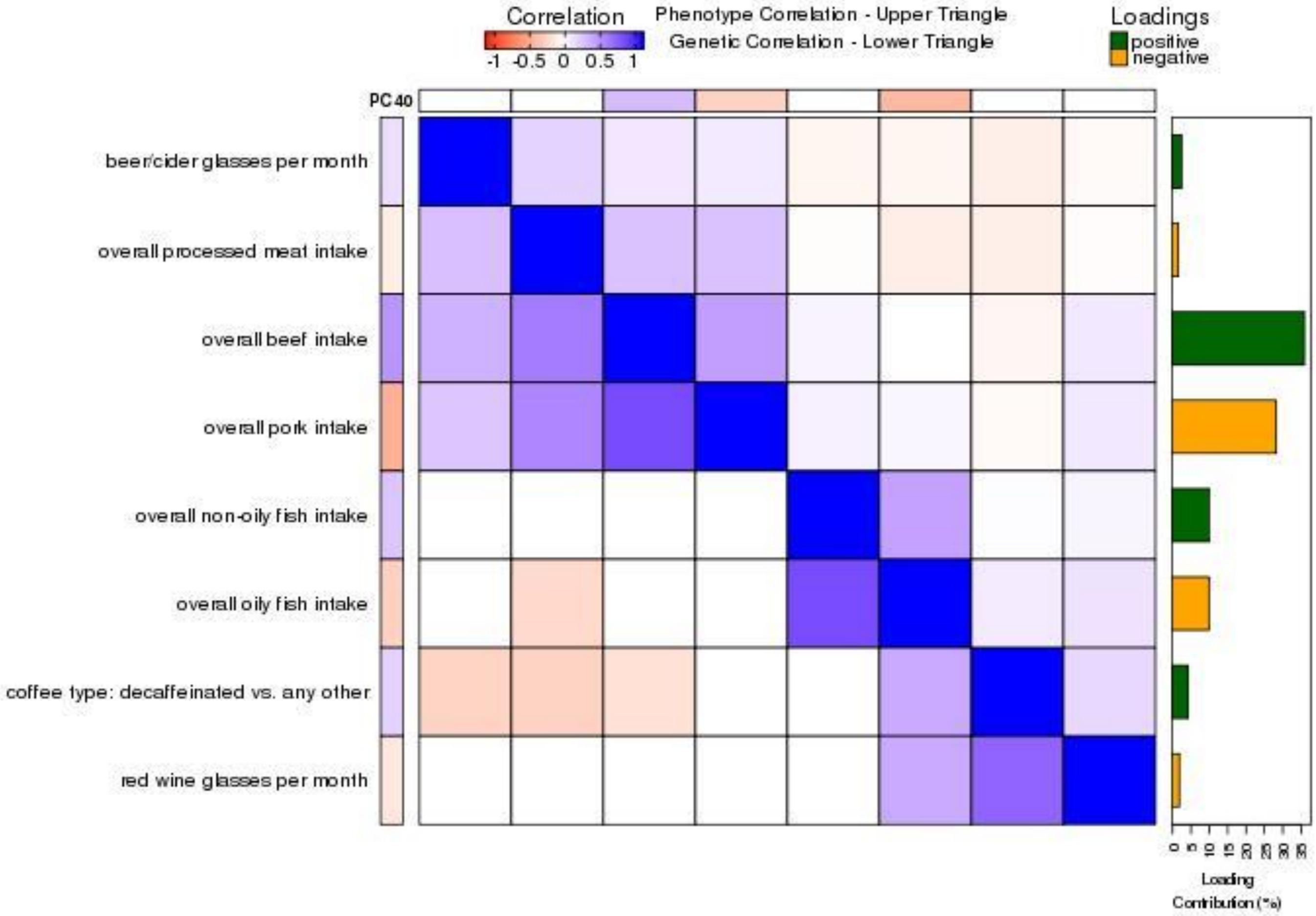
Correlation
-1 -0.5 0 0.5 1
Phenotype Correlation - Upper Triangle
Genetic Correlation - Lower Triangle
Loadings
positive
negative
PC40
beer/cider glasses per month
overall processed meat intake
overall beef intake
overall pork intake
overall non-oily fish intake
overall oily fish intake
coffee type: decaffeinated vs. any other
red wine glasses per month
0 5 10 15 20 25 30 35
Loading
Contribution (%)

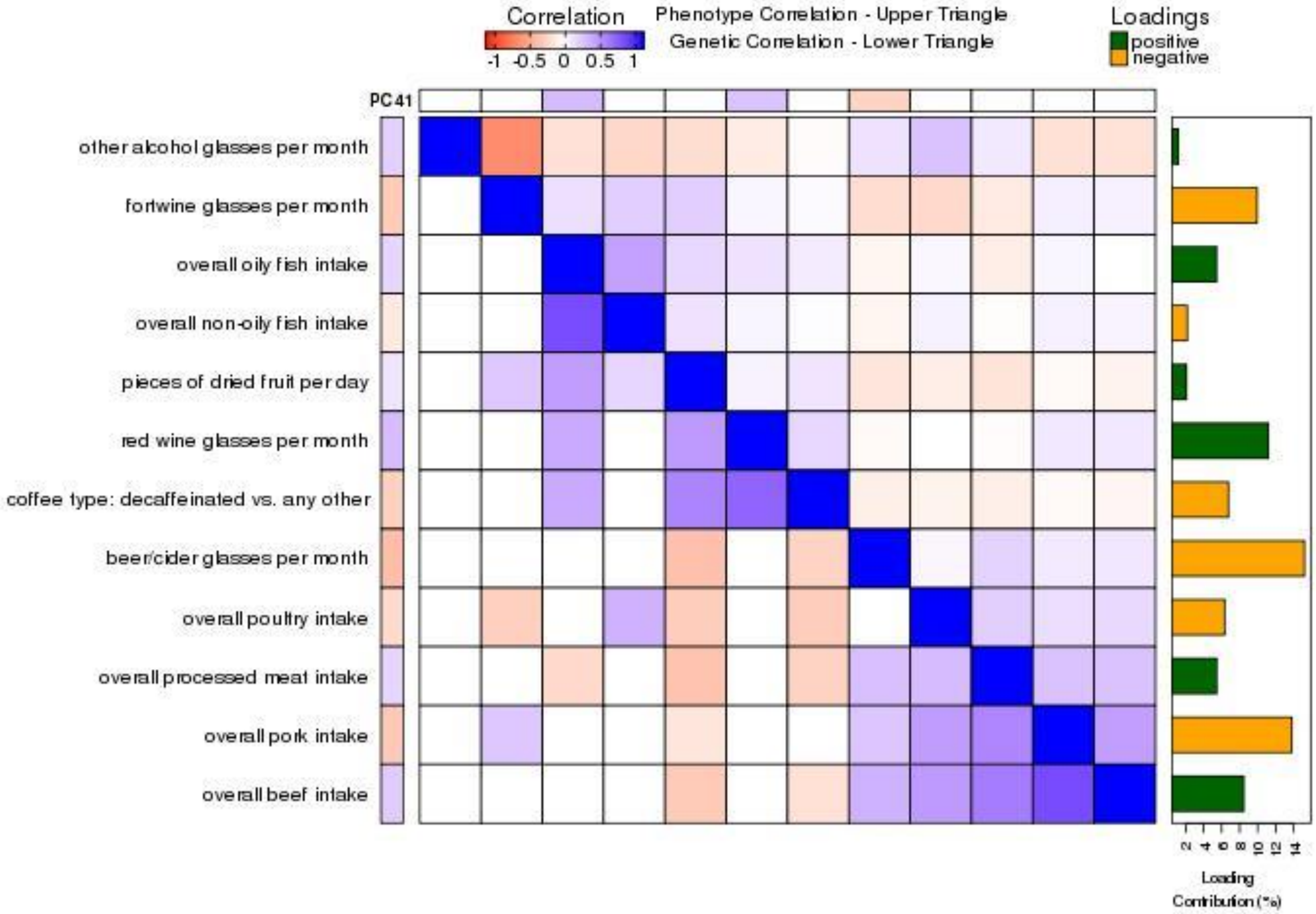
Correlation
-1 -0.5 0 0.5 1
Phenotype Correlation - Upper Triangle
Genetic Correlation - Lower Triangle
Loadings
positive
negative
PC41
other alcohol glasses per month
fortwine glasses per month
overall oily fish intake
overall non-oily fish intake
pieces of dried fruit per day
red wine glasses per month
coffee type: decaffeinated vs. any other
beer/cider glasses per month
overall poultry intake
overall processed meat intake
overall pork intake
overall beef intake
2 4 6 8 10 12 14
Loading
Contribution (%)

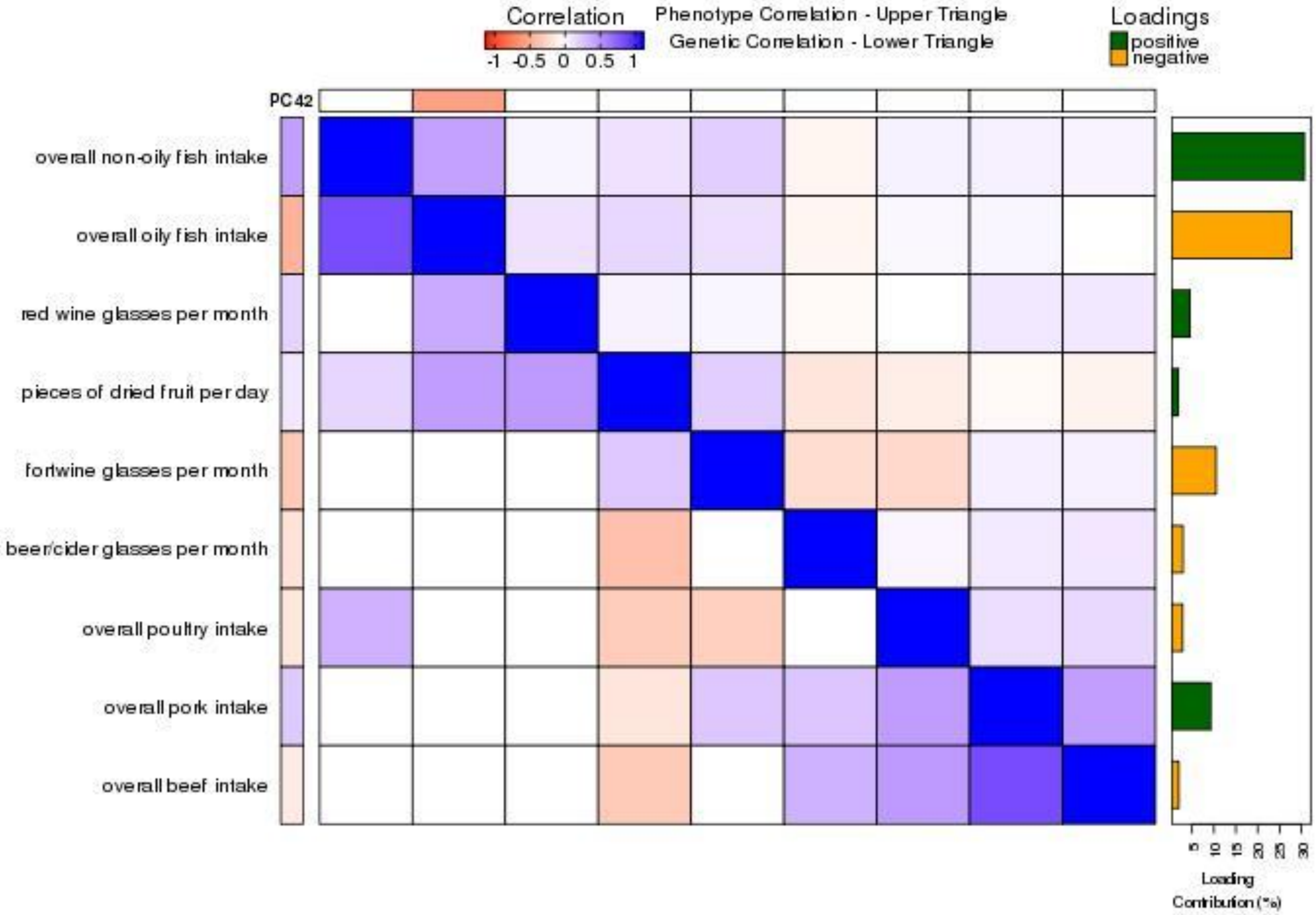
Correlation
-1 -0.5 0 0.5 1
Phenotype Correlation - Upper Triangle
Genetic Correlation - Lower Triangle
Loadings
positive
negative
PC42
overall non-oily fish intake
overall oily fish intake
red wine glasses per month
pieces of dried fruit per day
fortwine glasses per month
beer/cider glasses per month
overall poultry intake
overall pork intake
overall beef intake
5 10 15 20 25 30
Loading
Contribution (%)

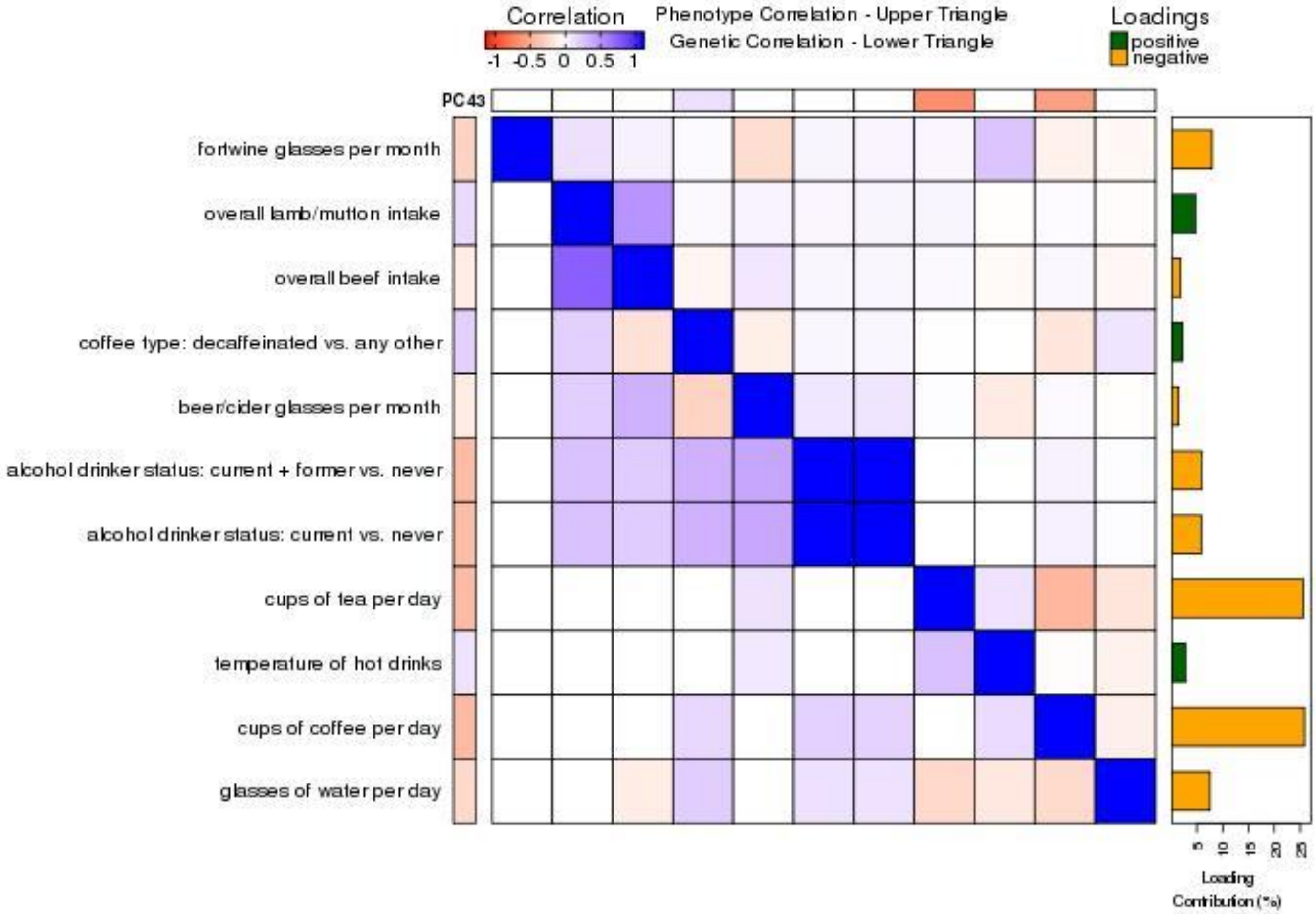
Correlation
-1 -0.5 0 0.5 1
Phenotype Correlation - Upper Triangle
Genetic Correlation - Lower Triangle
Loadings
positive
negative
PC43
fortwine glasses per month
overall lamb/mutton intake
overall beef intake
coffee type: decaffeinated vs. any other
beer/cider glasses per month
alcohol drinker status: current + former vs. never
alcohol drinker status: current vs. never
cups of tea per day
temperature of hot drinks
cups of coffee per day
glasses of water per day
5 10 15 20 25
Loading Contribution (%)

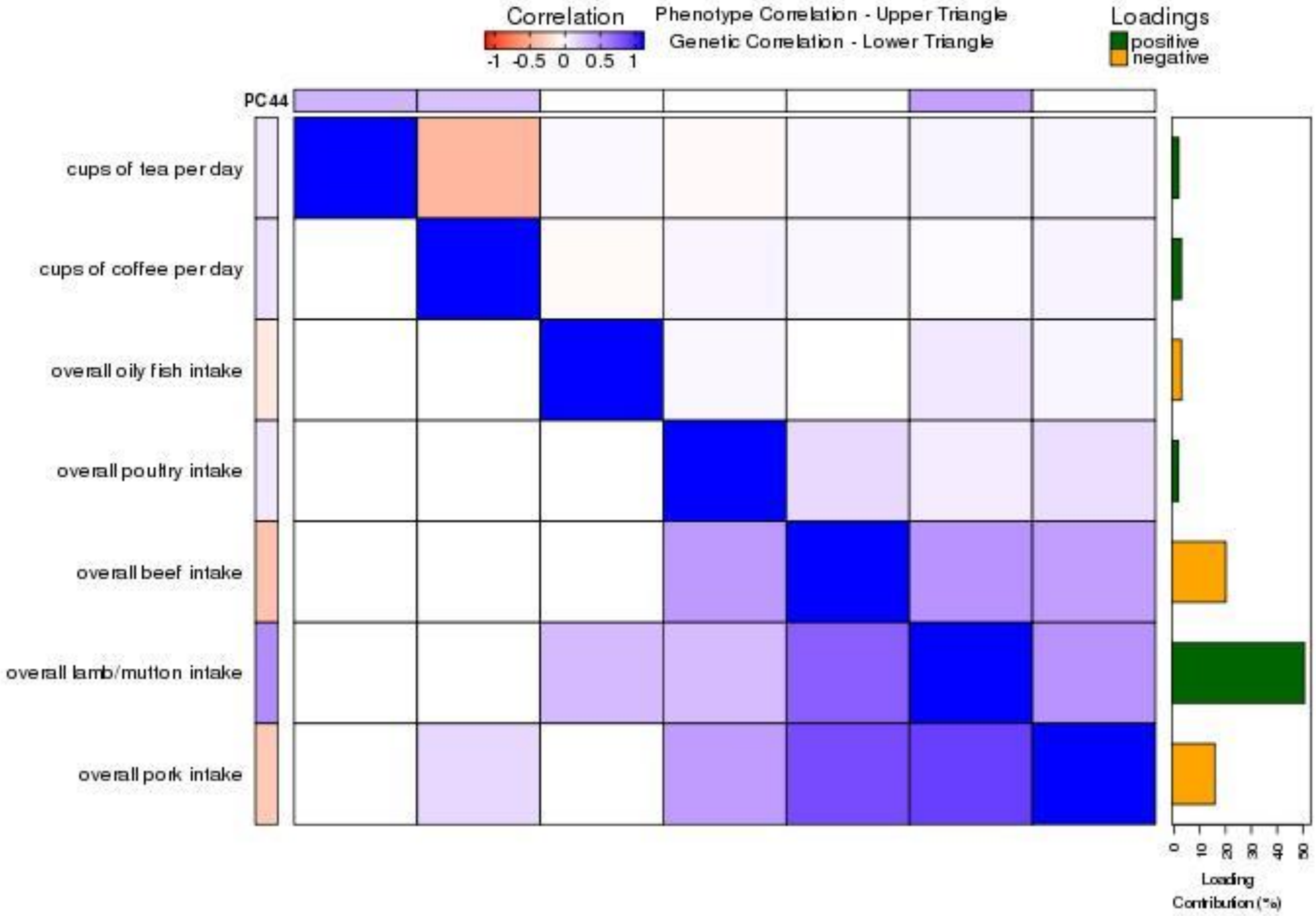
Correlation
-1 -0.5 0 0.5 1
Phenotype Correlation - Upper Triangle
Genetic Correlation - Lower Triangle
Loadings
positive
negative
PC44
cups of tea per day
cups of coffee per day
overall oily fish intake
overall poultry intake
overall beef intake
overall lamb/mutton intake
overall pork intake
0 10 20 30 40 50
Loading Contribution (%)

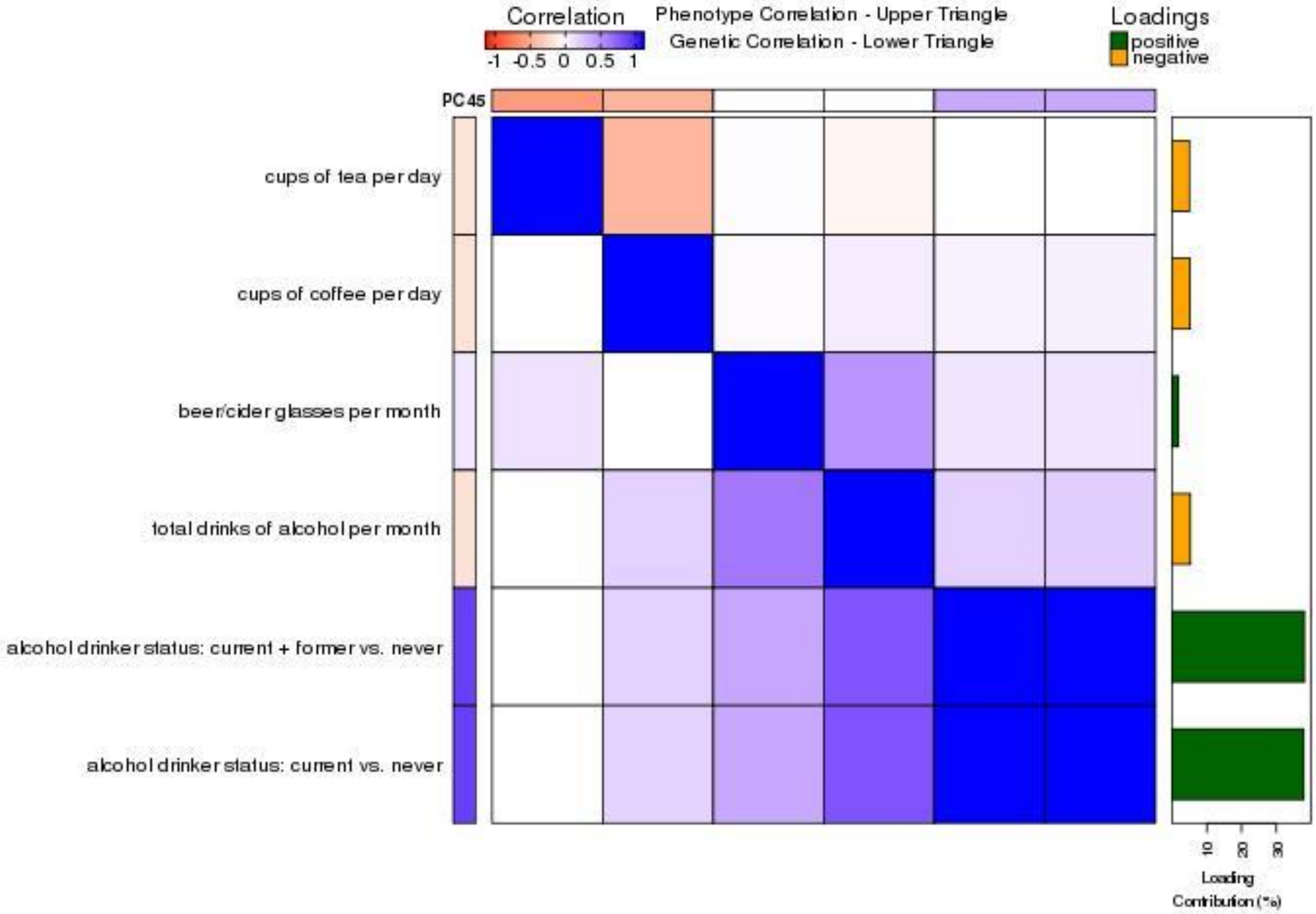
Correlation
-1 -0.5 0 0.5 1
Phenotype Correlation - Upper Triangle
Genetic Correlation - Lower Triangle
Loadings
positive
negative
PC45
cups of tea per day
cups of coffee per day
beer/cider glasses per month
total drinks of alcohol per month
alcohol drinker status: current + former vs. never
alcohol drinker status: current vs. never
10 20 30
Loading
Contribution (%)

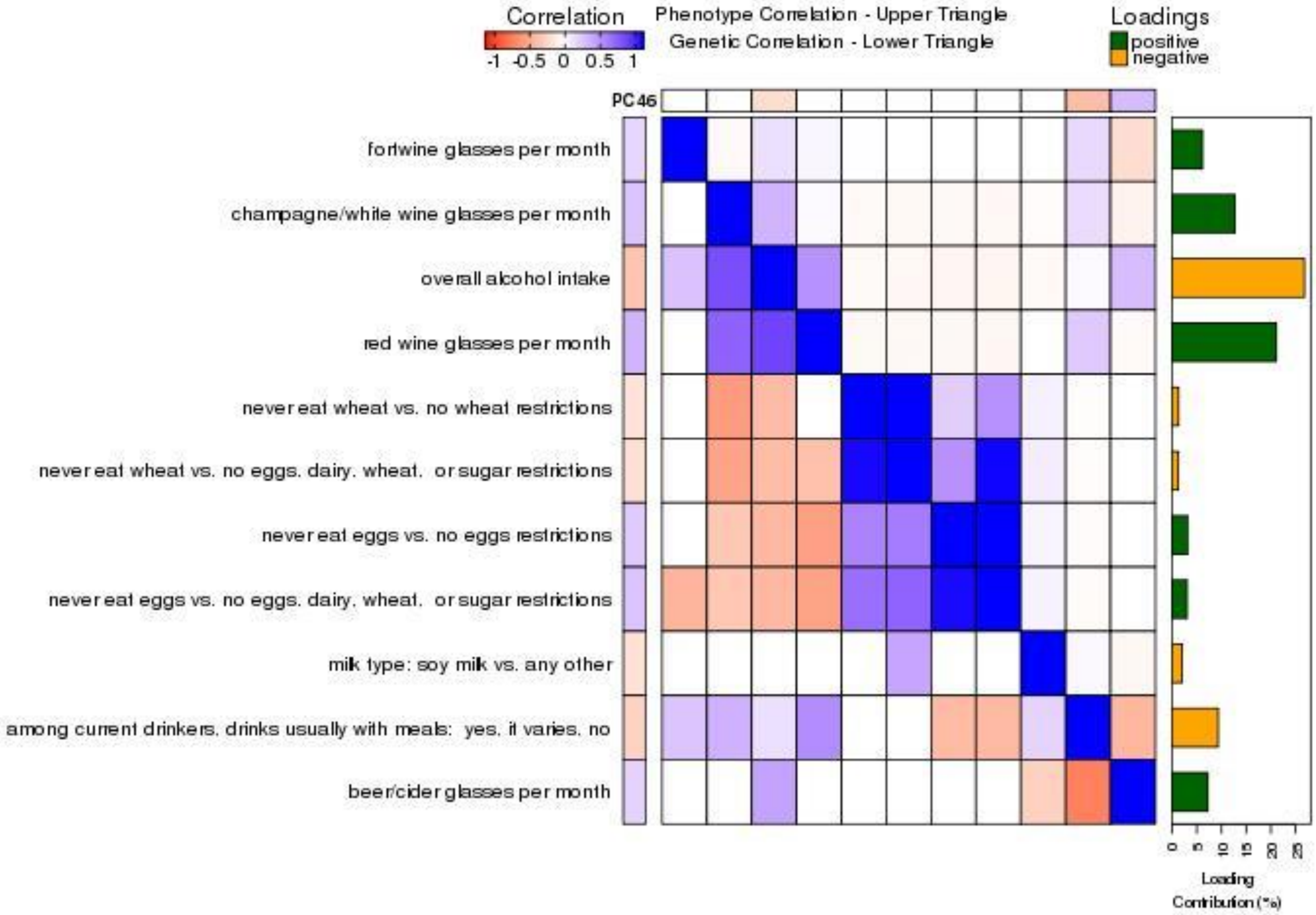
Correlation
-1 -0.5 0 0.5 1
Phenotype Correlation - Upper Triangle
Genetic Correlation - Lower Triangle
Loadings
positive
negative
PC46
fortwine glasses per month
champagne/white wine glasses per month
overall alcohol intake
red wine glasses per month
never eat wheat vs. no wheat restrictions
never eat wheat vs. no eggs, dairy, wheat, or sugar restrictions
never eat eggs vs. no eggs restrictions
never eat eggs vs. no eggs, dairy, wheat, or sugar restrictions
milk type: soy milk vs. any other
among current drinkers, drinks usually with meals: yes, it varies, no
beer/cider glasses per month
0 5 10 15 20 25
Loading
Contribution (%)

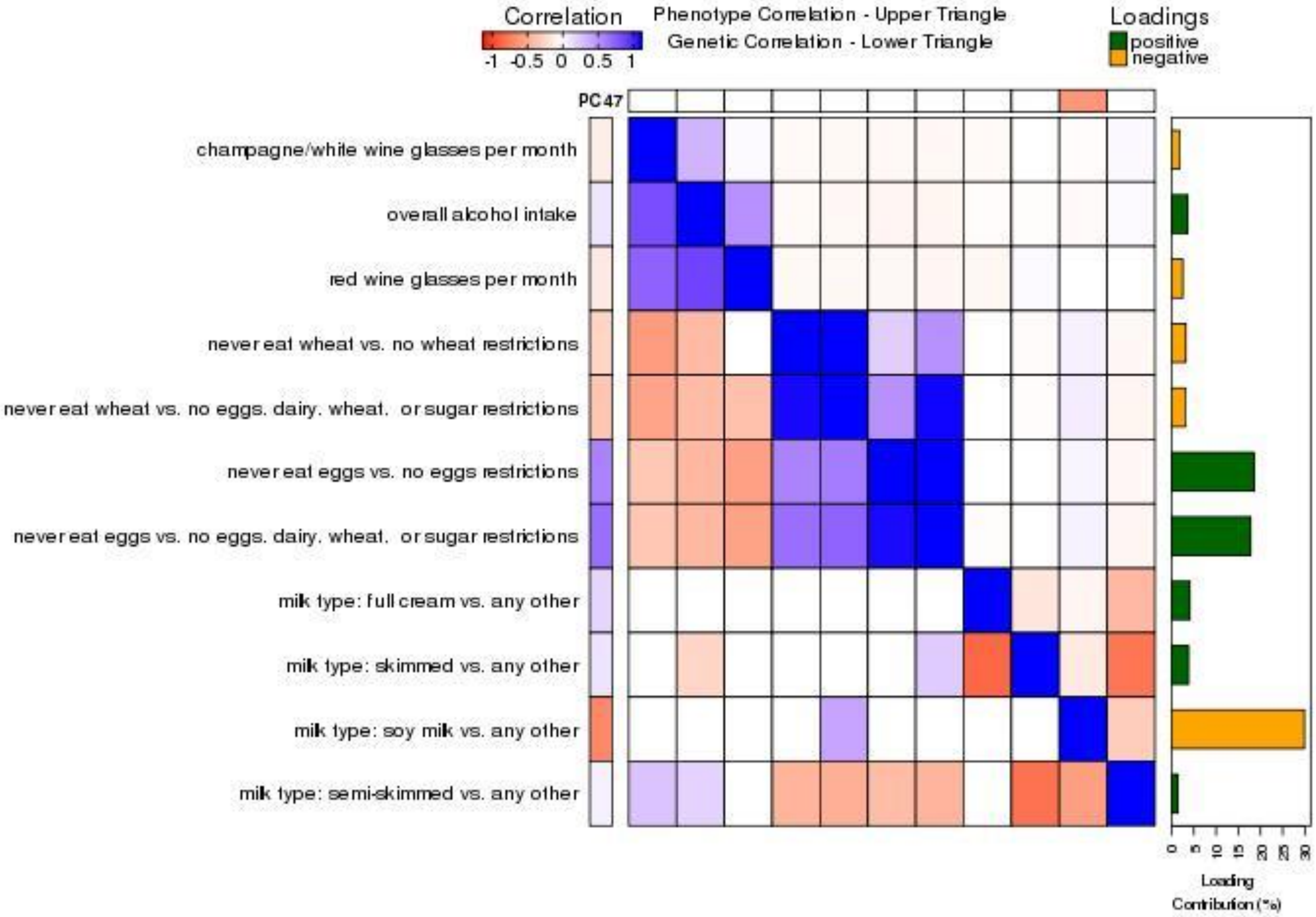
Correlation
-1 -0.5 0 0.5 1
Phenotype Correlation - Upper Triangle
Genetic Correlation - Lower Triangle
Loadings
positive
negative
PC47
champagne/white wine glasses per month
overall alcohol intake
red wine glasses per month
never eat wheat vs. no wheat restrictions
never eat wheat vs. no eggs, dairy, wheat, or sugar restrictions
never eat eggs vs. no eggs restrictions
never eat eggs vs. no eggs, dairy, wheat, or sugar restrictions
milk type: full cream vs. any other
milk type: skimmed vs. any other
milk type: soy milk vs. any other
milk type: semi-skimmed vs. any other
0 5 10 15 20 25 30
Loading
Contribution (%)

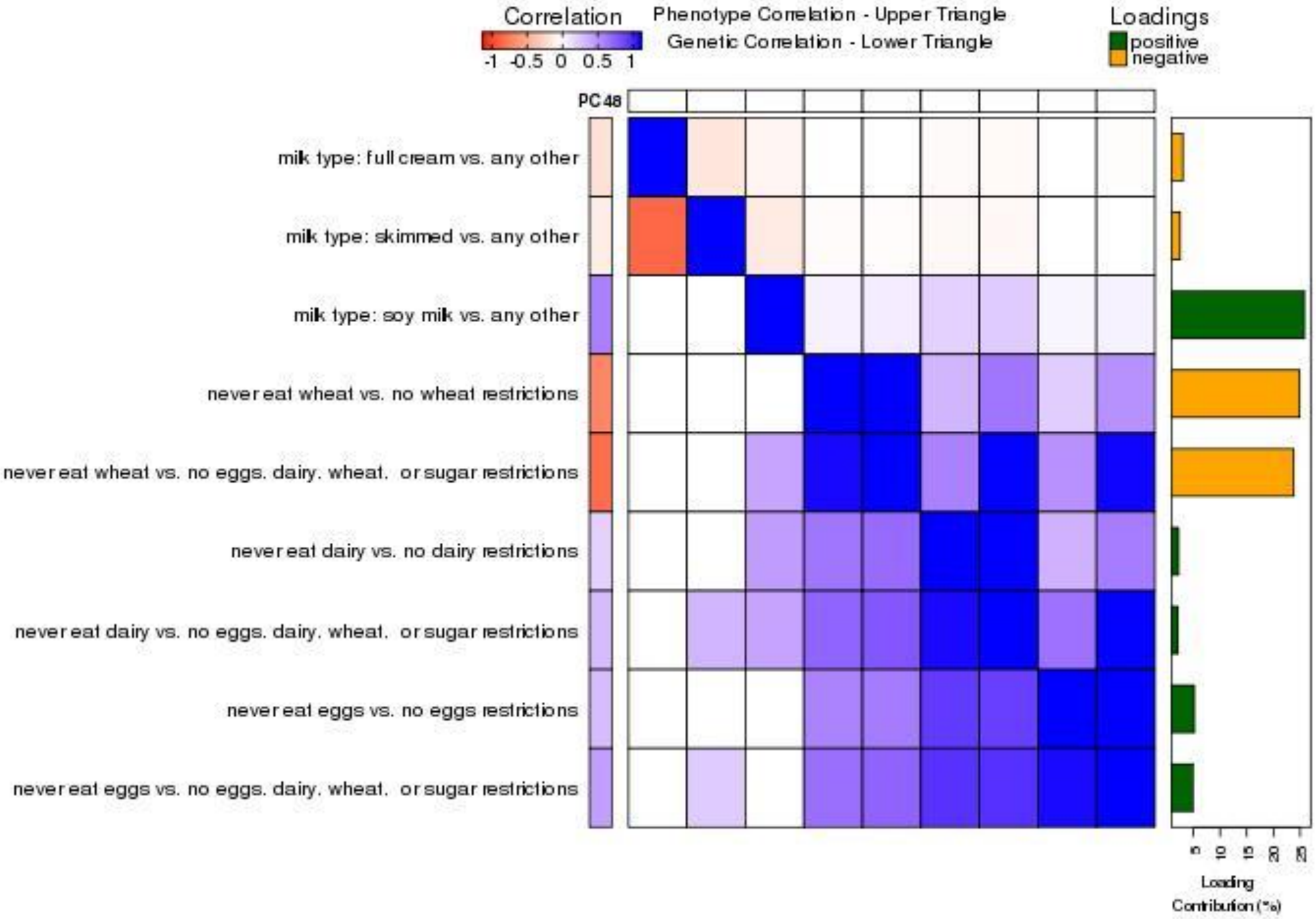
Correlation
-1 -0.5 0 0.5 1
Phenotype Correlation - Upper Triangle
Genetic Correlation - Lower Triangle
Loadings
positive
negative
PC48
milk type: full cream vs. any other
milk type: skimmed vs. any other
milk type: soy milk vs. any other
never eat wheat vs. no wheat restrictions
never eat wheat vs. no eggs, dairy, wheat, or sugar restrictions
never eat dairy vs. no dairy restrictions
never eat dairy vs. no eggs, dairy, wheat, or sugar restrictions
never eat eggs vs. no eggs restrictions
never eat eggs vs. no eggs, dairy, wheat, or sugar restrictions
5 10 15 20 25
Loading
Contribution (%)

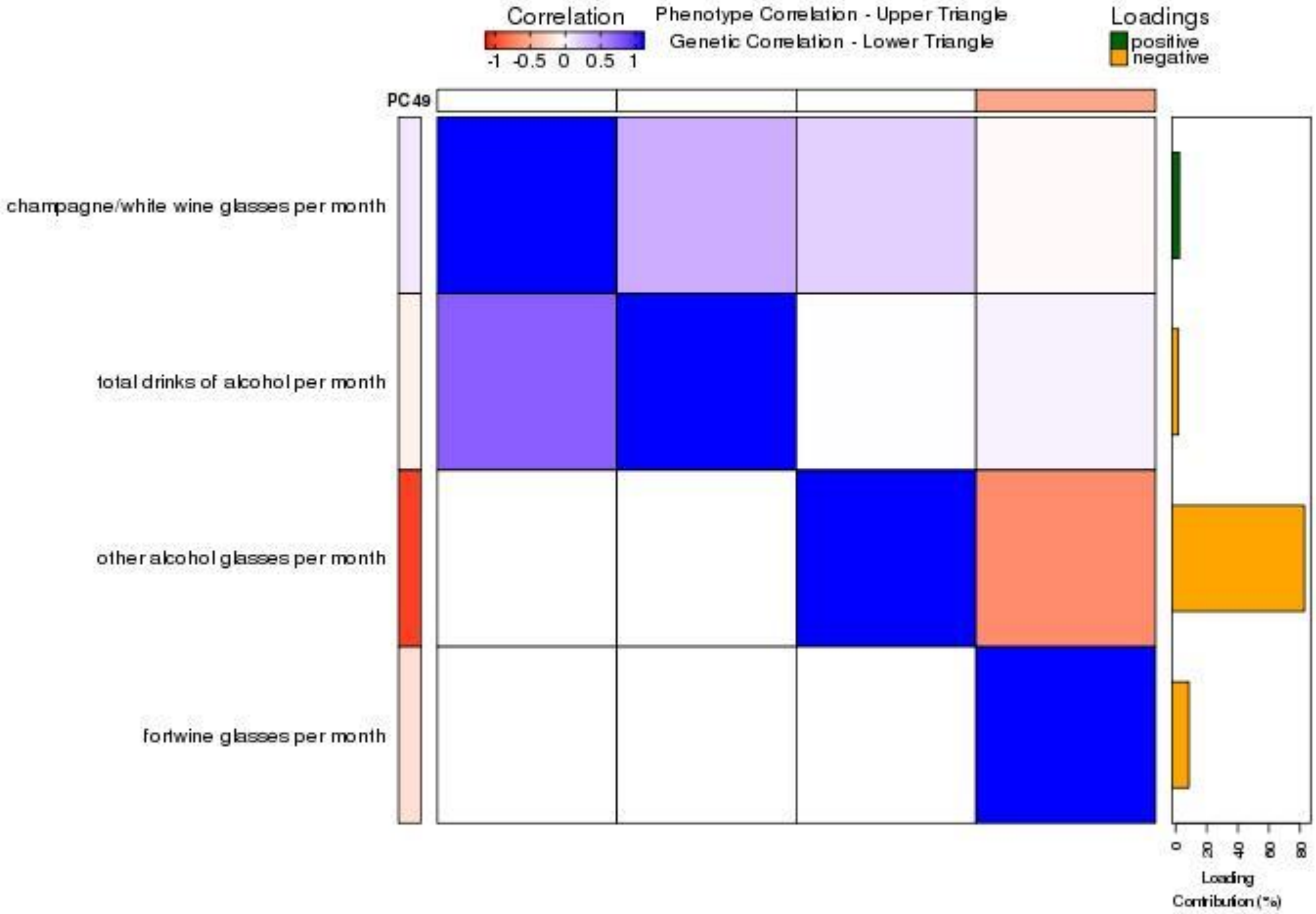

Correlation
-1 -0.5 0 0.5 1
Phenotype Correlation - Upper Triangle
Genetic Correlation - Lower Triangle
Loadings
positive
negative
PC49
champagne/white wine glasses per month
total drinks of alcohol per month
other alcohol glasses per month
fortwine glasses per month
0 20 40 60 80
Loading Contribution (%)

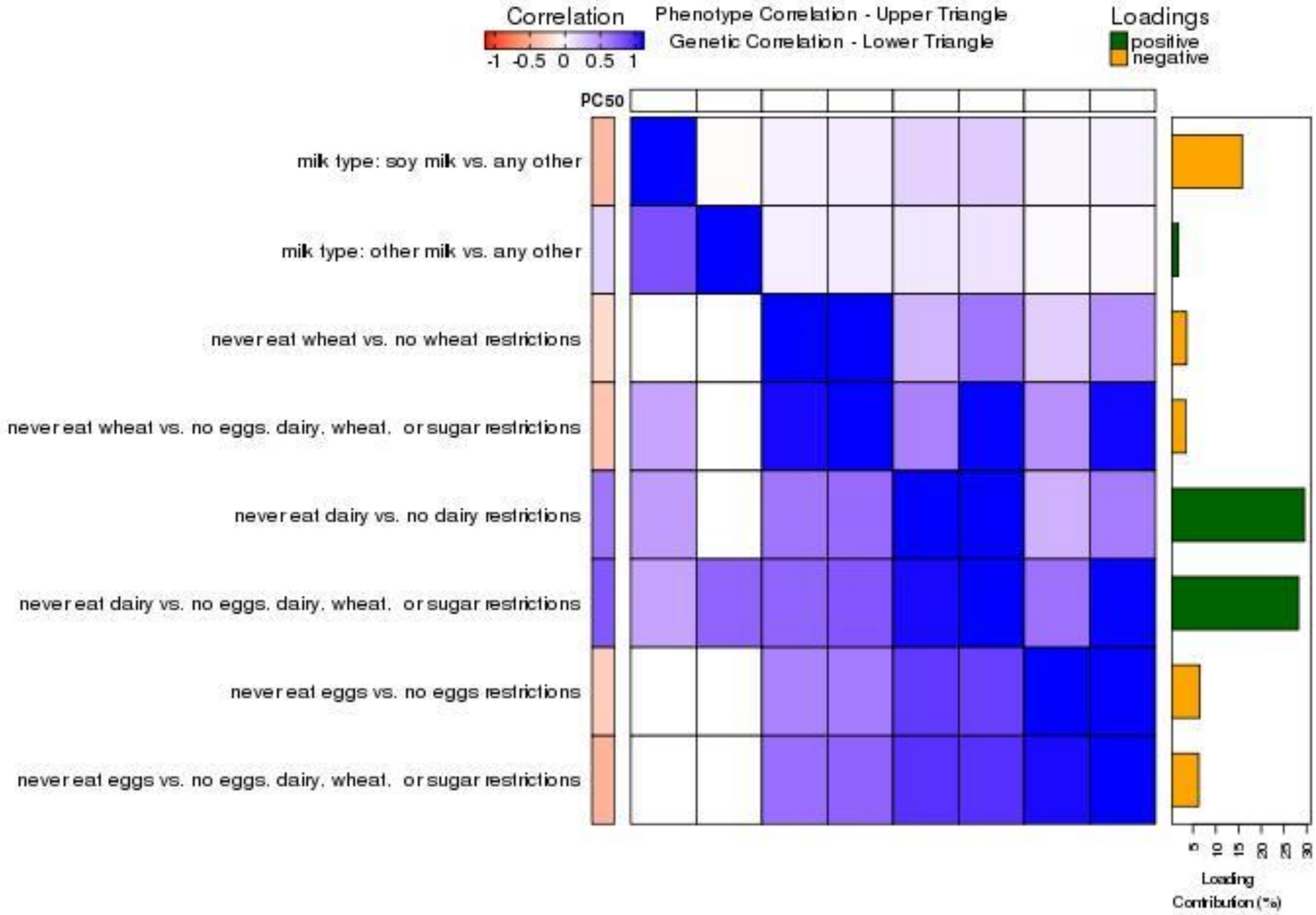

Correlation
-1 -0.5 0 0.5 1
Phenotype Correlation - Upper Triangle
Genetic Correlation - Lower Triangle
Loadings
positive
negative
PC50
milk type: soy milk vs. any other
milk type: other milk vs. any other
never eat wheat vs. no wheat restrictions
never eat wheat vs. no eggs, dairy, wheat, or sugar restrictions
never eat dairy vs. no dairy restrictions
never eat dairy vs. no eggs, dairy, wheat, or sugar restrictions
never eat eggs vs. no eggs restrictions
never eat eggs vs. no eggs, dairy, wheat, or sugar restrictions
5 10 15 20 25 30
Loading Contribution (%)

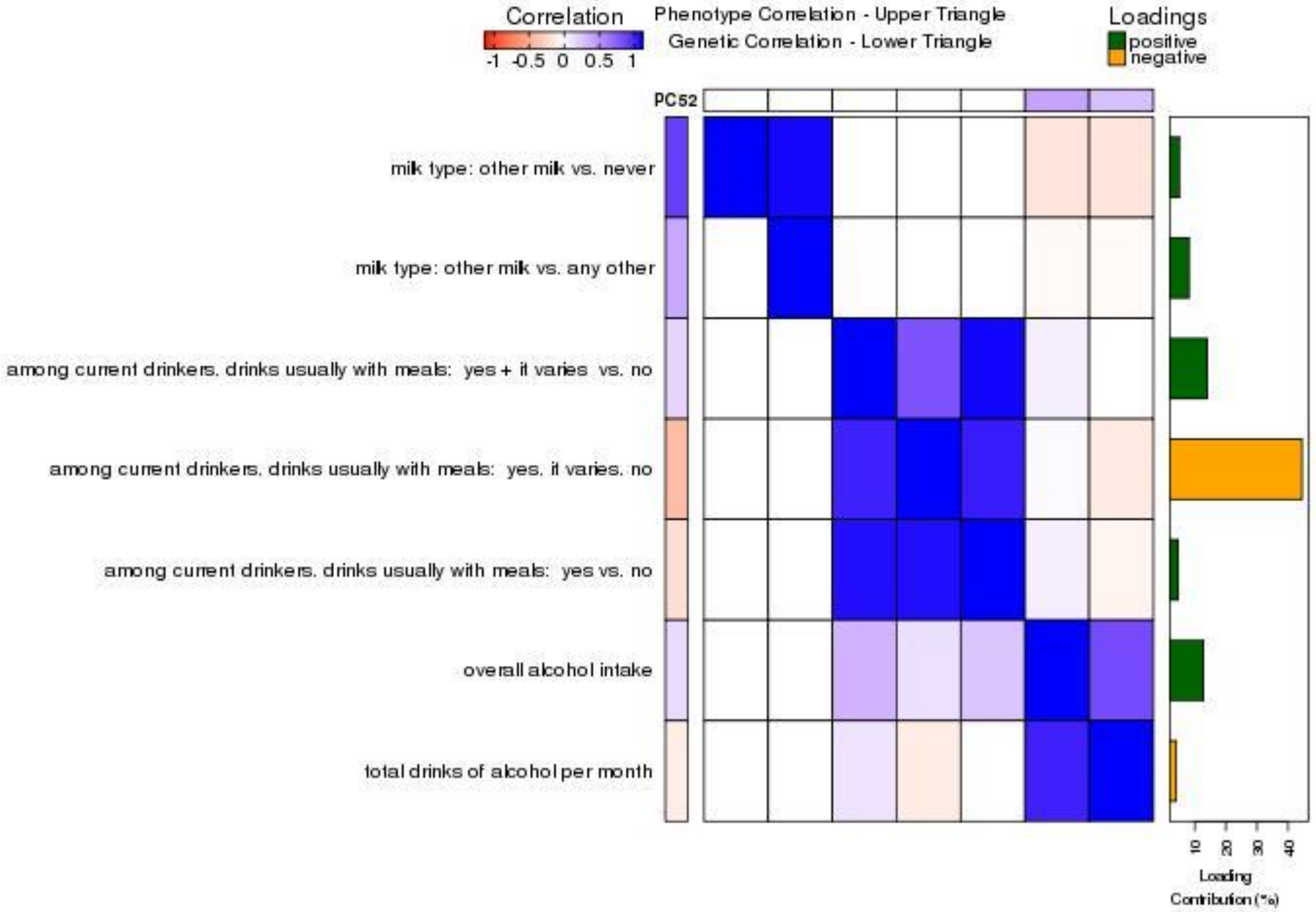

Correlation
-1 -0.5 0 0.5 1
Phenotype Correlation - Upper Triangle
Genetic Correlation - Lower Triangle
Loadings
positive
negative
PC52
milk type: other milk vs. never
milk type: other milk vs. any other
among current drinkers, drinks usually with meals: yes + it varies vs. no
among current drinkers, drinks usually with meals: yes, it varies, no
among current drinkers, drinks usually with meals: yes vs. no
overall alcohol intake
total drinks of alcohol per month
10
20
30
40
Loading Contribution (%)

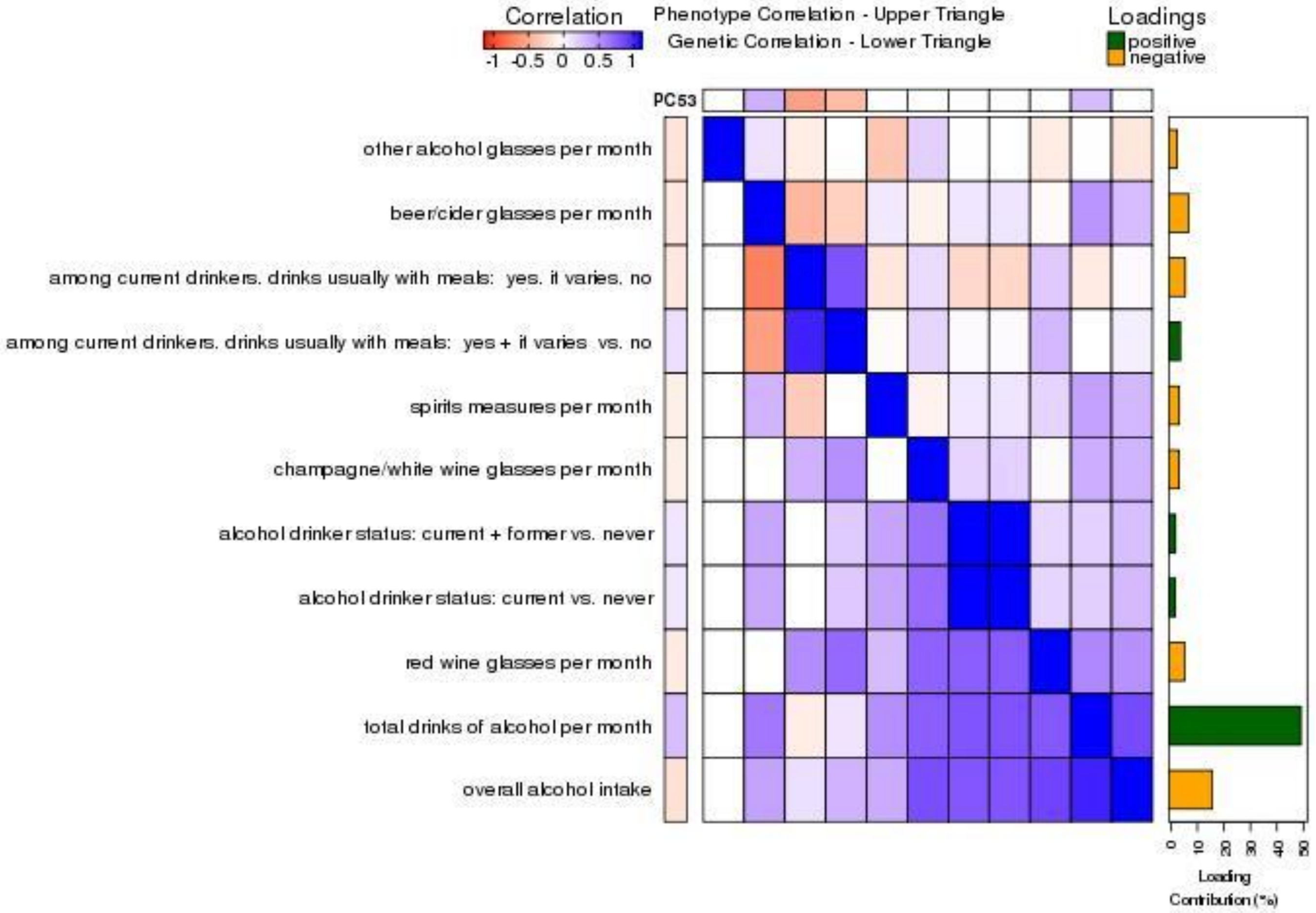

Correlation
-1 -0.5 0 0.5 1
Phenotype Correlation - Upper Triangle
Genetic Correlation - Lower Triangle
Loadings
positive
negative
PC53
other alcohol glasses per month
beer/cider glasses per month
among current drinkers, drinks usually with meals: yes, it varies, no
among current drinkers, drinks usually with meals: yes + it varies vs. no
spirits measures per month
champagne/white wine glasses per month
alcohol drinker status: current + former vs. never
alcohol drinker status: current vs. never
red wine glasses per month
total drinks of alcohol per month
overall alcohol intake
0
10
20
30
40
50
Loading Contribution (%)

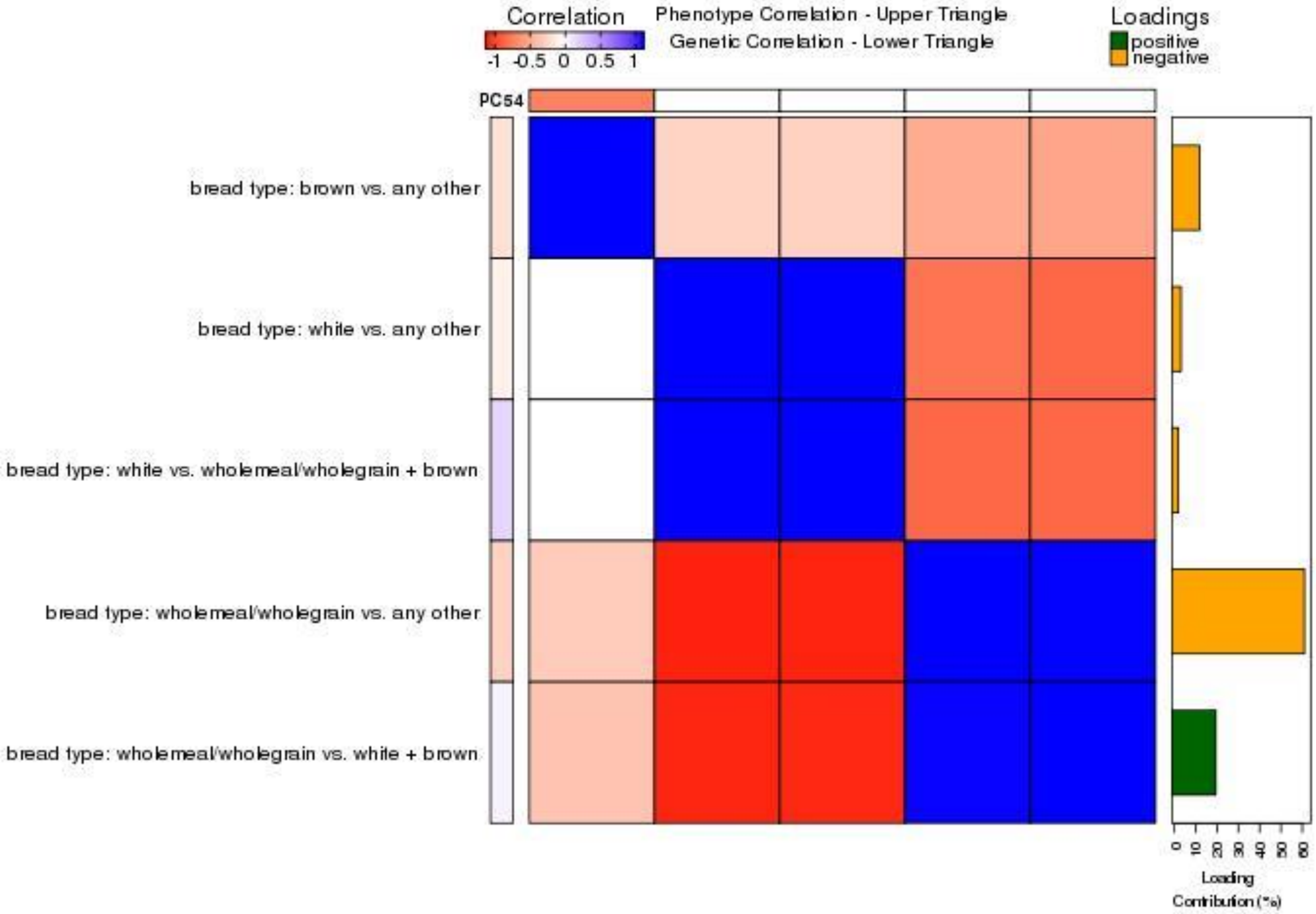
Correlation
-1 -0.5 0 0.5 1
Phenotype Correlation - Upper Triangle
Genetic Correlation - Lower Triangle
Loadings
positive
negative
PC54
bread type: brown vs. any other
bread type: white vs. any other
bread type: white vs. wholemeal/wholegrain + brown
bread type: wholemeal/wholegrain vs. any other
bread type: wholemeal/wholegrain vs. white + brown
0 10 20 30 40 50 60
Loading Contribution (%)

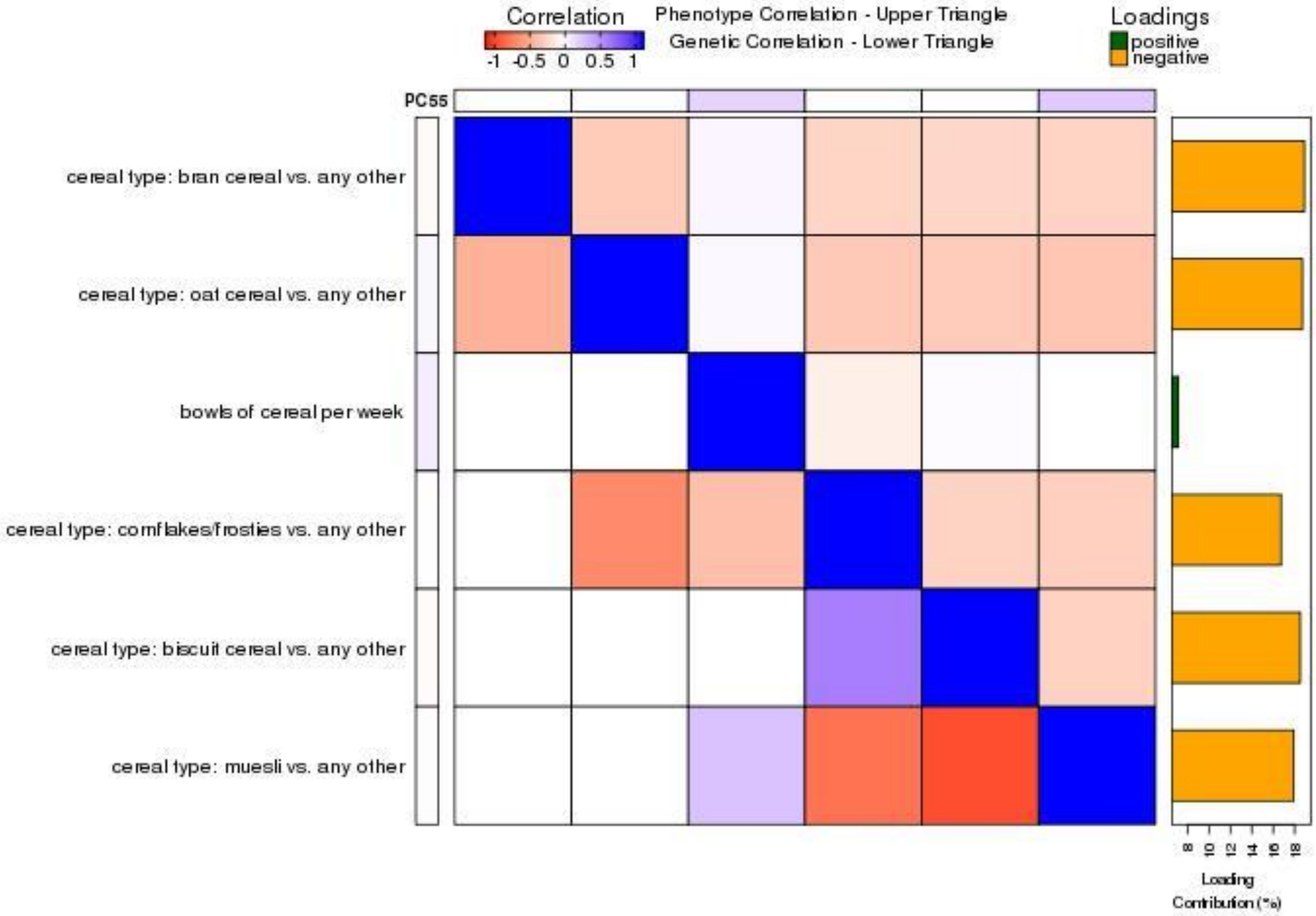
Correlation
-1 -0.5 0 0.5 1
Phenotype Correlation - Upper Triangle
Genetic Correlation - Lower Triangle
Loadings
positive
negative
PC55
cereal type: bran cereal vs. any other
cereal type: oat cereal vs. any other
bowls of cereal per week
cereal type: cornflakes/frosties vs. any other
cereal type: biscuit cereal vs. any other
cereal type: muesli vs. any other
8 10 12 14 16 18
Loading Contribution (%)

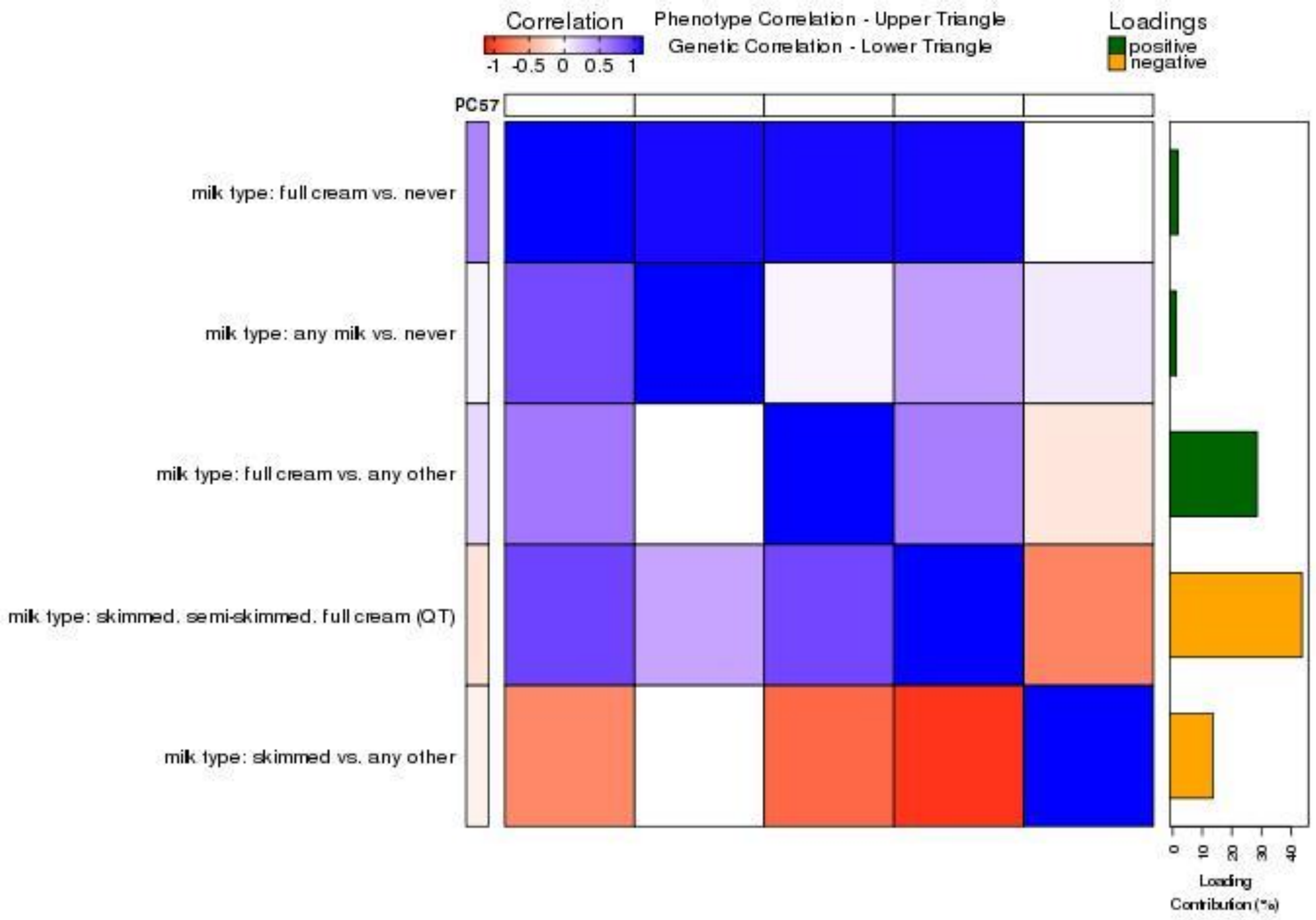

Correlation
-1 -0.5 0 0.5 1
Phenotype Correlation - Upper Triangle
Genetic Correlation - Lower Triangle
Loadings
positive
negative
PC57
milk type: full cream vs. never
milk type: any milk vs. never
milk type: full cream vs. any other
milk type: skimmed, semi-skimmed, full cream (QT)
milk type: skimmed vs. any other
0 10 20 30 40
Loading
Contribution (%)

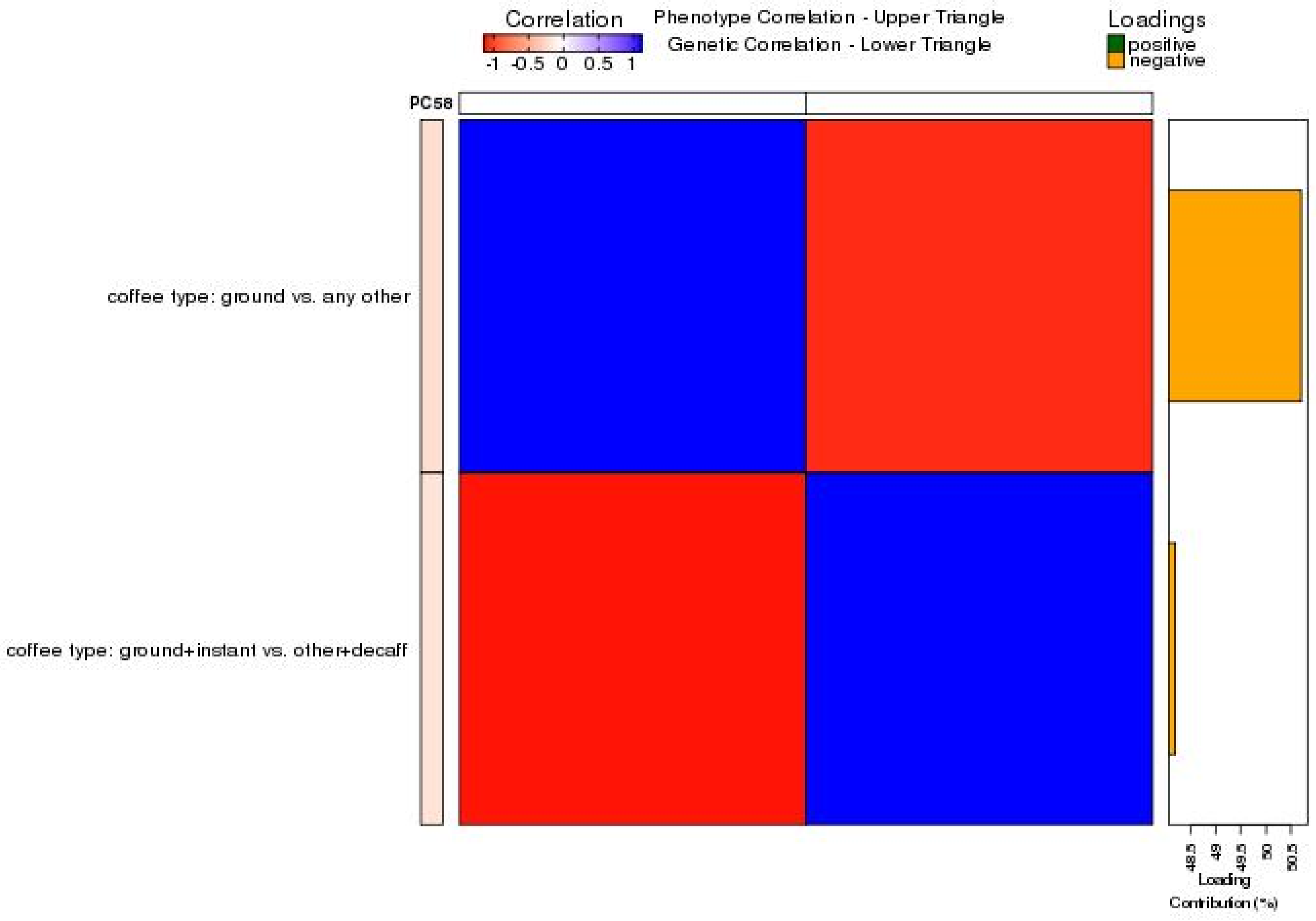

Correlation
-1 -0.5 0 0.5 1
Phenotype Correlation - Upper Triangle
Genetic Correlation - Lower Triangle
Loadings
positive
negative
PC58
coffee type: ground vs. any other
coffee type: ground+instant vs. other+decaff
48.5 49 49.5 50 50.5
Loading
Contribution (%)

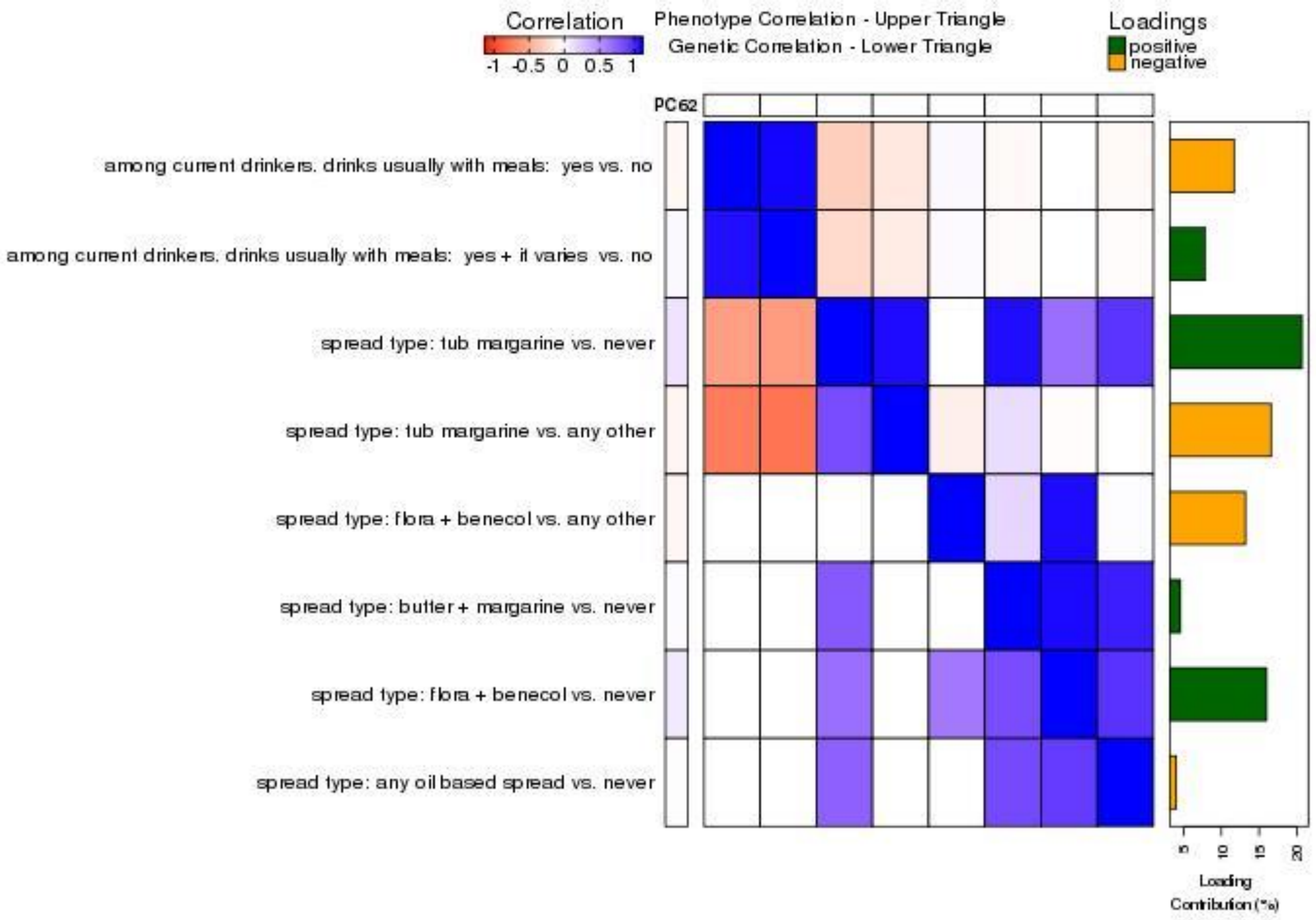
Correlation
-1 -0.5 0 0.5 1
Phenotype Correlation - Upper Triangle
Genetic Correlation - Lower Triangle
Loadings
positive
negative
PC62
among current drinkers. drinks usually with meals: yes vs. no
among current drinkers. drinks usually with meals: yes + it varies vs. no
spread type: tub margarine vs. never
spread type: tub margarine vs. any other
spread type: flora + benecol vs. any other
spread type: butter + margarine vs. never
spread type: flora + benecol vs. never
spread type: any oil based spread vs. never
5 10 15 20
Loading
Contribution (%)

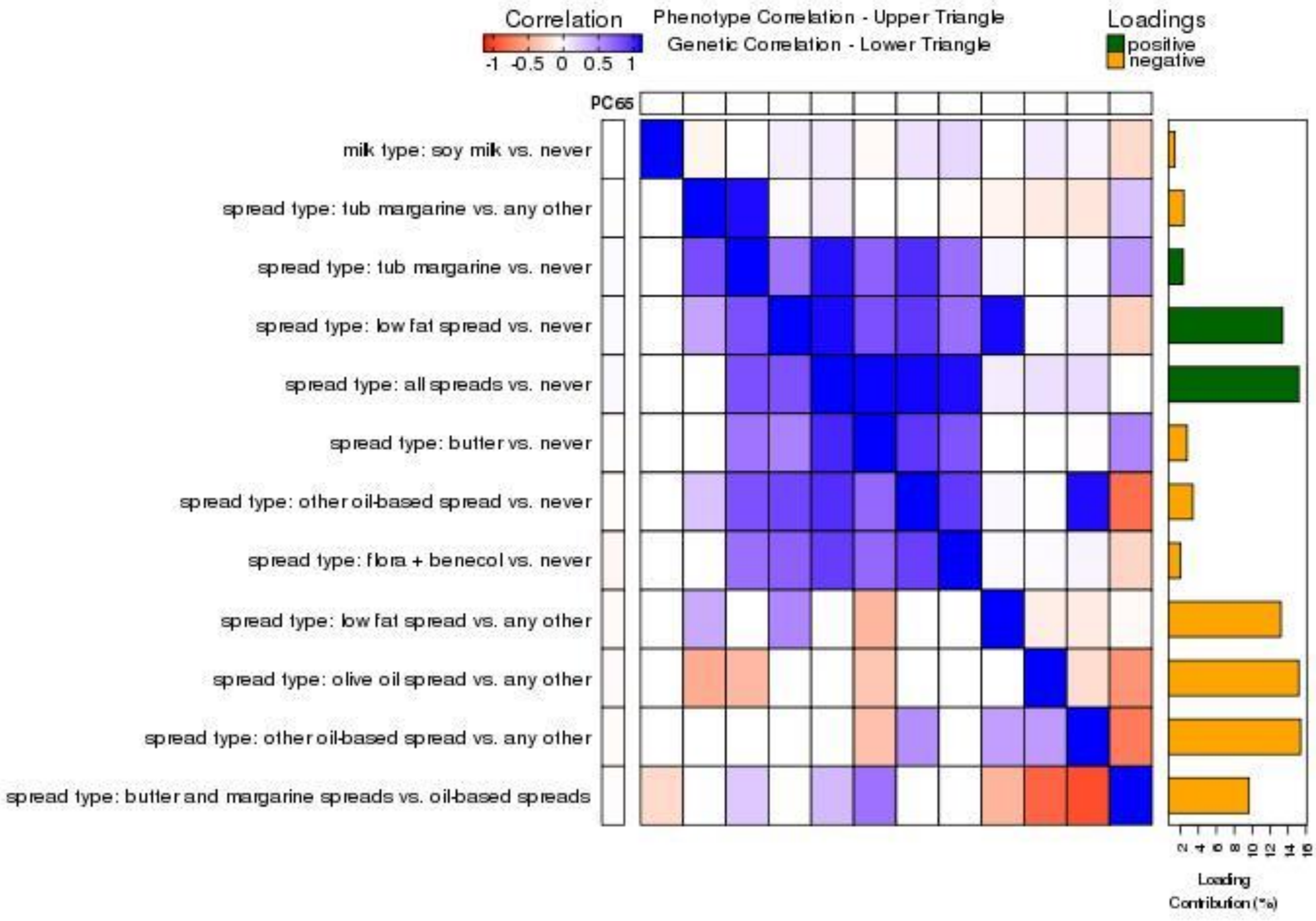
Correlation
-1 -0.5 0 0.5 1
Phenotype Correlation - Upper Triangle
Genetic Correlation - Lower Triangle
Loadings
positive
negative
PC65
milk type: soy milk vs. never
spread type: tub margarine vs. any other
spread type: tub margarine vs. never
spread type: low fat spread vs. never
spread type: all spreads vs. never
spread type: butter vs. never
spread type: other oil-based spread vs. never
spread type: flora + benecol vs. never
spread type: low fat spread vs. any other
spread type: olive oil spread vs. any other
spread type: other oil-based spread vs. any other
spread type: butter and margarine spreads vs. oil-based spreads
2 4 6 8 10 12 14 16
Loading
Contribution (%)

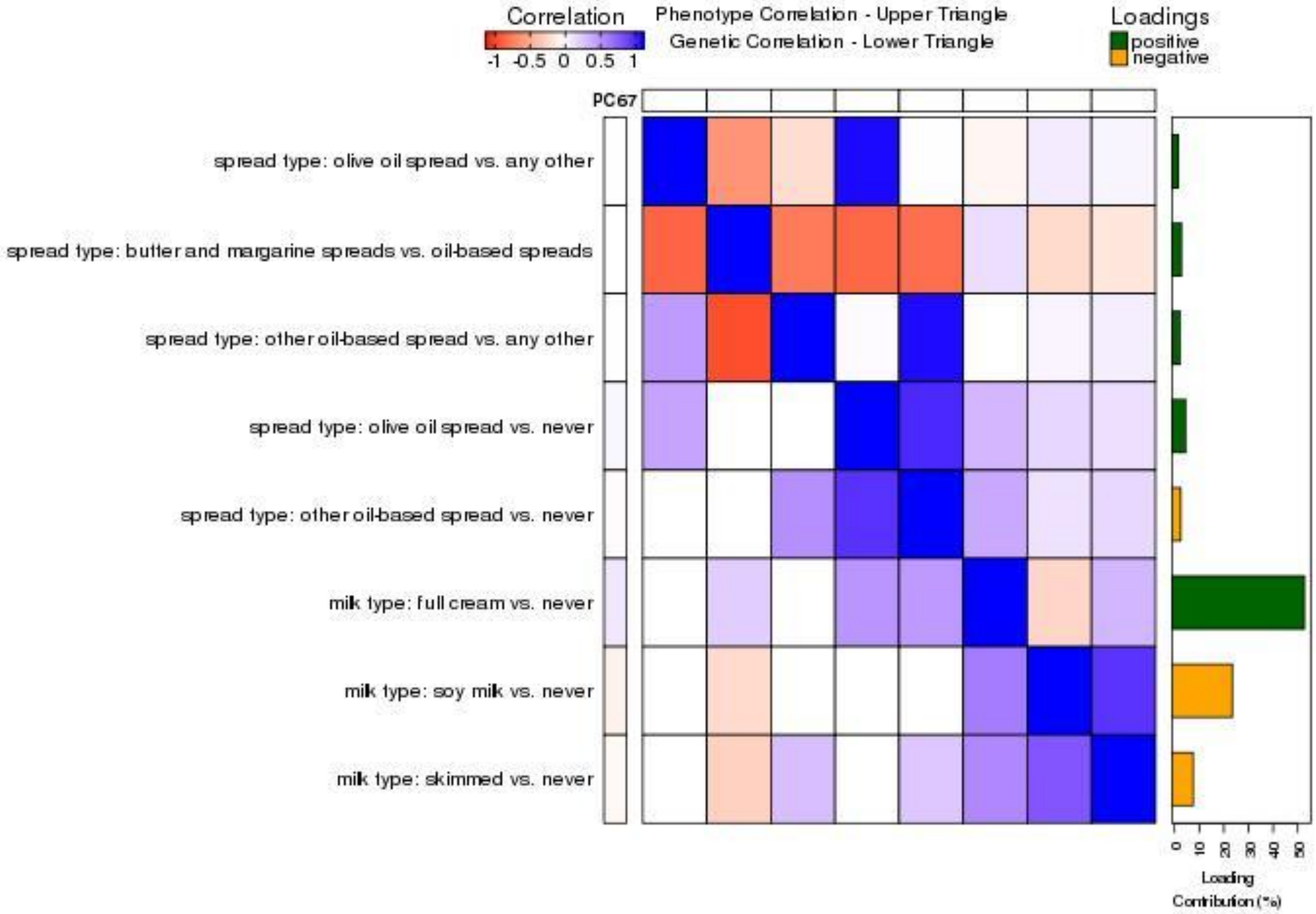
Correlation
-1 -0.5 0 0.5 1
Phenotype Correlation - Upper Triangle
Genetic Correlation - Lower Triangle
Loadings
positive
negative
PC67
spread type: olive oil spread vs. any other
spread type: butter and margarine spreads vs. oil-based spreads
spread type: other oil-based spread vs. any other
spread type: olive oil spread vs. never
spread type: other oil-based spread vs. never
milk type: full cream vs. never
milk type: soy milk vs. never
milk type: skimmed vs. never
0 10 20 30 40 50
Loading
Contribution (%)

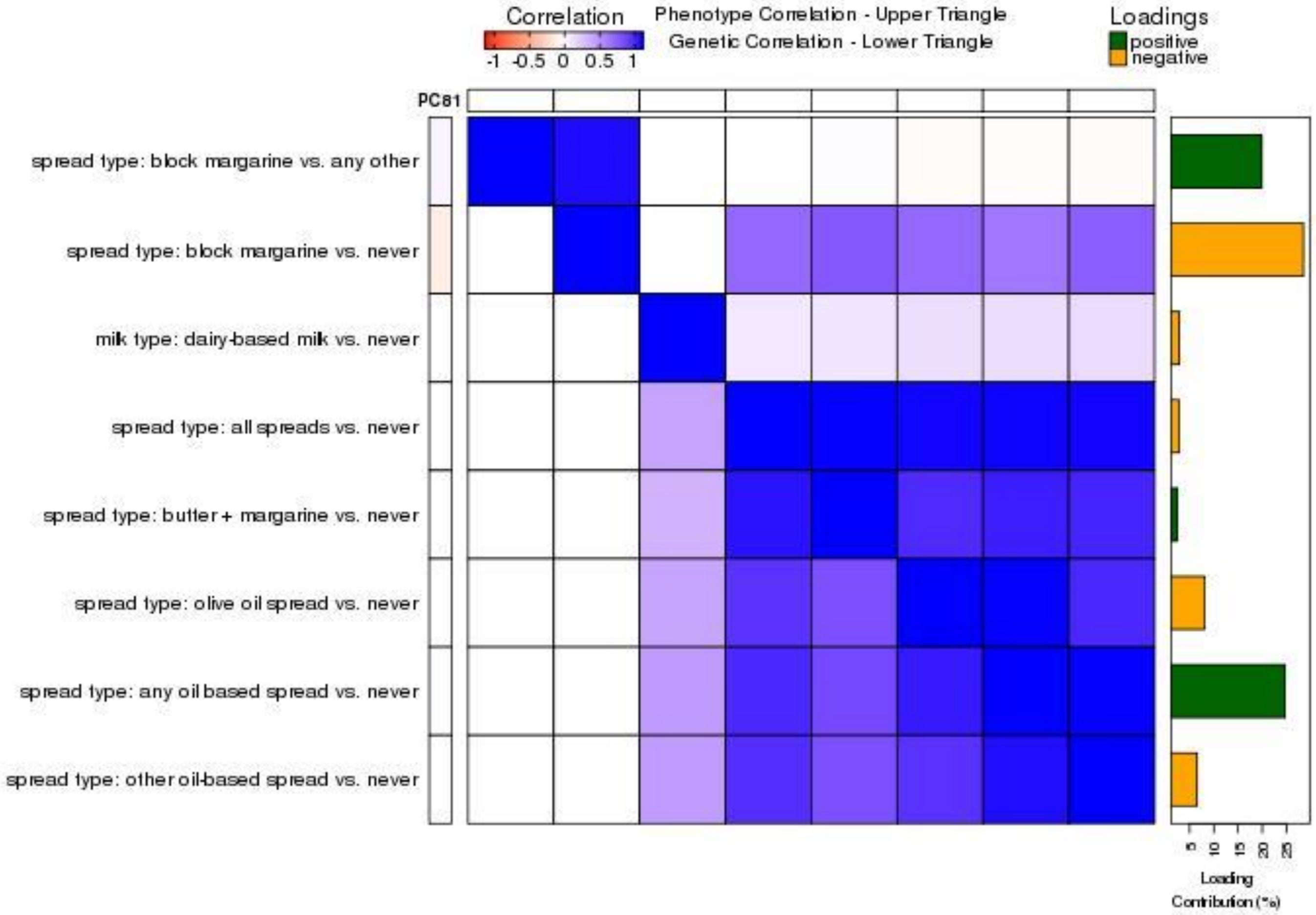
Correlation
-1 -0.5 0 0.5 1
Phenotype Correlation - Upper Triangle
Genetic Correlation - Lower Triangle
Loadings
positive
negative
PC81
spread type: block margarine vs. any other
spread type: block margarine vs. never
milk type: dairy-based milk vs. never
spread type: all spreads vs. never
spread type: butter + margarine vs. never
spread type: olive oil spread vs. never
spread type: any oil based spread vs. never
spread type: other oil-based spread vs. never
5 10 15 20 25
Loading
Contribution (%)

Supplementary Figure 5: Relationship between SNP heritability and GWAS success. A) Scatter plots of number of genome-wide significant loci or B) variance explained by genome-wide significant SNPs vs. SNP heritability for phenotypically independent DP-PCs, with points colored by the largest effect size and sized by the smallest P-value of their significant index SNPs. C) Scatter plots of the number of genome-wide significant loci or D) variance explained by genome-wide significant SNPs vs. SNP heritability for all 143 significantly heritable traits, with points colored by sample size.

a

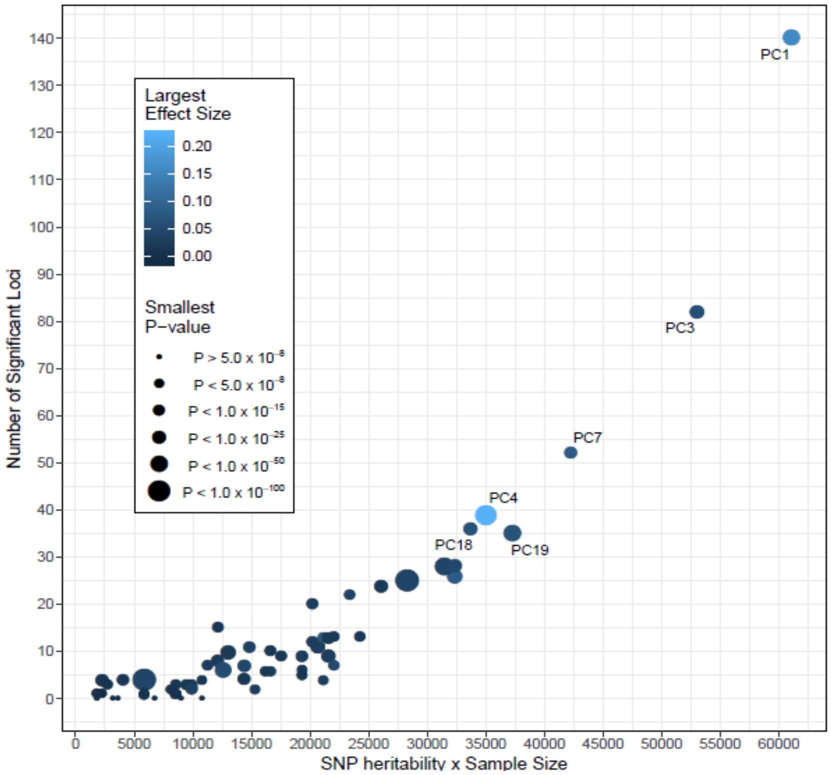

b

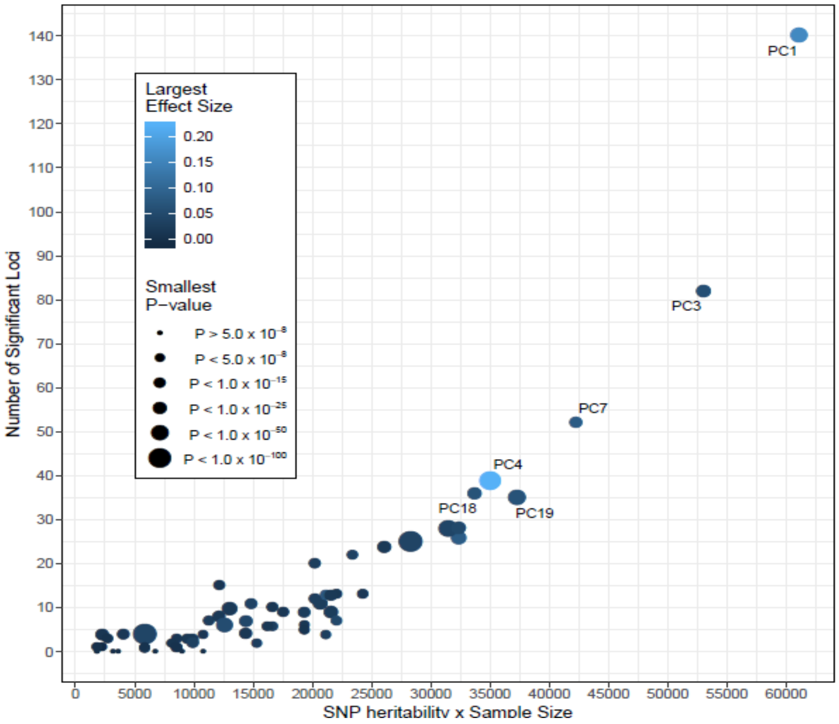

c

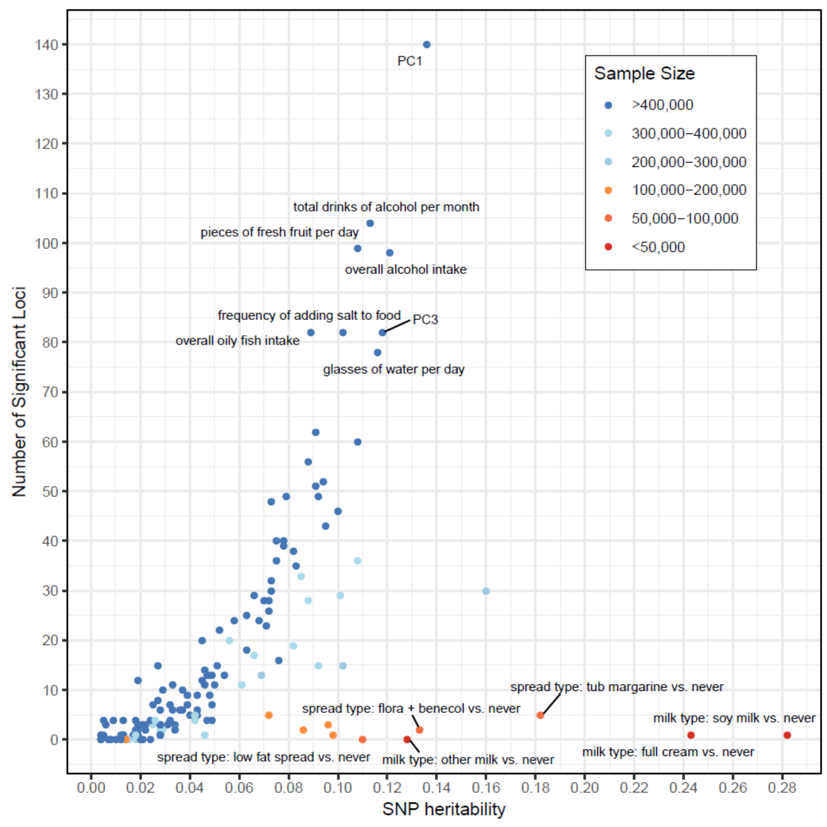

Sample Size
>400,000
300,000−400,000
200,000−300,000
100,000−200,000
50,000−100,000
<50,000
PC1
total drinks of alcohol per month
pieces of fresh fruit per day
overall alcohol intake
frequency of adding salt to food
PC3
overall oily fish intake
glasses of water per day
spread type: tub margarine vs. never
spread type: flora + benecol vs. never
milk type: soy milk vs. never
milk type: full cream vs. never
spread type: low fat spread vs. never
milk type: other milk vs. never
Number of Significant Loci
SNP heritability

d

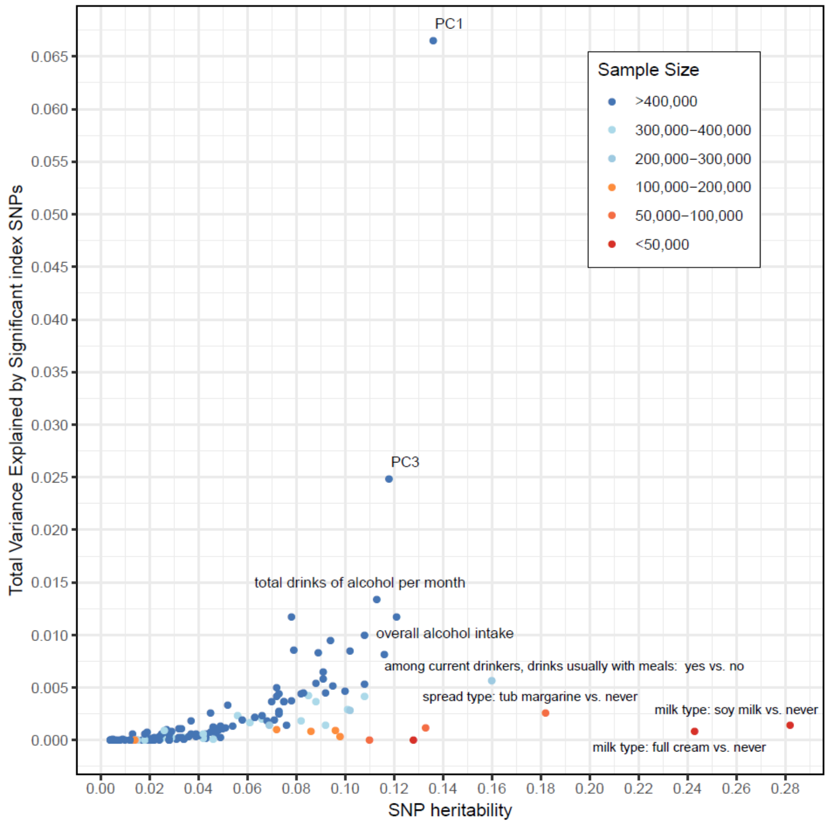

Sample Size
>400,000
300,000−400,000
200,000−300,000
100,000−200,000
50,000−100,000
<50,000
PC1
PC3
total drinks of alcohol per month
overall alcohol intake
among current drinkers, drinks usually with meals: yes vs. no
spread type: tub margarine vs. never
milk type: soy milk vs. never
milk type: full cream vs. never
Total Variance Explained by Significant index SNPs
SNP heritability

Supplementary Figure 6: Association of lead SNP P-values across all dietary habits with P<0.05. Traits in which the lead SNP reached genome-wide significance are colored in red. A) rs10249294 on chr7q35 is most strongly associated with "pieces of fresh fruit per day" and reaches genome-wide significance for 10 additional dietary habits correlated with "pieces of fresh fruit per day". B) rs34162196 reaches genome-wide significance for "pieces of fresh fruit per day" and "pieces of dried fruit per day" only. C) rs1229984 on chr4 in the *ADH1B* gene is most strongly associated with "total drinks of alcohol per month" and reaches genome-wide significance for 22 additional dietary habits.

a

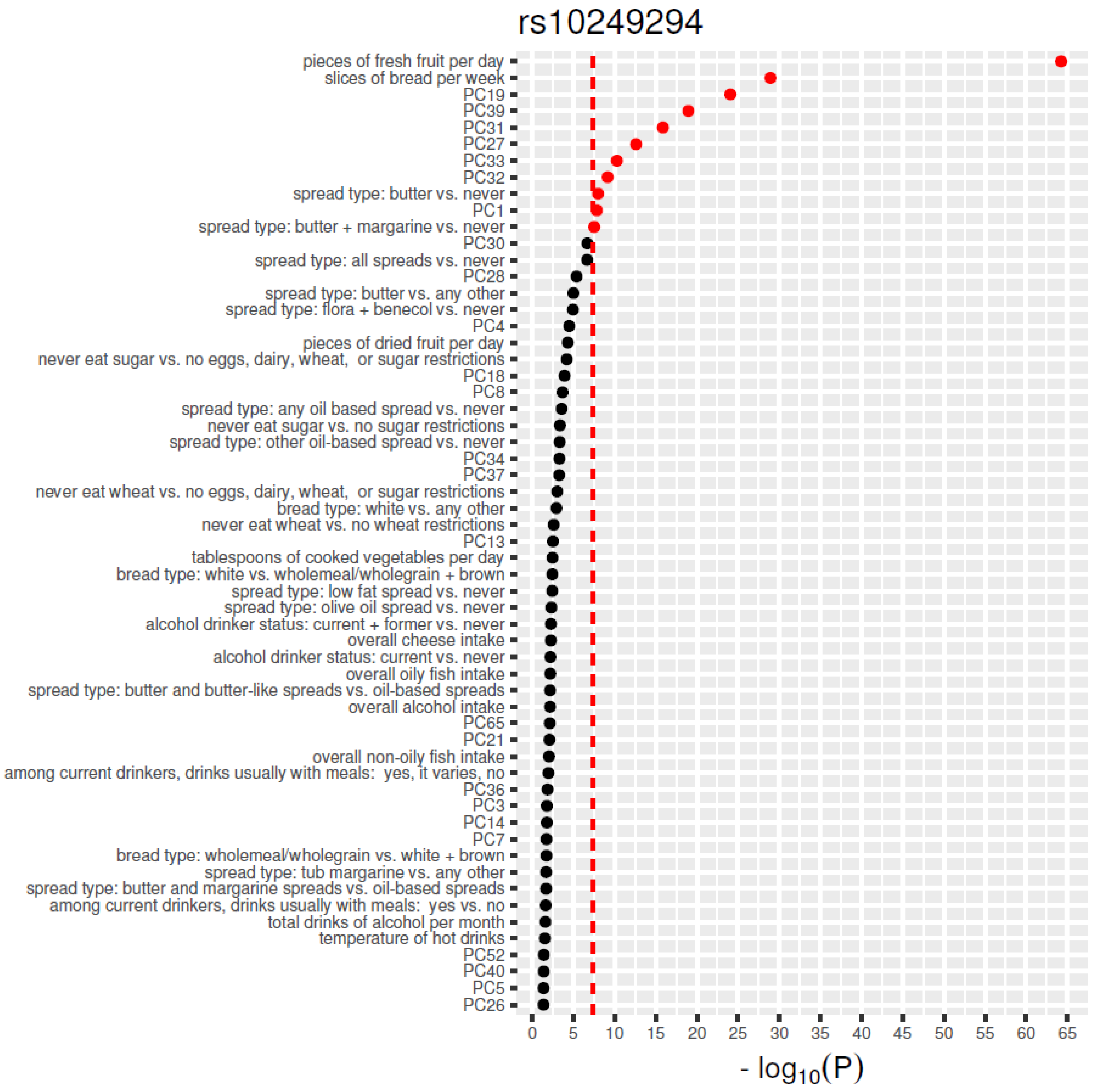

b

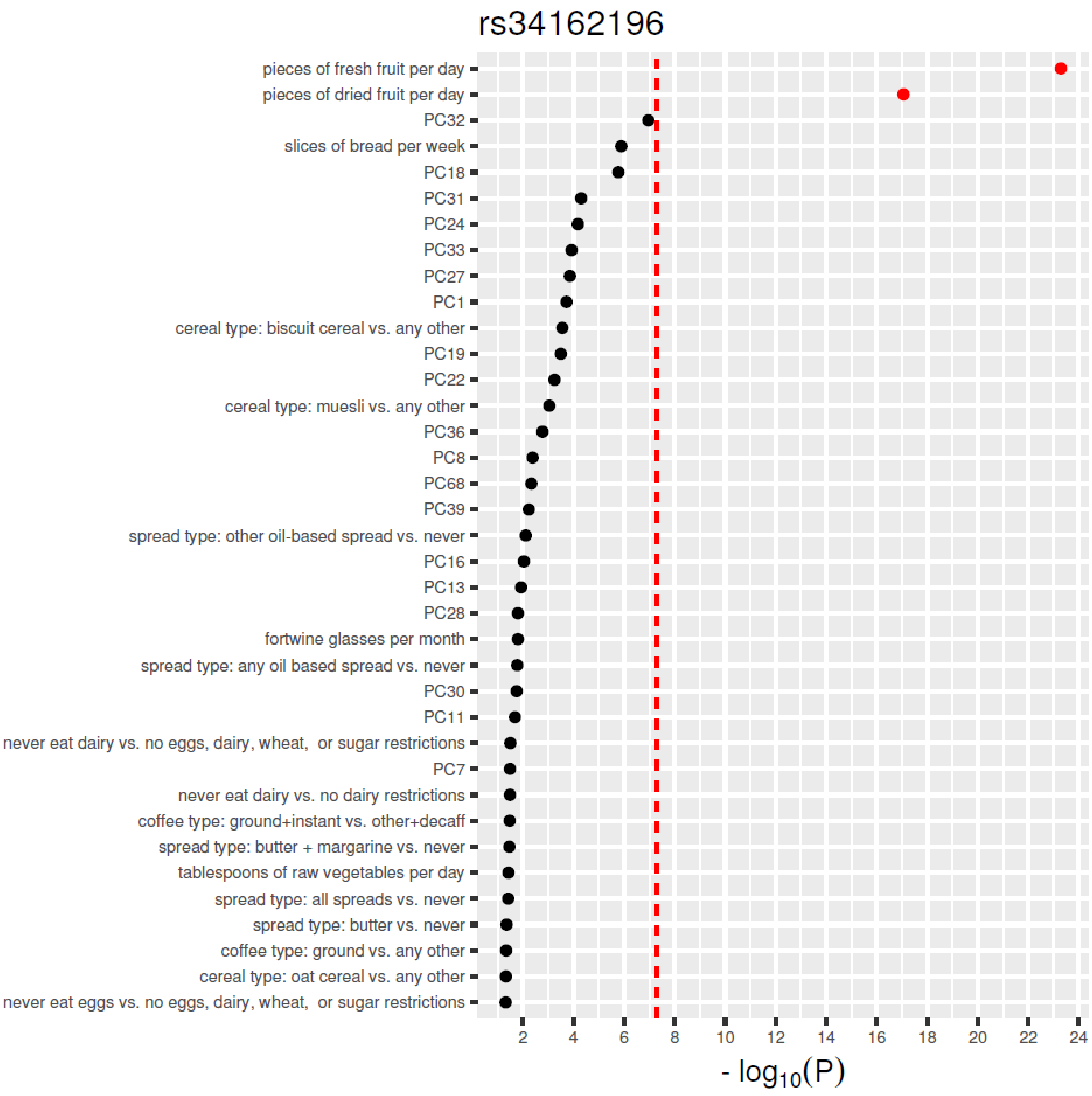

rs34162196
pieces of fresh fruit per day
pieces of dried fruit per day
PC32
slices of bread per week
PC18
PC31
PC24
PC33
PC27
PC1
cereal type: biscuit cereal vs. any other
PC19
PC22
cereal type: muesli vs. any other
PC36
PC8
PC68
PC39
spread type: other oil-based spread vs. never
PC16
PC13
PC28
fortwine glasses per month
spread type: any oil based spread vs. never
PC30
PC11
never eat dairy vs. no eggs, dairy, wheat, or sugar restrictions
PC7
never eat dairy vs. no dairy restrictions
coffee type: ground+instant vs. other+decaff
spread type: butter + margarine vs. never
tablespoons of raw vegetables per day
spread type: all spreads vs. never
spread type: butter vs. never
coffee type: ground vs. any other
cereal type: oat cereal vs. any other
never eat eggs vs. no eggs, dairy, wheat, or sugar restrictions
2 4 6 8 10 12 14 16 18 20 22 24
- $\log_{10}(P)$

c

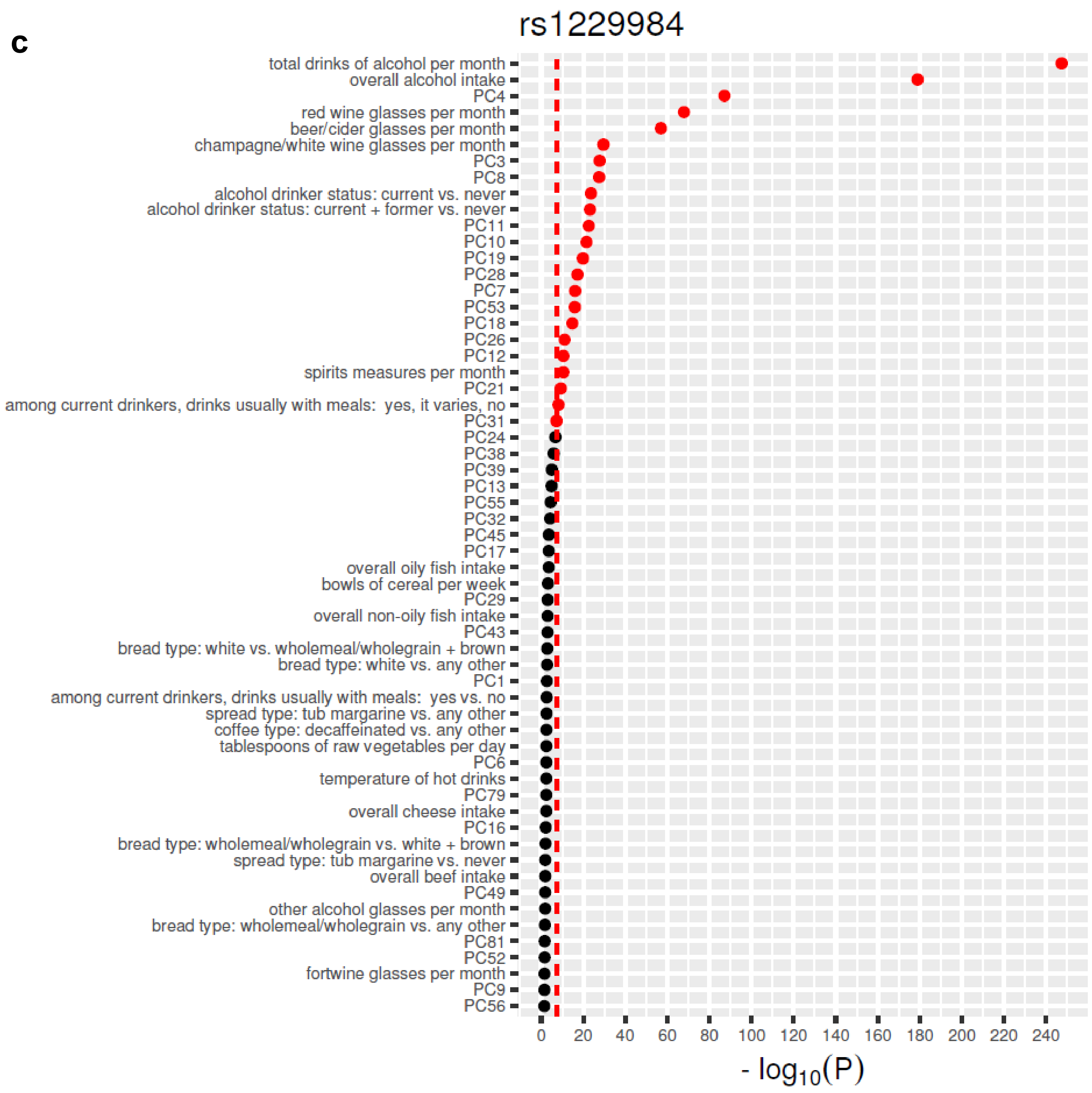
rs1229984
total drinks of alcohol per month
overall alcohol intake
PC4
red wine glasses per month
beer/cider glasses per month
champagne/white wine glasses per month
PC3
PC8
alcohol drinker status: current vs. never
alcohol drinker status: current + former vs. never
PC11
PC10
PC19
PC28
PC7
PC53
PC18
PC26
PC12
spirits measures per month
PC21
among current drinkers, drinks usually with meals: yes, it varies, no
PC31
PC24
PC38
PC39
PC13
PC55
PC32
PC45
PC17
overall oily fish intake
bowls of cereal per week
PC29
overall non-oily fish intake
PC43
bread type: white vs. wholemeal/wholegrain + brown
bread type: white vs. any other
PC1
among current drinkers, drinks usually with meals: yes vs. no
spread type: tub margarine vs. any other
coffee type: decaffeinated vs. any other
tablespoons of raw vegetables per day
PC6
temperature of hot drinks
PC79
overall cheese intake
PC16
bread type: wholemeal/wholegrain vs. white + brown
spread type: tub margarine vs. never
overall beef intake
PC49
other alcohol glasses per month
bread type: wholemeal/wholegrain vs. any other
PC81
PC52
fortwine glasses per month
PC9
PC56
0
20
40
60
80
100
120
140
160
180
200
220
240
- $\log_{10}(P)$

Supplementary Figure 7: LocusZoom plots. Close-up Manhattan (LocusZoom) plots displaying chromosomal position on the x-axis and $-\log_{10}$(P-value) on the y-axis. LD information is estimated from UKB and denoted by color (gray SNPs have $r^2<0.10$). SNPs within the 95% credible set are plotted as triangles. A) Chromosome 7q35 associated with "pieces of fresh fruit per day". B) Chromosome 14q11 associated with "pieces of fresh fruit per day" and "pieces of dried fruit per day". C) Chromosome 11p15 associated with butter consumption. D) Chromosome 14q11 associated with "tablespoons of raw vegetables per day". E) Chromosome 11q12 associated with "cups of tea per day". F) Chromosome 11q12 associated with "cups of coffee per day". G) Chromosome 11p15 associated with "overall cheese intake". H) Chromosome associated with PC1.

a

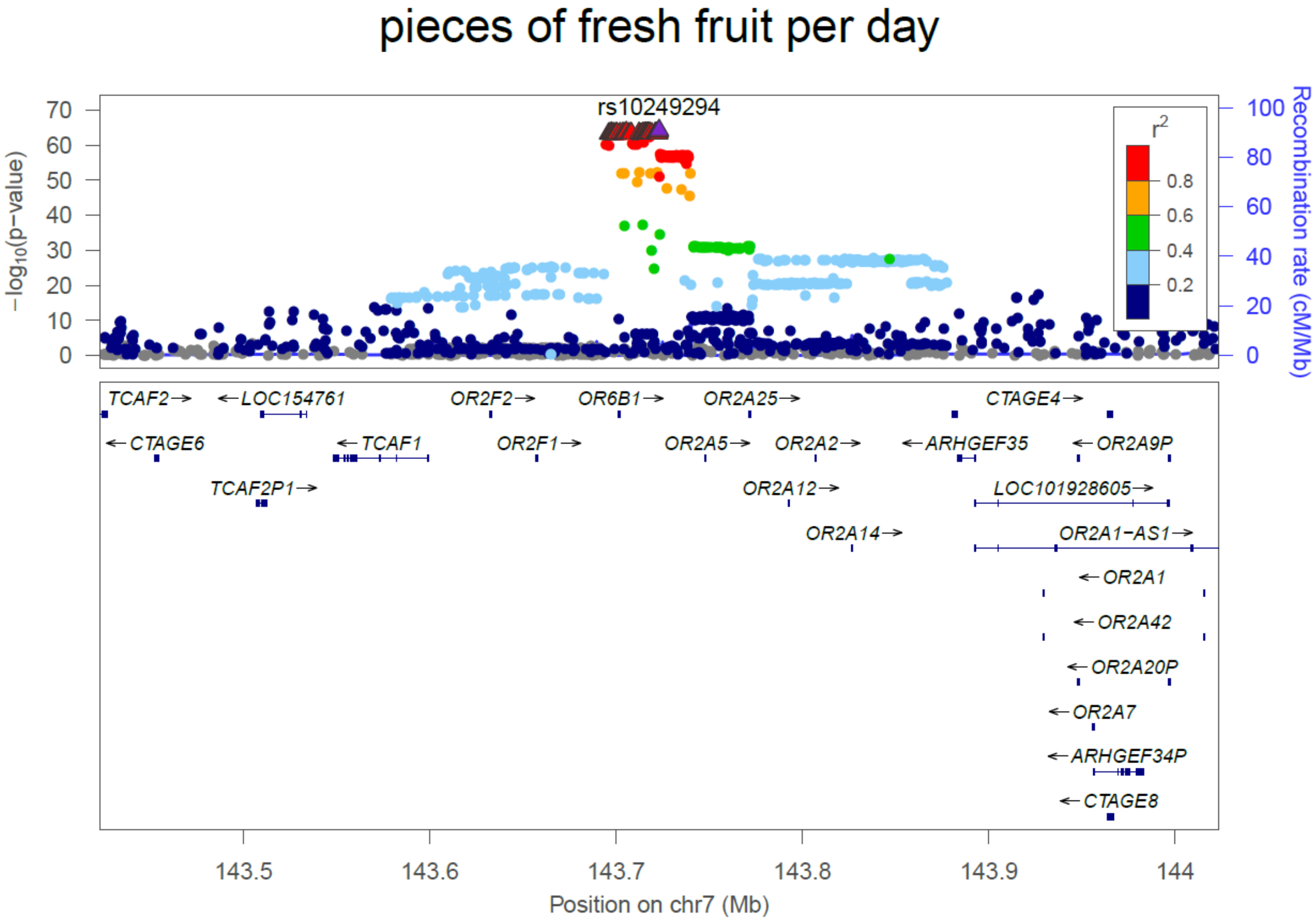

b

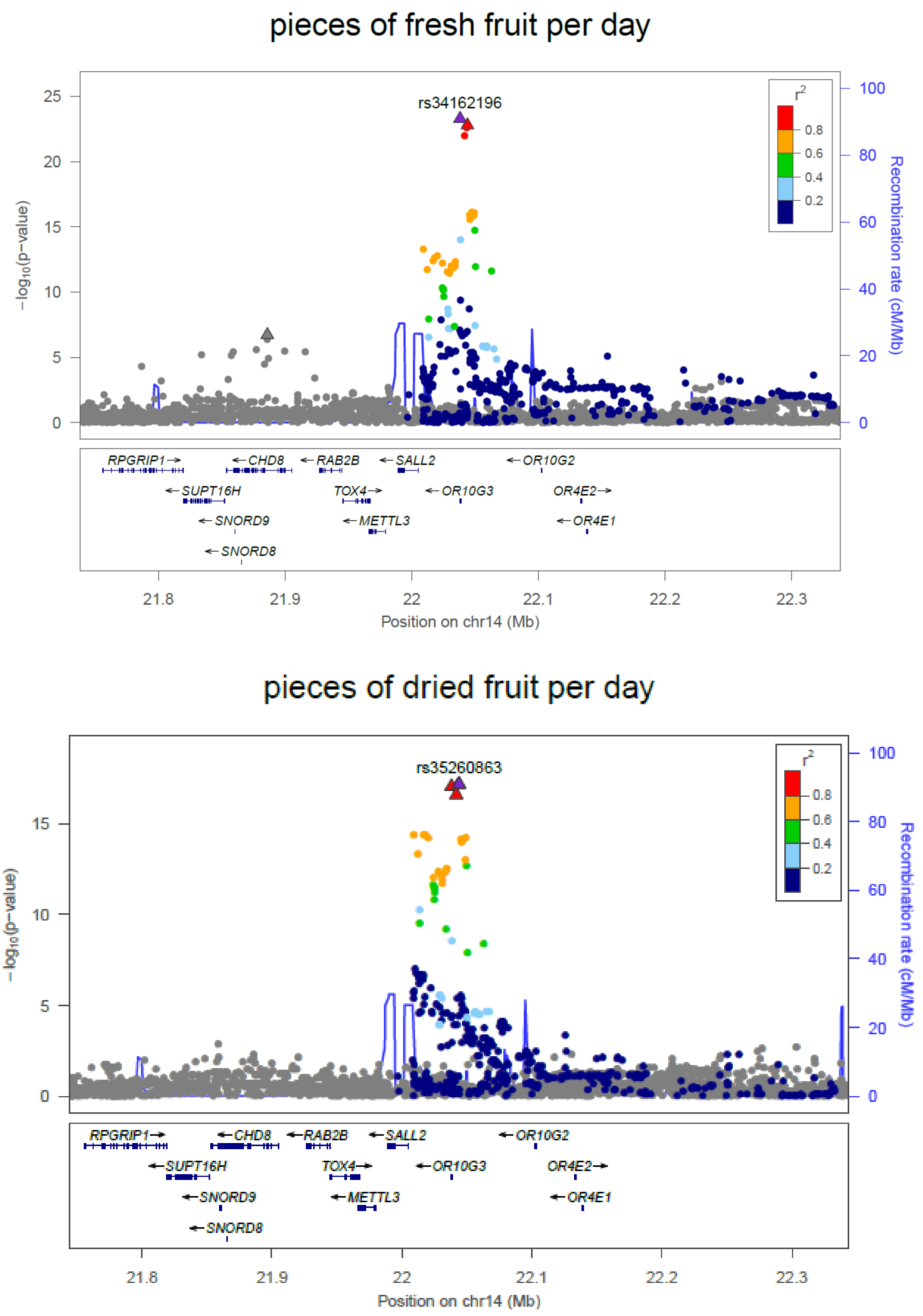
pieces of fresh fruit per day
rs34162196
r2
0.8
0.6
0.4
0.2
−log10(p−value)
Recombination rate (cM/Mb)
RPGRIP1
CHD8
RAB2B
SALL2
OR10G2
SUPT16H
TOX4
OR10G3
OR4E2
SNORD9
METTL3
OR4E1
SNORD8
21.8
21.9
22
22.1
22.2
22.3
Position on chr14 (Mb)
pieces of dried fruit per day
rs35260863
Position on chr14 (Mb)

c

spread type: butter vs. any other

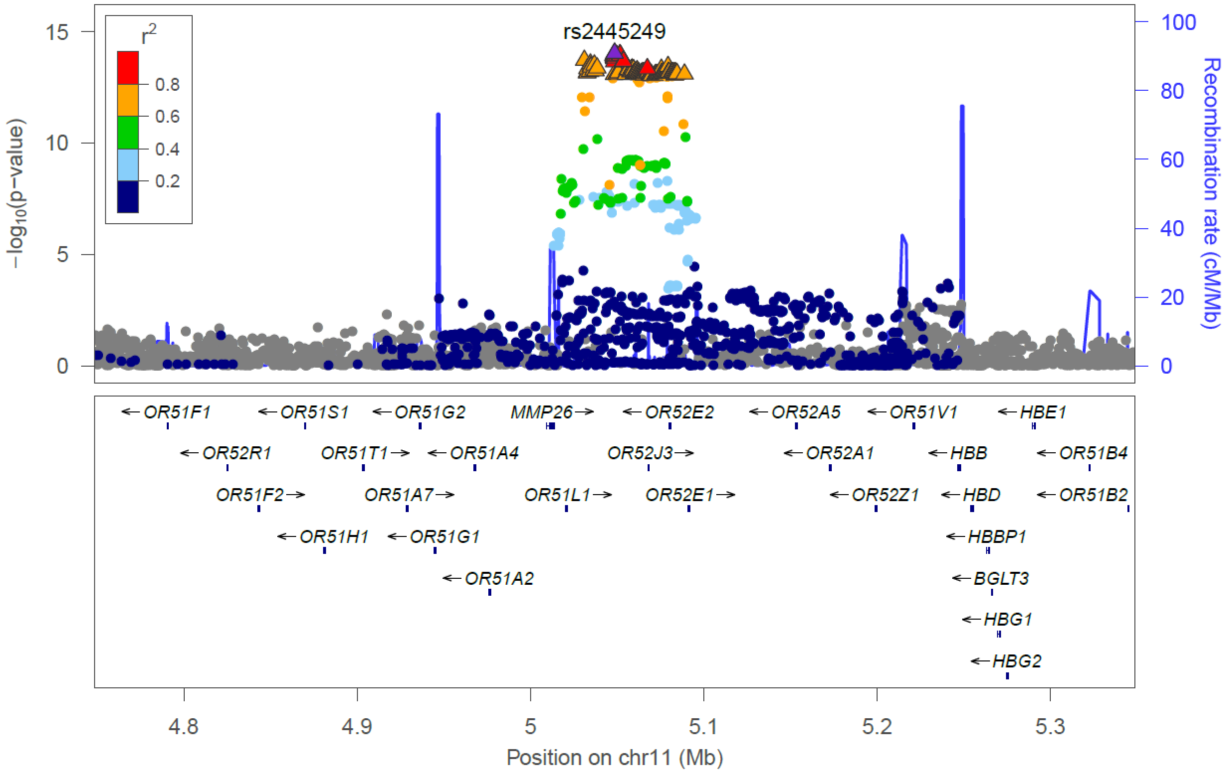

spread type: butter and margarine spreads vs. oil−based spreads

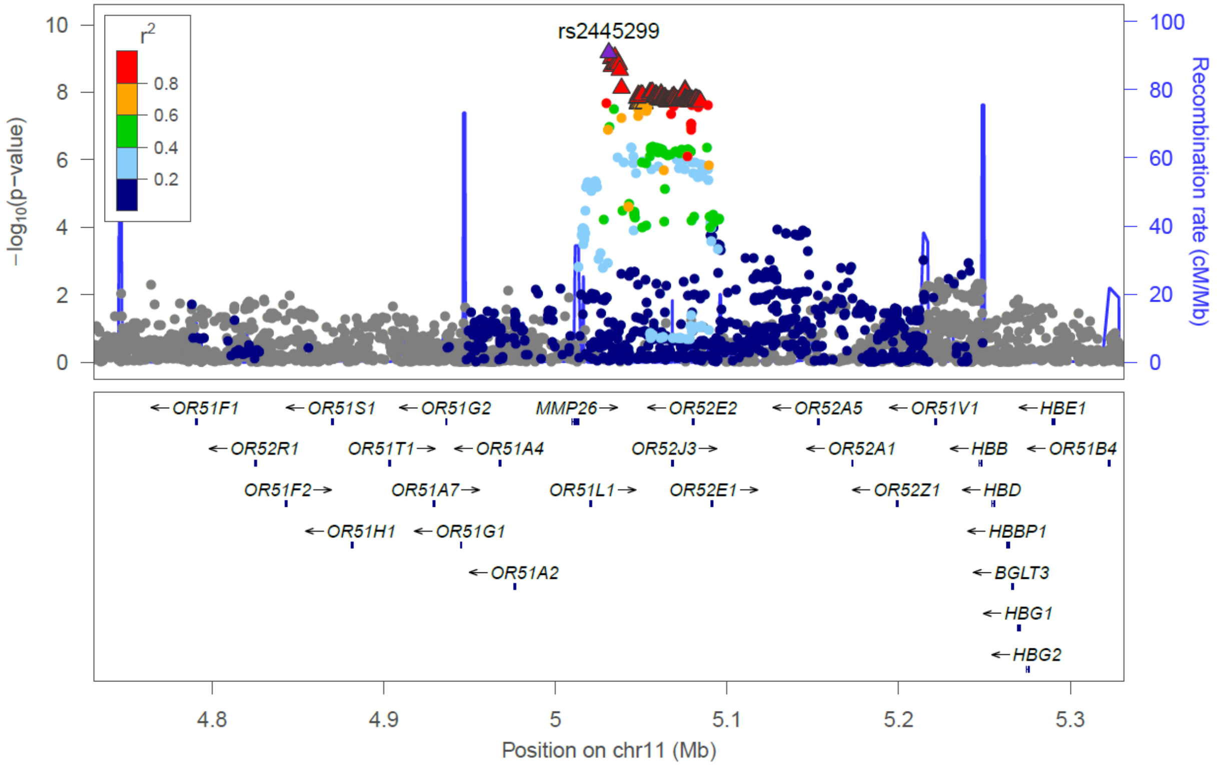

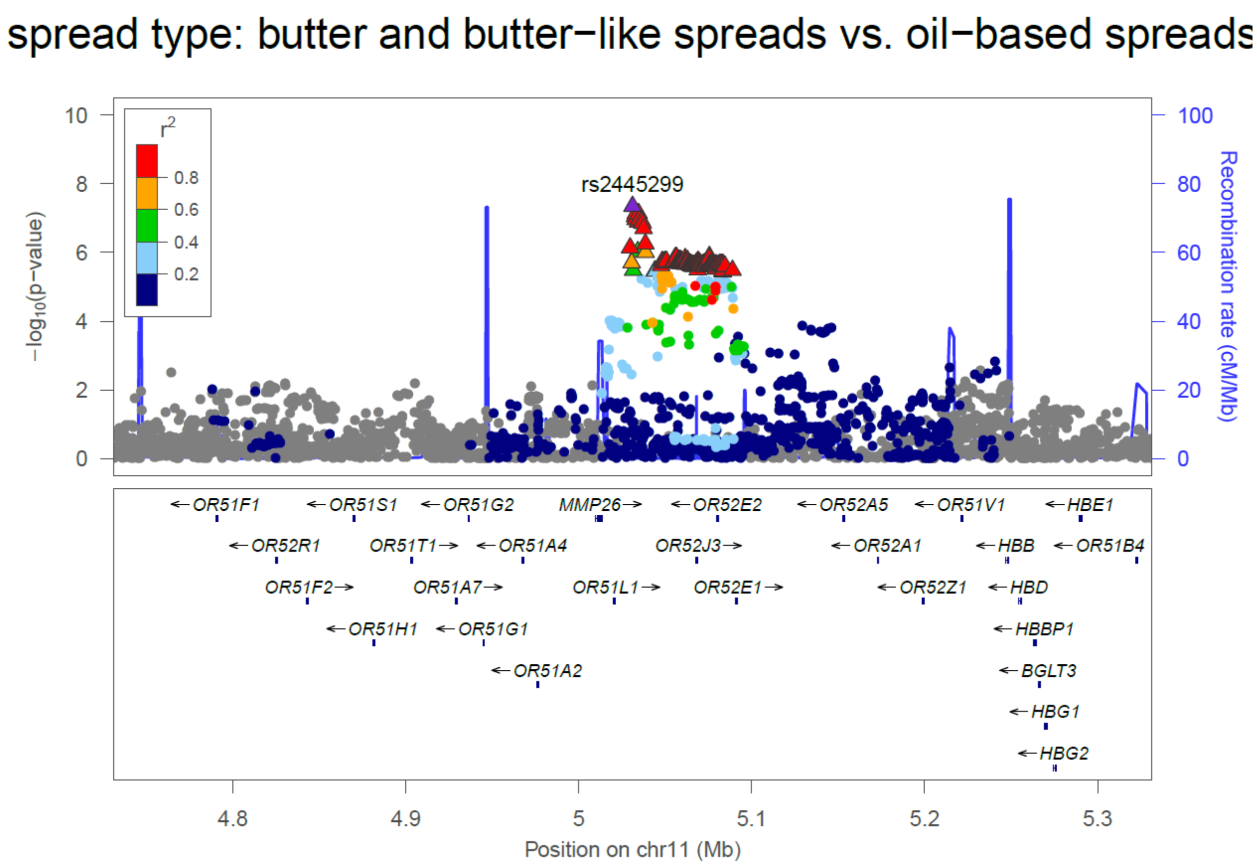
spread type: butter and butter−like spreads vs. oil−based spreads
rs2445299
r²
0.8
0.6
0.4
0.2
−log10(p−value)
Recombination rate (cM/Mb)
OR51F1
OR51S1
OR51G2
MMP26
OR52E2
OR52A5
OR51V1
HBE1
OR52R1
OR51T1
OR51A4
OR52J3
OR52A1
HBB
OR51B4
OR51F2
OR51A7
OR51L1
OR52E1
OR52Z1
HBD
OR51H1
OR51G1
HBBP1
OR51A2
BGLT3
HBG1
HBG2
4.8
4.9
5
5.1
5.2
5.3
Position on chr11 (Mb)

d

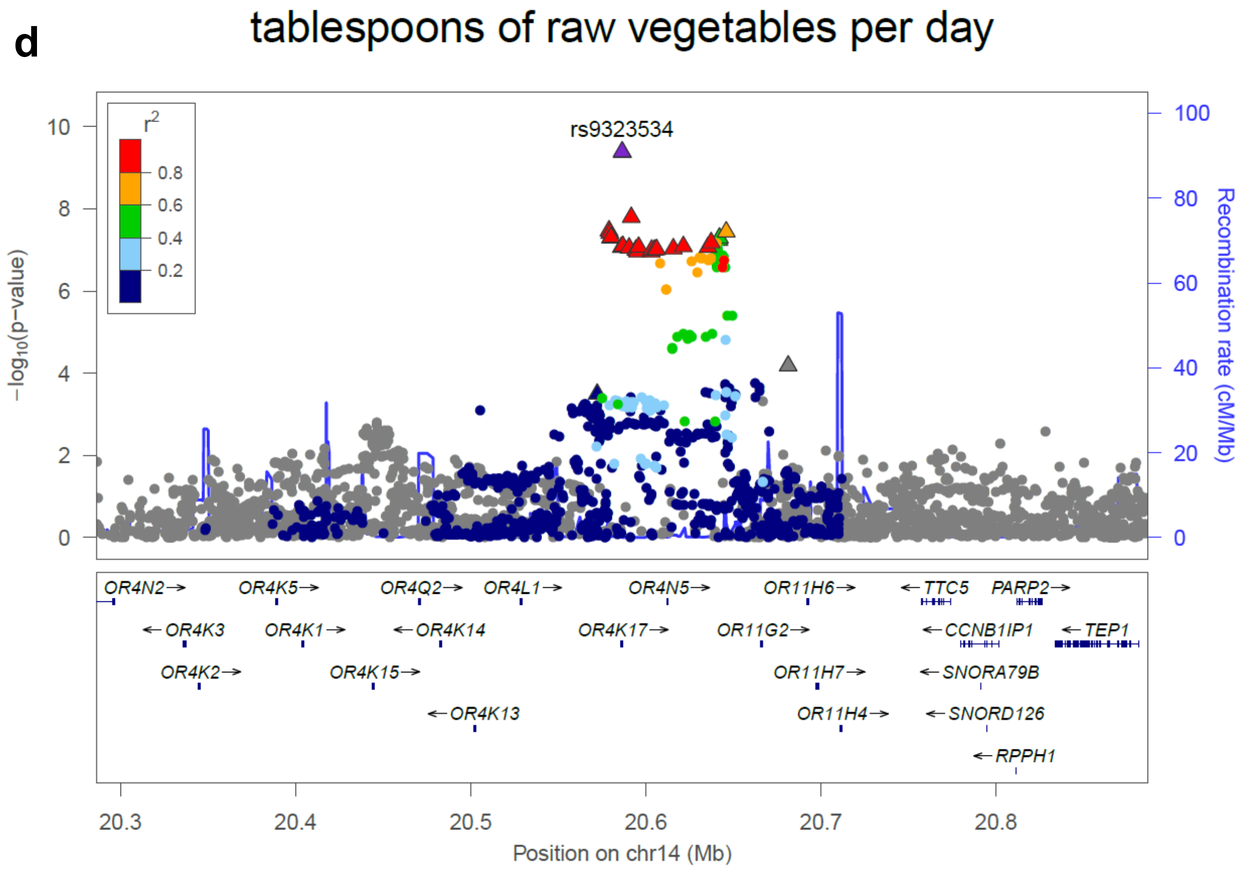
tablespoons of raw vegetables per day
rs9323534
r²
0.8
0.6
0.4
0.2
−log10(p−value)
Recombination rate (cM/Mb)
OR4N2
OR4K5
OR4Q2
OR4L1
OR4N5
OR11H6
TTC5
PARP2
OR4K3
OR4K1
OR4K14
OR4K17
OR11G2
CCNB1IP1
TEP1
OR4K2
OR4K15
OR11H7
SNORA79B
OR4K13
OR11H4
SNORD126
RPPH1
20.3
20.4
20.5
20.6
20.7
20.8
Position on chr14 (Mb)

e

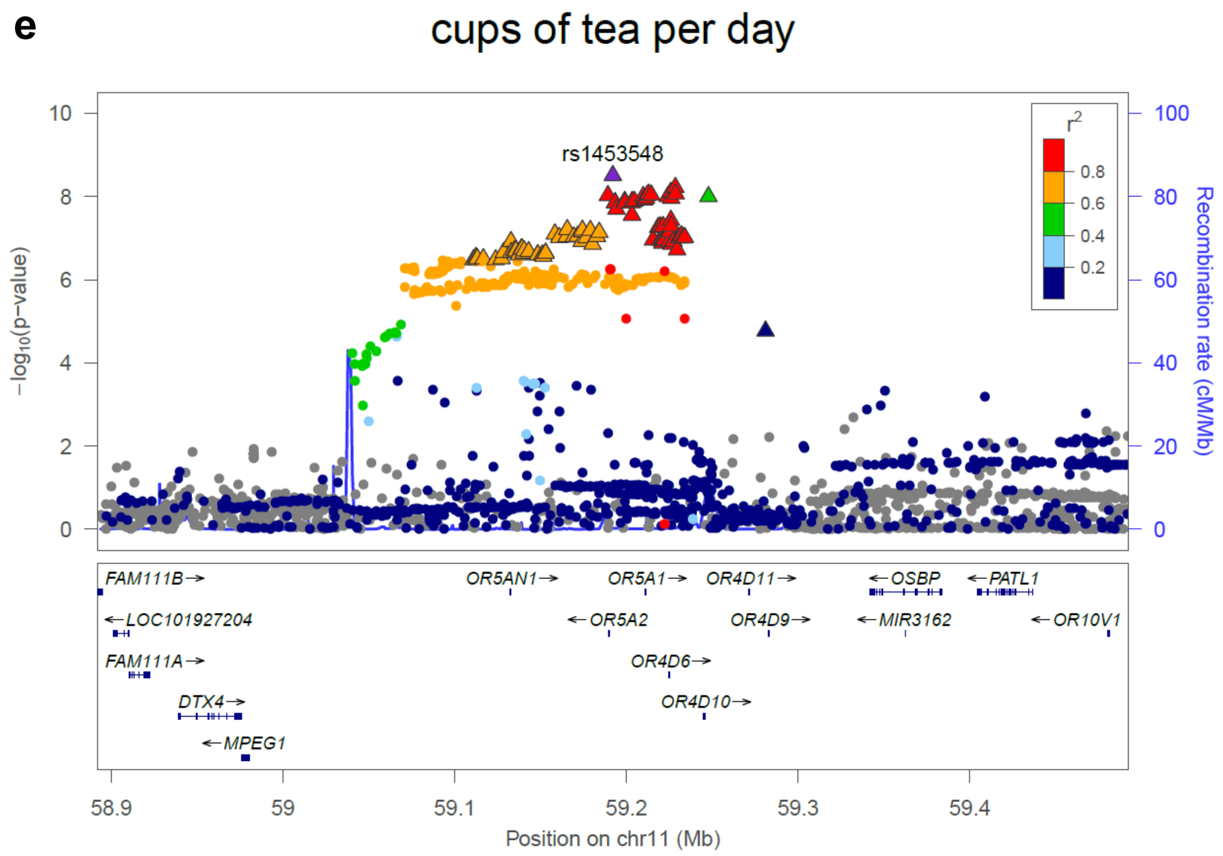

cups of tea per day
rs1453548
r2
0.8
0.6
0.4
0.2
−log10(p−value)
Recombination rate (cM/Mb)
FAM111B
LOC101927204
FAM111A
DTX4
MPEG1
OR5AN1
OR5A2
OR5A1
OR4D6
OR4D10
OR4D11
OR4D9
OSBP
MIR3162
PATL1
OR10V1
58.9
59
59.1
59.2
59.3
59.4
Position on chr11 (Mb)

f

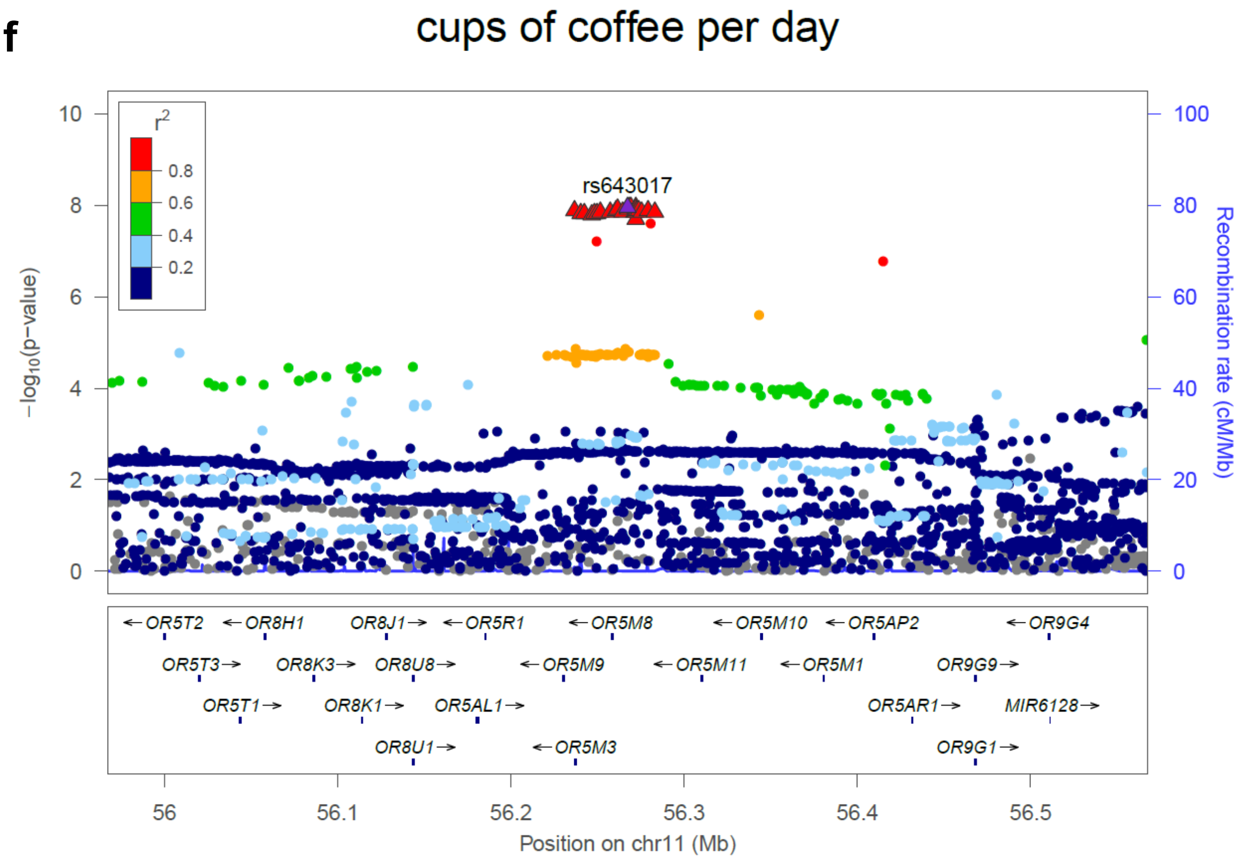

cups of coffee per day
rs643017
r2
0.8
0.6
0.4
0.2
−log10(p−value)
Recombination rate (cM/Mb)
OR5T2
OR8H1
OR8J1
OR5R1
OR5M8
OR5M10
OR5AP2
OR9G4
OR5T3
OR8K3
OR8U8
OR5M9
OR5M11
OR5M1
OR9G9
OR5T1
OR8K1
OR5AL1
OR5AR1
MIR6128
OR8U1
OR5M3
OR9G1
56
56.1
56.2
56.3
56.4
56.5
Position on chr11 (Mb)

g

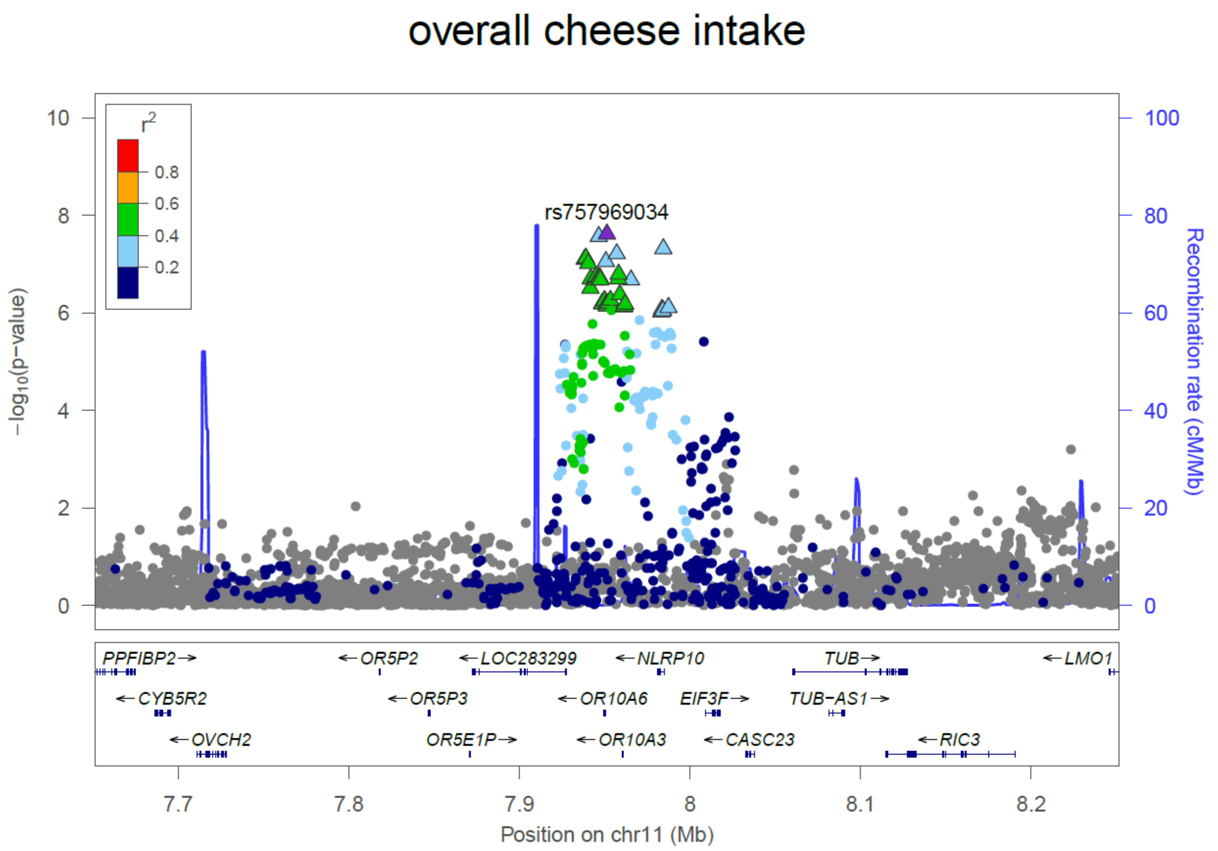
overall cheese intake
rs757969034
r2
0.8
0.6
0.4
0.2
−log10(p−value)
Recombination rate (cM/Mb)
PPFIBP2→
←OR5P2
←LOC283299
←NLRP10
TUB→
←LMO1
←CYB5R2
←OR5P3
←OR10A6
EIF3F→
TUB−AS1→
←OVCH2
OR5E1P→
←OR10A3
←CASC23
←RIC3
7.7
7.8
7.9
8
8.1
8.2
Position on chr11 (Mb)

h

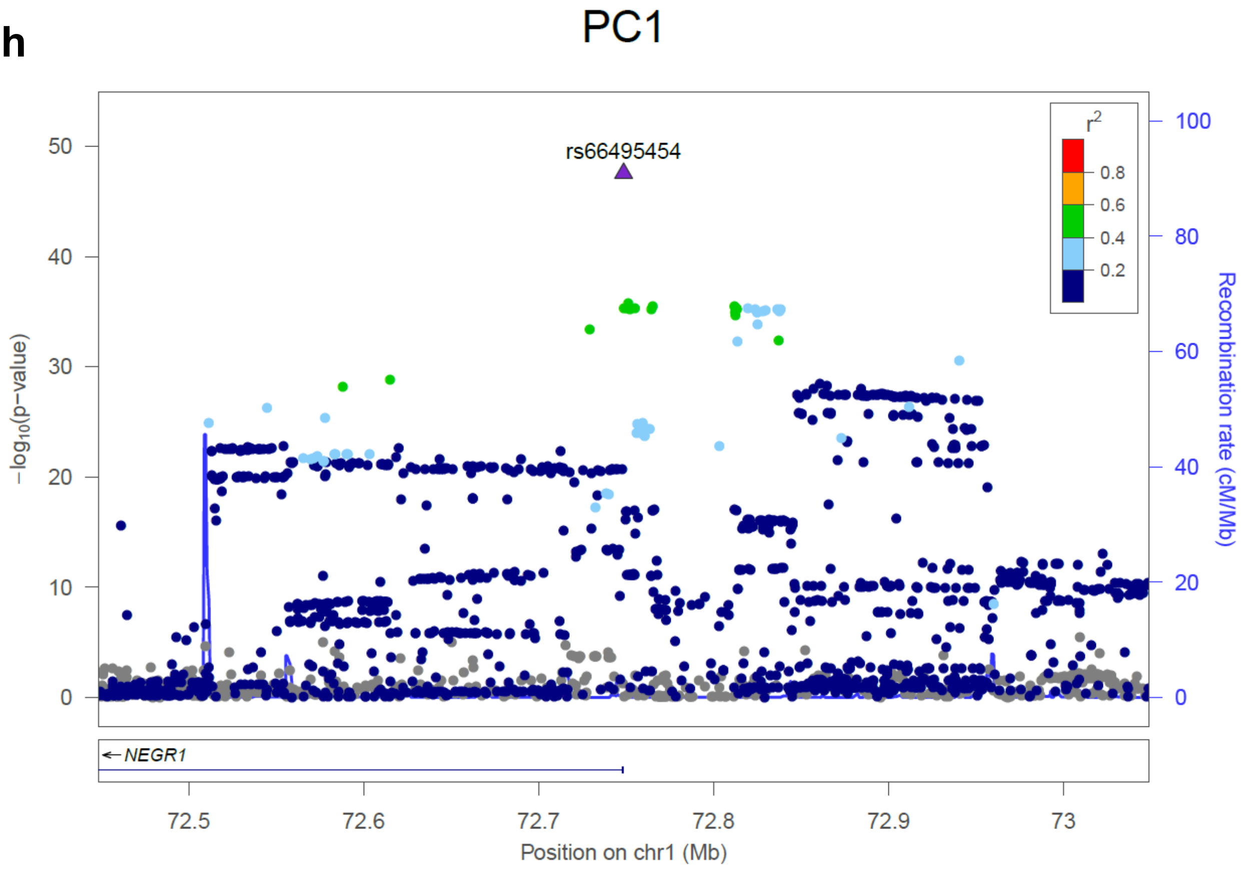
PC1
rs66495454
r2
0.8
0.6
0.4
0.2
−log10(p−value)
Recombination rate (cM/Mb)
←NEGR1
72.5
72.6
72.7
72.8
72.9
73
Position on chr1 (Mb)

Supplementary Figure 8: Bidirectional Mendelian Randomization with PC1 as the exposure and outcome with educational attainment, intelligence, and BMI. Scatter plots of beta coefficients with regression lines between exposure and outcome for the three MR methods (IVW-solid line, Egger-dashed, Weighted Median-dotted) before (left) and after (right) genetic instrument SNP filtering.

Exposure: PC1

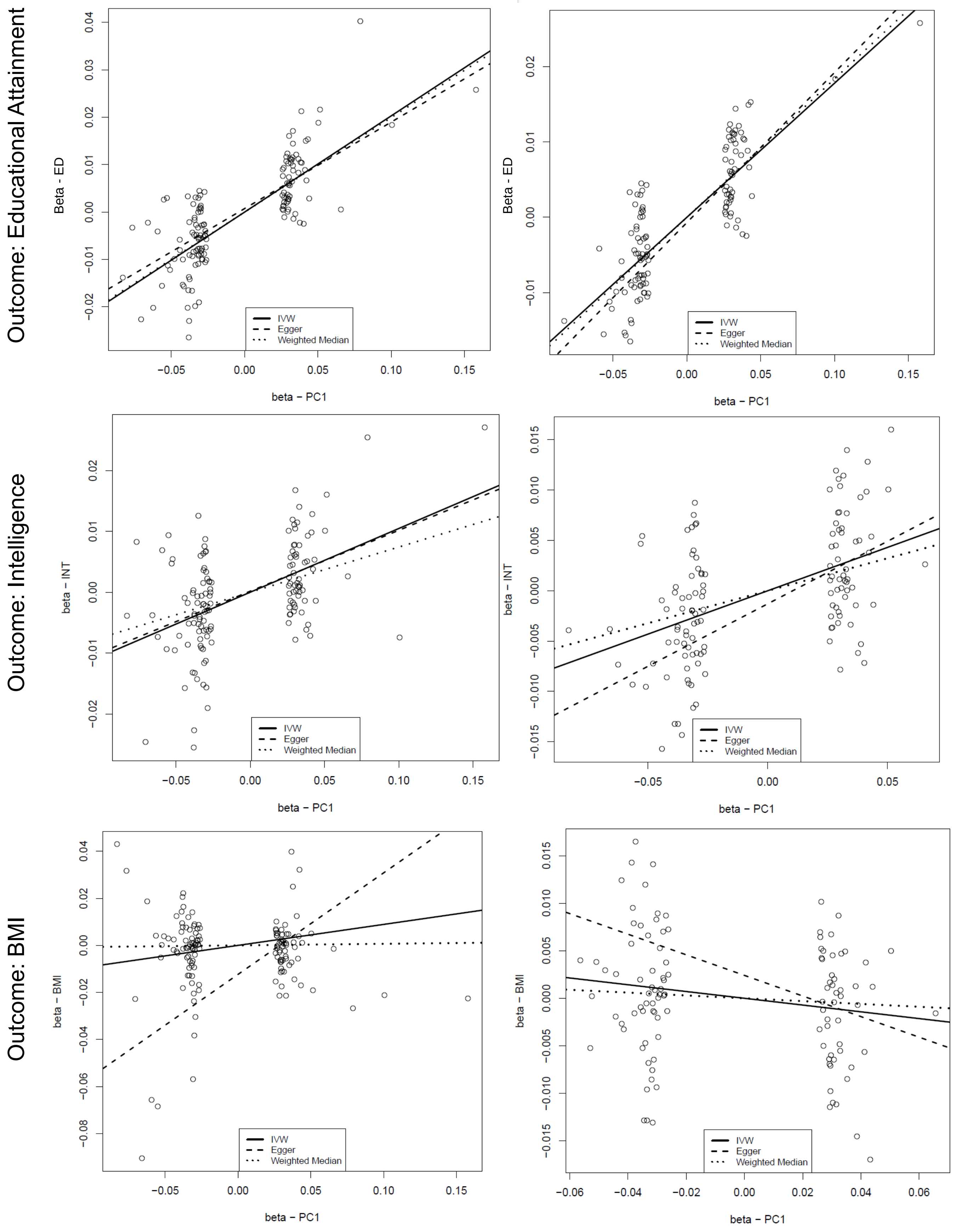

Exposure: Educational Attainment

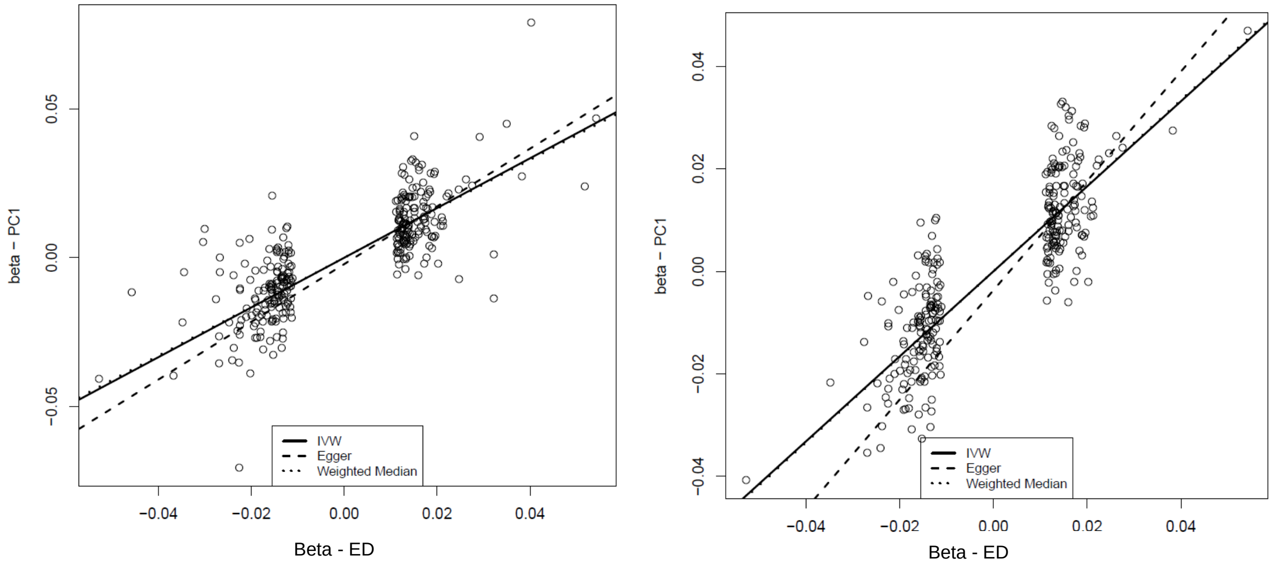

Exposure: Intelligence

Outcome: PC1

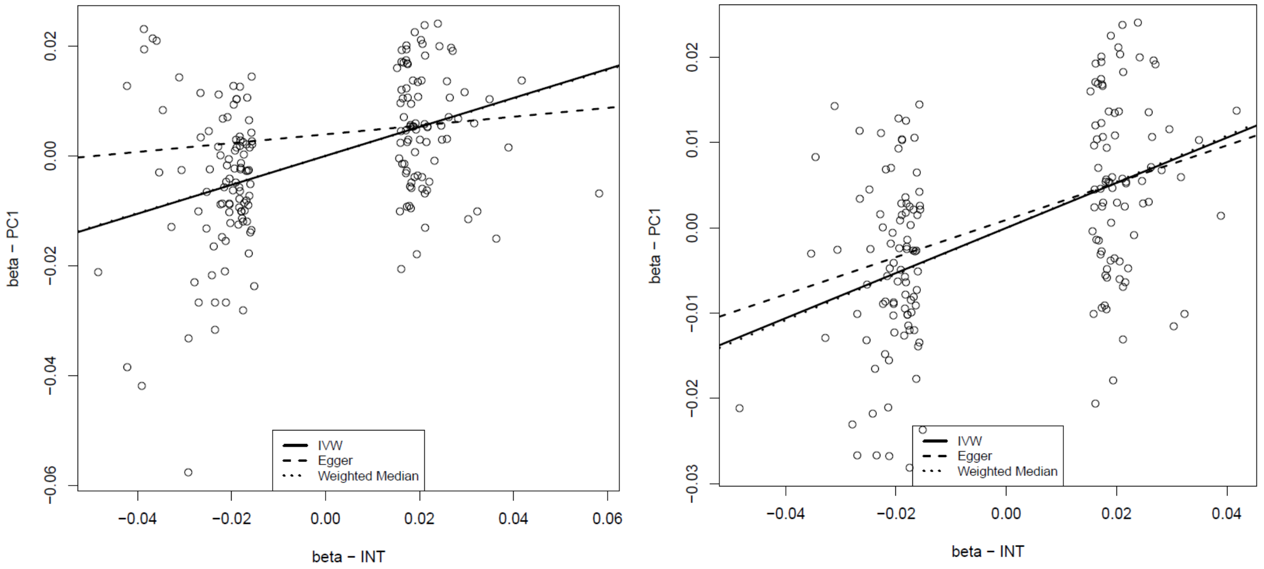

Exposure: BMI

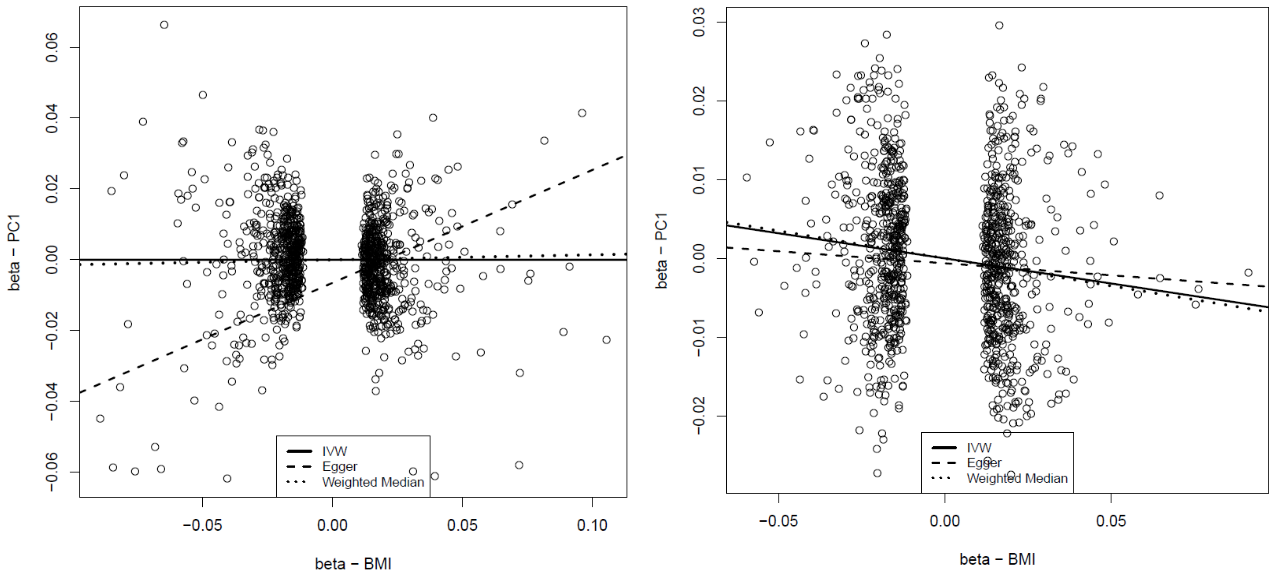

Supplementary Figure 9: P-value comparison of significant SNPs between two alcohol related phenotypes. Scatter plots of –$\log_{10}$(P-value) between "total drinks per month" and "overall alcohol intake" with the line of identity (solid black) and the regression line (dotted red line) A) with all SNPs B) excluding lead SNP rs1229984.

a

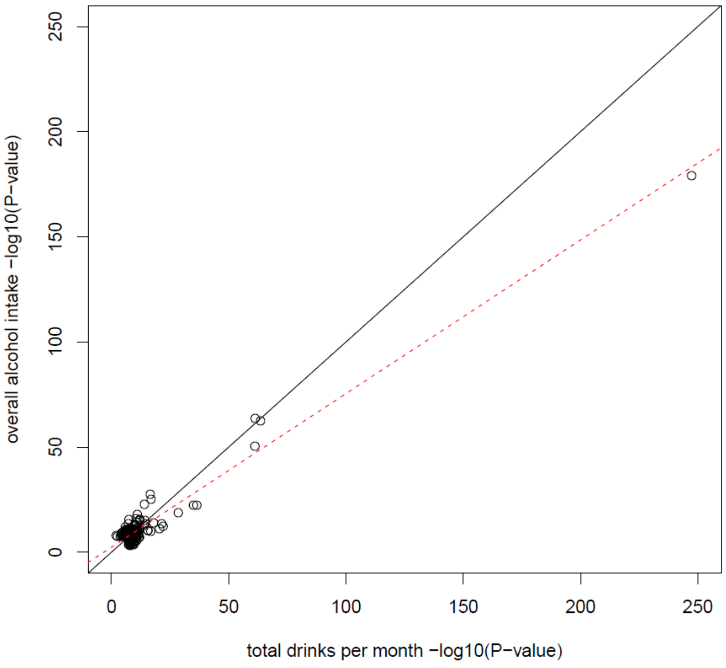

b

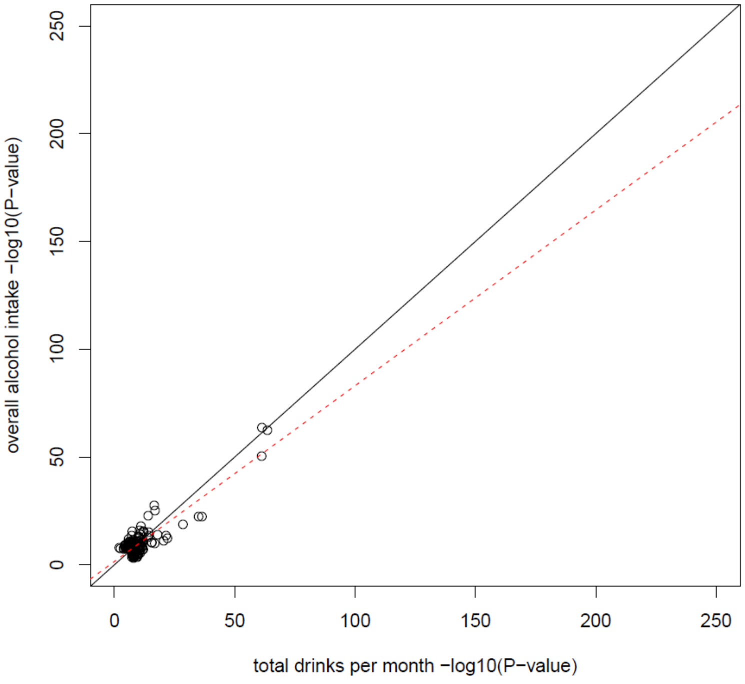

Supplementary Figure 10: Correlation matrix of alcohol-related traits. Phenotypic correlation (upper triangle) and genetic correlation (lower triangle) between correlated alcohol-related traits. All correlations with nonsignificant p-values ($P > 0.05/85 = 5.88 \times 10^{-4}$) were set to 0.

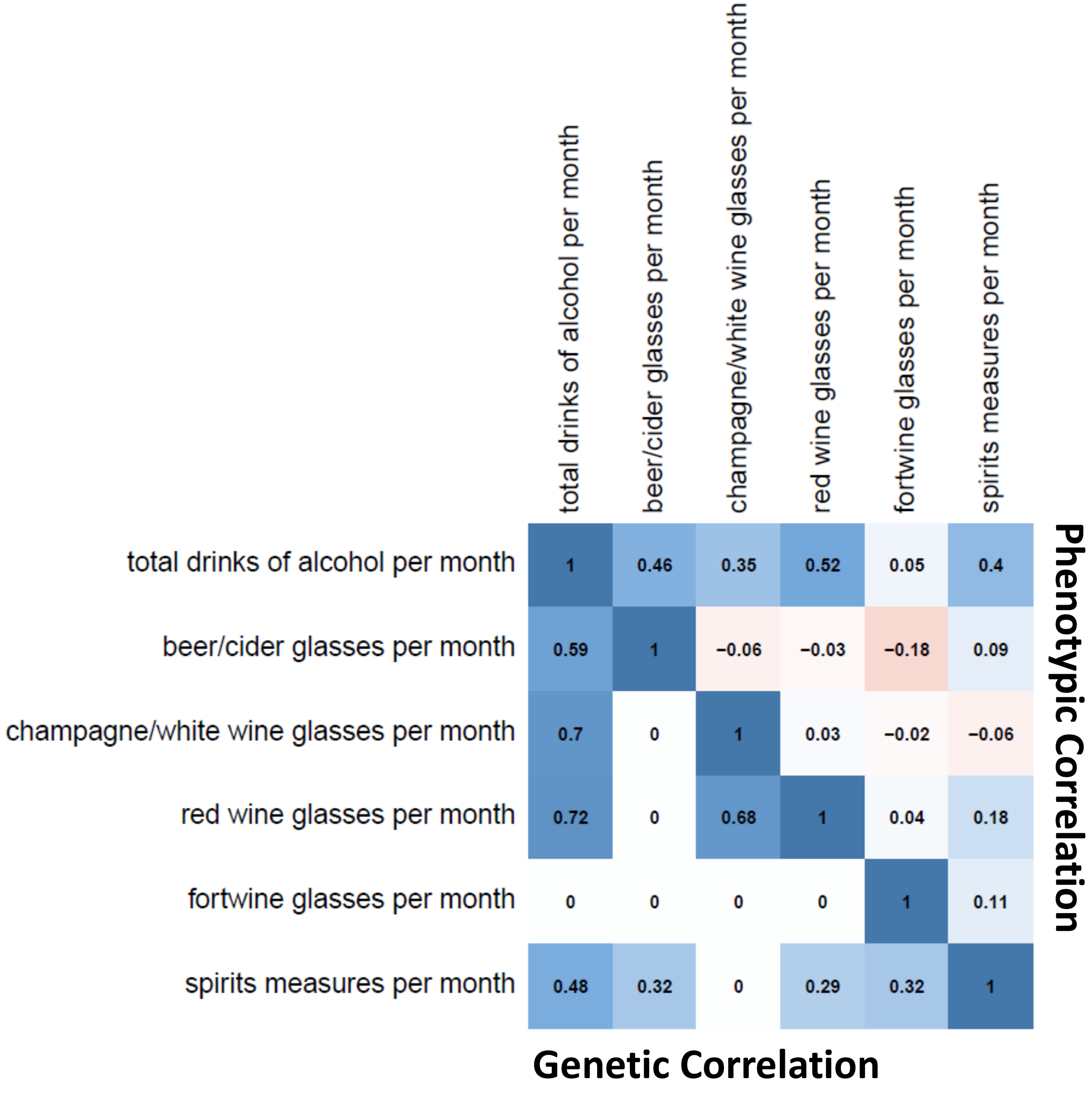

Supplementary Figure 11: Heritability estimates between BOLT-lmm pseudo-h2g and LD Score regression heritability estimates.

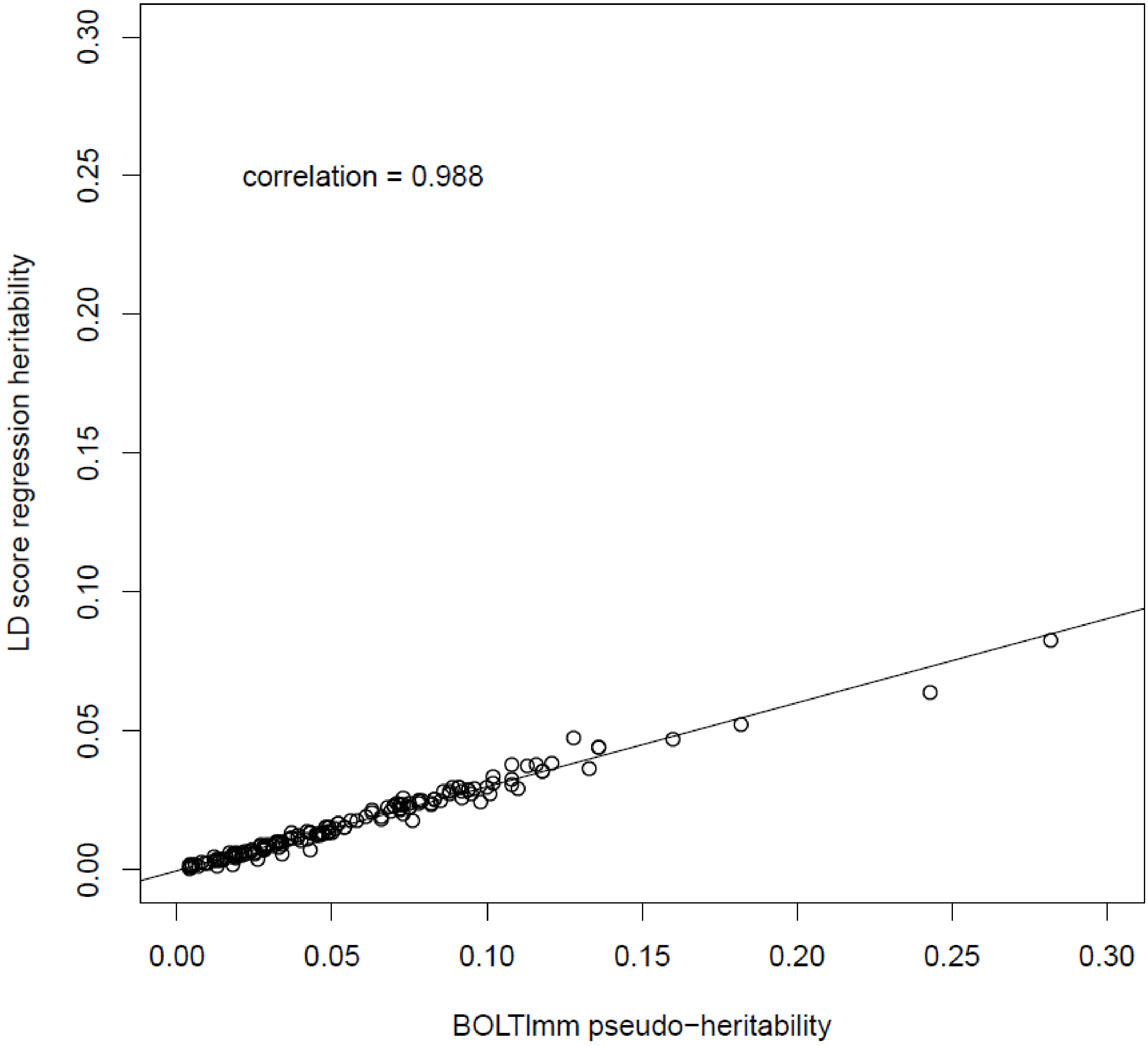

Supplementary Table 1. Bidirectional Mendelian randomization results for PC1 after SNP filtering. SNP filtering includes Steiger filtering to remove variants likely influenced by reverse causation and Cook's distance filtering to remove outlying heterogeneous variants

			Inverse Variance				MR Egger						Weighted Median			
**Expo-sure**	**Out-come**	**SNPs**	**Est.**	**CI Lower**	**CI Upper**	**P-value**	**Est.**	**CI Lower**	**CI Upper**	**P-value**	**Intercept**	**Int. P-value**	**Est.**	**CI Lower**	**CI Upper**	**P-value**
ED	PC1	288	0.829	0.765	0.893	7.80×10^{-142}	1.066	0.776	1.356	5.46×10^{-13}	-0.004	0.100	0.835	0.763	0.907	2.79×10^{-114}
PC1	ED	121	0.178	0.152	0.204	7.07×10^{-42}	0.199	0.067	0.332	3.14×10^{-3}	-0.001	0.749	0.184	0.159	0.209	1.87×10^{-47}
INT	PC1	172	0.265	0.189	0.340	5.41×10^{-12}	0.218	-0.170	0.607	0.271	0.001	0.812	0.270	0.200	0.341	6.01×10^{-14}
PC1	INT	126	0.086	0.058	0.115	3.72×10^{-9}	0.124	-0.014	0.263	0.079	-0.001	0.582	0.065	0.034	0.095	2.78×10^{-5}
BMI	PC1	1081	-0.064	-0.096	-0.032	8.76×10^{-5}	-0.031	-0.145	0.084	0.601	-0.001	0.552	-0.070	-0.102	-0.037	2.40×10^{-5}
PC1	BMI	110	-0.036	-0.072	0.001	0.055	-0.108	-0.319	0.102	0.314	0.002	0.492	-0.015	-0.041	0.012	0.272

Supplementary Table 2. Mendelian Randomization validation and sensitivity results for educational attainment and PC1. A) Validation of educational attainment (ED) causal effect on PC1 using an external dataset not including UKB. B) Bidirectional MR validation of educational attainment and PC1 using 2 subsets of UKB: a training dataset to derive the genetic instrumental variable and a testing subset to assess causality. C) Bidirectional sensitivity analysis with PC1 GWAS additionally adjusted for assessment center in the linear mixed model.

A) Validation of educational attainment (ED) causal effect on PC1 using an external dataset not including UKB (Okbay et al, Nature 2016).

			Inverse Variance				MR Egger						Weighted Median			
Expo-sure	Out-come	SNPs	Est.	CI Lower	CI Upper	P-value	Est.	CI Lower	CI Upper	P-value	Intercept	Int. P-value	Est.	CI Lower	CI Upper	P-value
ED	PC1	66	0.733	0.566	0.9	7.39 x 10^{-18}	1.201	0.432	1.97	2.19 x 10^{-3}	-0.009	0.221	0.708	0.576	0.84	6.23 x 10^{-26}

B) Bidirectional MR validation of educational attainment and PC1 using 2 subsets of UKB: a training dataset to derive the genetic instrumental variable (1/3 EUR N~150K) and a testing subset to assess causality (2/3 EUR N~300K)

			Inverse Variance				MR Egger						Weighted Median			
Expo-sure	Out-come	SNPs	Est.	CI Lower	CI Upper	P-value	Est.	CI Lower	CI Upper	P-value	Intercept	Int. P-value	Est.	CI Lower	CI Upper	P-value
PC1	ED	13	0.137	0.076	0.199	1.02 x 10^{-13}	0.075	-0.247	0.396	0.0103	0.004	0.696	0.11	0.062	0.158	4.39 x 10-$^{-22}$
ED	PC1	32	0.809	0.596	1.022	1.11 x 10^{-5}	2.116	0.5	3.732	0.65	-0.032	0.11	0.826	0.658	0.993	6.91 x 10^{-6}

C) Bidirectional sensitivity analysis with PC1 GWAS additionally adjusted for assessment center in the linear mixed model.

			Inverse Variance				MR Egger						Weighted Median			
Expo-sure	Out-come	SNPs	Est.	CI Lower	CI Upper	P-value	Est.	CI Lower	CI Upper	P-value	Intercept	Int. P-value	Est.	CI Lower	CI Upper	P-value
PC1	ED	126	0.203	0.172	0.235	1.02 x 10^{-37}	0.143	0.018	0.269	0.025	0.0021	0.331	0.194	0.168	0.22	4.39 x 10^{-49}
ED	PC1	309	0.79	0.72	0.86	6.31 x 10^{-108}	0.916	0.629	1.203	3.94 x 10^{-10}	-0.002	0.376	0.807	0.736	0.878	2.30 x 10^{-110}

Supplementary Table 3. Mendelian randomization results between educational attainment and PC1's contributing FI-QTs. Weighed Median MR with educational attainment from UKB as the exposure and PC1 and its's 19 FI-QTs as the outcome.

Outcome	Estimate	CI lower	CI upper	P-value
**PC1**	0.823	0.751	0.894	6.59E-113
**bread type: wholemeal/wholegrain vs. white + brown**	0.397	0.362	0.433	1.97E-106
**bread type: wholemeal/wholegrain vs. any other**	0.366	0.332	0.401	2.59E-95
**bread type: white vs. any other**	-0.357	-0.389	-0.325	6.18E-107
**bread type: white vs. wholemeal/wholegrain + brown**	-0.363	-0.396	-0.330	1.10E-101
**pieces of fresh fruit per day**	0.081	0.050	0.113	4.18E-07
**spread type: all spreads vs. never**	-0.050	-0.071	-0.028	7.65E-06
**spread type: butter + margarine vs. never**	-0.090	-0.122	-0.057	4.91E-08
**spread type: butter vs. never**	-0.077	-0.118	-0.036	0.000232102
**overall processed meat intake**	-0.017	-0.047	0.012	0.252382614
**milk type: skimmed, semi-skimmed, full cream (QT)**	0.049	0.021	0.078	0.000685274
**overall oily fish intake**	0.141	0.111	0.172	9.30E-20
**tablespoons of raw vegetables per day**	0.015	-0.015	0.045	0.342731568
**pieces of dried fruit per day**	0.253	0.223	0.283	4.61E-62
**spread type: any oil based spread vs. never**	-0.066	-0.111	-0.021	0.004317514
**spread type: butter vs. any other**	0.032	-0.001	0.066	0.061011324
**spread type: other oil-based spread vs. never**	-0.167	-0.225	-0.109	1.86E-08
**spread type: olive oil spread vs. never**	0.015	-0.051	0.081	0.655263859
**glasses of water per day**	0.072	0.041	0.102	5.18E-06
**tablespoons of cooked vegetables per day**	0.047	0.017	0.076	0.002009479

Supplementary Table 4. Bidirectional Mendelian randomization results for diet and coronary artery disease (CAD) and type 2 diabetes (T2D). Results are shown both for the original GWAS results for PC1 (top table) and a sensitivity analysis adjusting PC1 additionally for assessment center in the linear mixed model (bottom table).

			**Inverse Variance**				**MR Egger**						**Weighted Median**			
**Expo-sure**	**Out-come**	**SNPs**	**Est.**	**CI Lower**	**CI Upper**	**P-value**	**Est.**	**CI Lower**	**CI Upper**	**P-value**	**Intercept**	**Int. P-value**	**Est.**	**CI Lower**	**CI Upper**	**P-value**
**CAD**	**PC1**	**38**	**0.0711**	**0.0199**	**0.1223**	**0.0065**	**0.0271**	**-0.0823**	**0.1364**	**0.6275**	**0.005**	**0.3711**	**0.0458**	**0.0159**	**0.0757**	**0.0027**
**PC1**	**CAD**	**105**	**0.0713**	**-0.0135**	**0.156**	**0.0992**	**0.7423**	**0.4487**	**1.0358**	**7.22×10^{-7}**	**-0.0241**	**3.42×10^{-6}**	**0.0722**	**-0.0261**	**0.1706**	**0.15**
**T2D**	**PC1**	**37**	**-0.0028**	**-0.0389**	**0.0333**	**0.88**	**0.0358**	**-0.0579**	**0.1296**	**0.4537**	**-0.0045**	**0.3815**	**0.0191**	**-0.0069**	**0.0451**	**0.1496**
**PC1**	**T2D**	**94**	**0.1341**	**-0.0186**	**0.2867**	**0.0852**	**0.306**	**-0.3059**	**0.918**	**0.327**	**-0.0061**	**0.5694**	**0.0994**	**-0.0441**	**0.2428**	**0.1745**

			**Inverse Variance**				**MR Egger**						**Weighted Median**			
**Expo-sure**	**Out-come**	**SNPs**	**Est.**	**CI Lower**	**CI Upper**	**P-value**	**Est.**	**CI Lower**	**CI Upper**	**P-value**	**Intercept**	**Int. P-value**	**Est.**	**CI Lower**	**CI Upper**	**P-value**
**CAD**	**PC1**	**38**	**0.0720**	**0.0205**	**0.1235**	**0.0062**	**0.0268**	**-0.0832**	**0.1368**	**0.6328**	**0.0052**	**0.3619**	**0.0463**	**0.0164**	**0.0762**	**0.0024**
**PC1**	**CAD**	**93**	**0.0855**	**-0.0048**	**0.1759**	**0.0635**	**0.7948**	**0.4863**	**1.1034**	**4.43×10^{-7}**	**-0.0254**	**2.98×10^{-6}**	**0.0983**	**-0.0046**	**0.2013**	**0.0612**
**T2D**	**PC1**	**37**	**-0.0037**	**-0.0399**	**0.0326**	**0.8435**	**0.0323**	**-0.0620**	**0.1267**	**0.5019**	**-0.0042**	**0.4178**	**0.0195**	**-0.0062**	**0.0452**	**0.1364**
**PC1**	**T2D**	**81**	**0.1545**	**-0.0156**	**0.3246**	**0.0750**	**0.4269**	**-0.2562**	**1.1101**	**0.2206**	**-0.0096**	**0.4196**	**0.1181**	**-0.0374**	**0.2736**	**0.1365**
