# Supplementary material for: Comprehensive genomic analysis of dietary habits in UK Biobank identifies hundreds of genetic associations
Source: Nat Commun. 2020 Mar 19;11:1467. doi: 10.1038/s41467-020-15193-0 (PMC7081342; doi:10.1038/s41467-020-15193-0)
Supplement: Supplementary file 10 — Description of Additional Supplementary Files [file 41467_2020_15193_MOESM10_ESM.pdf]

Title: Supplementary Data 1. Heritability estimates for 170 dietary habit phenotypes.

Description: A complete list of all 170 derived dietary habits, UKB field corresponding to the FFQ question for which the dietary habit was derived, sample size (N), SNP heritability estimates on the observed scale (h2g) with standard error (SE) and P-value, SNP heritability on the liability scale if the trait was originally binary (though all traits after averaging, covariate adjustment, and transformation are continuous), LD score regression (LDSC) intercept, LDSC ratio, significant number of GWAS loci (GWAS loci count), and principal components analysis PC eigenvalues (EV) and proportion of variance explained (Prop VarEx). Significant observed h2g estimates are in bold.

Title: Supplementary Data 2. Dietary habit phenotype correlation matrix.

Description: Pair-wise phenotypic correlation matrix of 85 single food intake QT. All non-significant correlations were set to 0 (P > 0.05/85 = 5.88 x 10-4).

Title: Supplementary Data 3. Dietary habit genetic correlation matrix.

Description: Genetic correlation matrix of 143 significantly heritable QT and PC dietary habits, as estimated by LD score regression software on GWAS summary statistics. All non-significant correlations were set to 0 (P > 0.05/85 = 5.88 x 10-4).

Title: Supplementary Data 4. Eight-hundred fourteen lead index SNP GWAS results.

Description: GWAS results include the lead index SNP in each 500kb clumped locus with its most significantly associated dietary habit. The SNP is considered novel if the locus as defined by the 95% credible set SNPs and SNPs in high LD (r2>0.8) with any of the 95% credible set SNPs have not been previously reported in the GWAS catalog or Neale Lab UKB GWAS at genome-wide significance. We also include the number of SNPs in the 95% credible set, nearest gene, and distance to the nearest gene.

Title: Supplementary Data 5. Genetic correlation matrix between 143 significantly heritable dietary habits and 3,219 Neale Lab UKB GWAS traits.

Description: Estimates were obtained from LD Score Regression software, and all genetic correlation estimates with P-values > 1.09 x 10^-7 were set to 0.

Title: Supplementary Data 6. DEPICT gene set and tissue enrichment analysis results for PC1.

Description: Displayed significant gene sets and tissues with FDR <0.05.

Title: Supplementary Data 7. Mendelian randomization of rs1453548 effect on tea drinking with outcomes in Neale Lab UKB GWAS.

Description: Results here represent the Wald ratio estimates with 95% confidence intervals and P-values for an increase in cups of tea per day. Only tests with P<0.05 are displayed below.
