# Supplementary material for: Comprehensive genomic analysis of dietary habits in UK Biobank identifies hundreds of genetic associations
Source: Nat Commun. 2020 Mar 19;11:1467. doi: 10.1038/s41467-020-15193-0 (PMC7081342; doi:10.1038/s41467-020-15193-0)
Supplement: Supplementary file 11 — Reporting Summary [file 41467_2020_15193_MOESM11_ESM.pdf]

nature research

Corresponding author(s): Joel Hirschhorn

Last updated by author(s): Jan 23, 2020

Reporting Summary

Nature Research wishes to improve the reproducibility of the work that we publish. This form provides structure for consistency and transparency in reporting. For further information on Nature Research policies, see Authors & Referees and the Editorial Policy Checklist .

Statistics

For all statistical analyses, confirm that the following items are present in the figure legend, table legend, main text, or Methods section.

n/a	Confirmed	
☐	☒	The exact sample size (*n*) for each experimental group/condition, given as a discrete number and unit of measurement
☐	☒	A statement on whether measurements were taken from distinct samples or whether the same sample was measured repeatedly
☐	☒	The statistical test(s) used AND whether they are one- or two-sided<br>*Only common tests should be described solely by name; describe more complex techniques in the Methods section.*
☐	☒	A description of all covariates tested
☐	☒	A description of any assumptions or corrections, such as tests of normality and adjustment for multiple comparisons
☐	☒	A full description of the statistical parameters including central tendency (e.g. means) or other basic estimates (e.g. regression coefficient) AND variation (e.g. standard deviation) or associated estimates of uncertainty (e.g. confidence intervals)
☐	☒	For null hypothesis testing, the test statistic (e.g. *F*, *t*, *r*) with confidence intervals, effect sizes, degrees of freedom and *P* value noted<br>*Give P values as exact values whenever suitable.*
☒	☐	For Bayesian analysis, information on the choice of priors and Markov chain Monte Carlo settings
☒	☐	For hierarchical and complex designs, identification of the appropriate level for tests and full reporting of outcomes
☐	☒	Estimates of effect sizes (e.g. Cohen's *d*, Pearson's *r*), indicating how they were calculated

Our web collection on statistics for biologists contains articles on many of the points above.

Software and code

Policy information about availability of computer code

Data collection	All data was previously collected and is described in detail elsewhere (UK Biobank and previously published GWAS).
Data analysis	Genomic analysis was conducted with BOLT-lmm v.2.3.2, LD score regression v1.0.0, DEPICT, FINEMAP v1.3, LDstore v1.1, SNPsnap, and LocusZoom stand-alone package for data visualization. R v3.4.0 base functions and the following packages were used for phenotype derivation and additional data analysis: Aberrant, MendelianRandomization, TwoSampleMR, and ComplexHeatmap.

For manuscripts utilizing custom algorithms or software that are central to the research but not yet described in published literature, software must be made available to editors/reviewers. We strongly encourage code deposition in a community repository (e.g. GitHub). See the Nature Research guidelines for submitting code & software for further information.

Data

Policy information about availability of data

All manuscripts must include a data availability statement. This statement should provide the following information, where applicable:

- Accession codes, unique identifiers, or web links for publicly available datasets
- A list of figures that have associated raw data
- A description of any restrictions on data availability

All 170 derived dietary habits will be returned and shared through UK Biobank and all GWAS results for the 143 significantly heritable dietary habits will be made publically available on the Type 2 Diabetes Knowledge Portal (http://www.type2diabetesgenetics.org/) upon publication.

Field-specific reporting

Please select the one below that is the best fit for your research. If you are not sure, read the appropriate sections before making your selection.

Life sciences study design

All studies must disclose on these points even when the disclosure is negative.

Sample size	Sample size was based on the complete set of European individuals with both genotyping and food frequency questionnaire information in UK Biobank.
Data exclusions	Individuals that were excluded from analysis were based on poor genetic information or other phenotypic factors that would confound dietary analysis, and is described in the detail in the Methods section.
Replication	GWAS results were obtained from a single large homogeneous population analysis in UK Biobank. Multiple testing correction was used where appropriate, and a strict Bonferroni-adjusted multiple testing threshold was supplied in text for evaluation of significance of top loci. Replication of the causal relationship between education and dietary pattern PC1 was undertaken in two ways using non-overlapping samples.
Randomization	Association analysis was performed on all individuals together adjusting for age, sex, and genetic PCs.
Blinding	Standard genotype and phenotype quality control steps were undertaken and all individuals were analyzed simultaneously for each dietary habit GWAS, such that blinding is not relevant to this study.

Reporting for specific materials, systems and methods

We require information from authors about some types of materials, experimental systems and methods used in many studies. Here, indicate whether each material, system or method listed is relevant to your study. If you are not sure if a list item applies to your research, read the appropriate section before selecting a response.

Materials & experimental systems

n/a	Involved in the study	
☒	☐	Antibodies
☒	☐	Eukaryotic cell lines
☒	☐	Palaeontology
☒	☐	Animals and other organisms
☐	☒	Human research participants
☒	☐	Clinical data

Methods

n/a	Involved in the study	
☒	☐	ChIP-seq
☒	☐	Flow cytometry
☒	☐	MRI-based neuroimaging

Human research participants

Policy information about studies involving human research participants

Population characteristics	All analysis was conducted using the UK Biobank resource, described in detail elsewhere. Briefly, the UK Biobank is an adult population cohort (N~500K) from the UK with deep phenotyping and genetic information collected between 2006-2010. Details on our use of UK Biobank participant data are described in detail in the Methods section.
Recruitment	UK Biobank recruitment aimed for a widely generalizable population, though similar to other population cohorts and as has been noted by others, is subject to healthy participant bias in which the sample tends to have reduced disease rates and higher socioeconomic status than a true population sample.
Ethics oversight	Ethics oversight of UK Biobank is described elsewhere. All research conducted herein was done using UK Biobank application 11898.

Note that full information on the approval of the study protocol must also be provided in the manuscript.
